# Discovery of High Affinity Receptors for Dityrosine through Inverse Virtual Screening and Docking and Molecular Dynamics

**DOI:** 10.3390/ijms20010115

**Published:** 2018-12-29

**Authors:** Fangfang Wang, Wei Yang, Xiaojun Hu

**Affiliations:** 1 School of Life Science, Linyi University, Linyi 276000, China; 2 Department of Microbiology, Biomedicine Discovery Institute, Monash University, Clayton, VIC 3800, Australia; zhuxiaoqing88@163.com; 3 Arieh Warshel Institute of Computational Biology, the Chinese University of Hong Kong, 2001 Longxiang Road, Longgang District, Shenzhen 518000, China

**Keywords:** dityrosine, inverse virtual screening, molecular docking, molecular dynamics

## Abstract

Dityrosine is the product of oxidation that has been linked to a number of serious pathological conditions. Evidence indicates that high amounts of dityrosine exist in oxidized milk powders and some milk related foodstuffs, further reducing the nutritional value of oxidized proteins. Therefore, we hypothesize that some receptors related to special diseases would be targets for dityrosine. However, the mechanisms of the interaction of dityrosine with probable targets are still unknown. In the present work, an inverse virtual screening approach was performed to screen possible novel targets for dityrosine. Molecular docking studies were performed on a panel of targets extracted from the potential drug target database (PDTD) to optimize and validate the screening results. Firstly, two different conformations cis- and trans- were found for dityrosine during minimization. Moreover, Tubulin (αT) (−11.0 kcal/mol) was identified as a target for cis-dityrosine (CDT), targets including αT (−11.2 kcal/mol) and thyroid hormone receptor beta-1 (−10.7 kcal/mol) presented high binding affinities for trans-dityrosine (TDT). Furthermore, in order to provide binding complexes with higher precision, the three docked systems were further refined by performing thermo dynamic simulations. A series of techniques for searching for the most stable binding pose and the calculation of binding free energy are elaborately provided in this work. The major interactions between these targets and dityrosine were hydrophobic, electrostatic and hydrogen bonding. The application of inverse virtual screening method may facilitate the prediction of unknown targets for known ligands, and direct future experimental assays.

## 1. Introduction

Proteins represent major targets for free radicals arising from oxygen reduction in vivo, which would be generated as a result of normal cellular metabolism and external stimulus, including environmental agents, such as metal ions and radiation. The relative oxidation reactions would modify amino acid residues, altering the structure and function of proteins, and further forming protein cross-links [1,2]. Moreover, the resulting changed proteins participate in damaging reactions and signal induction as a new reaction entity [3].

During food processing and storage, oxidized protein products (OPPs), such as protein carbonyls, methionine sulfoxide, and dityrosine, would be generated as a response to heat treatment, oxygen and light exposure, further leading to a decrease of product quality, safety, and nutritional value [4,5]. Studies have proven that OPPs have been tested in plasma and accumulated in diverse disorders, such as diabetes mellitus, metabolic syndrome, atherosclerosis, coronary artery disease, and chronic kidney disease [6,7,8]. Furthermore, OPPs also play an important role in a wide range of age-related diseases, including Parkinson′s disease, senescence, and asthma [9,10,11]. The above studies indicate that OPPs ingested from food systems could accumulate in cells, then have influence on human bodies, leading to the occurrence of some diseases. 

In particular, tyrosine as the most sensitive residue among the aromatic amino acid residues is the potential target of oxidative modification. Moreover, an oxidized product 1, 3-dityrosine is formed through protein dimerization. Dityrosine is a highly fluorescent compound that is resistant to protease activity and acid hydrolysis [12]. Furthermore, dityrosine is a useful marker for assessing oxidative damage to proteins [13,14], which can be generated during a normal physiological process in specialized cases and also as a result of exposure to environmental agents (ultraviolet irradiation, radicals, NO_2_, and lipid hydroperoxides). Dityrosine has been found in the elastic ligaments of insects [15], the cell walls of *Candida albicans* and *Saccharomyces cerevisiae* [16,17], and the fertilization envelope of the sea urchin egg *Stronglycocentrotus purpuratus* [18]. Additionally, dityrosine has been detected in the hydrolysates of some structural proteins [19,20], insoluble proteins derived from human cataractous lenses [21], elastin, collagen [22], and the storage form of thyroglobulin [23]. In addition, dityrosine is also linked to a number of serious pathological cases, such as brain tissues from Parkinson′s disease [24], and aortic tissues of hyperglycaemic animals [25]. 

Up to now, although more than 245 articles have been focused on studying dityrosine, considerable process has been made in dityrosine preparation, isolation, analysis, and the role for dityrosine crosslinking of some diseases. Based on the previous publications, it is assumed that dityrosine may result in potential damage to food nutrition and the human body, moreover, some related proteins might be the targets for dityrosine. Therefore, in this paper, an in silico inverse virtual screening approach was performed on dityrosine in an attempt to evaluate the possible interaction between dityrosine and targets that have been recognized as participants in different diseases and find possible binding conformations to direct experimental assays.

## 2. Results and Discussion

### 2.1. Inverse Virtual Screening

The inverse virtual screening was done by AutoDock Vina, which has been proven to be efficacious in predicting the binding poses and energies, and particularly developed for parallel computing [26]. Therefore, in the present work, we chose this method for large inverse virtual screening studies. 

#### 2.1.1. For Cis-Dityrosine (CDT)

After inverse virtual screening, the scoring function results of the compounds are listed in Tables S1–S17. The highest docking score (lower than −8.0 kcal/mol) in the group of binding proteins, as shown in Table S1, is formed between CDT and intestinal fatty acid binding protein (IFABP), with binding score of −9.1 kcal/mol. 

In the class of nuclear receptors (Table S2), Lac Repressor (LacR) exhibits the best binding affinity for CDT, possessing score of −9.1 kcal/mol, then, Peroxisome proliferator activated receptor delta, Retinoic acid receptor RXR-alpha, Nuclear Vitamin D Receptor, Thyroid hormone receptor Alpha-1, Hepatocyte nuclear factor 4-gamma, Cellular Retinoic-Acid-Binding Protein Type II, and Liver X receptor alpha might interact with CDT, with binding scores of −8.7 kcal/mol, −8.4 kcal/mol, −8.3 kcal/mol, −8.2 kcal/mol, −8.2 kcal/mol, −8.0 kcal/mol and −8.0 kcal/mol, respectively. 

The targets involved in transport proteins (Table S3), receptors (Tables S4 and S12), Monoclonal Antibodies (Table S5), Factor, Regulator and Hormones (Table S6), Structural Proteins (Table S7), Signaling Proteins (Table S8), Ion Channels (Table S9), Lipid Binding Protein (Table S11), other Enzymes (Table S13) with the best binding scores are Cytochrome C2 (−8.7 kcal/mol), Actin, alpha skeletal muscle (−8.9 kcal/mol), Immunoglobulin lambda Light Chain Dimer (Mcg) (−8.9 kcal/mol), Gonadotropin alpha subunit (−8.6 kcal/mol), Tubulin alpha chain (−8.1 kcal/mol), Ran-GPPNHP-RanBP1-RanGAP (−9.0 kcal/mol), Voltage-Gated Potassium Channel (−8.6 kcal/mol), KES1 protein (−8.6 kcal/mol), and Aldehyde oxidoreductase (−9.9 kcal/mol), respectively. 

Among the circadian clock-related proteins (Table S14), the targets CLK1, CLK2, CLK4 and CK II alpha′ exhibit higher binding scores toward CDT, with values of −9.1 kcal/mol, −8.3 kcal/mol, −8.0 kcal/mol, and −8.0 kcal/mol, respectively. 

In addition, the good interaction in insulin receptor pathway proteins (Table S15) is formed between PKA C-alpha and CDT, with value of −8.9 kcal/mol. 

The serum proteins (Table S16) with the best docking scores are SL-2, ADAM-TS 1, ADAM-TS 5, ADORA2A, MMP-13, Topo1, ADAM 17 and AE 1, with binding scores of −8.6 kcal/mol, −8.4 kcal/mol, −8.3 kcal/mol, −8.3 kcal/mol, −8.2 kcal/mol, −8.2 kcal/mol, −8.1 kcal/mol, and −8.1 kcal/mol, respectively. 

The other positive interactions are obtained in the breast cancer proteins, between CDT and Beta-2 adrenoceptor, TOP2A, MMP−9, MMP-16, CHEK2, Stromelysin-1 and LAR, having binding affinities of −9.8 kcal/mol, −9.3 kcal/mol, −8.7 kcal/mol, −8.6 kcal/mol, −8.4 kcal/mol, and −8.2 kcal/mol, respectively. 

#### 2.1.2. For Trans-Dityrosine (TDT)

The results of inverse virtual screening for TDT are shown in Tables S18–S34. The AutoDock Vina method gives the best performance in the group of binding proteins (Table S18), retrieving Streptavidin, Intestinal Fatty Acid Binding Protein, Annexin III, Uteroglobin, with binding scores of −8.7 kcal/mol, −8.5 kcal/mol, −8.1 kcal/mol, and −8.1 kcal/mol, respectively. 

For the group of nuclear receptors (Table S19), thyroid hormone receptor beta-1 (TRβ1) exhibits the best binding score for TDT, possessing binding affinity of −9.0 kcal/mol. 

The targets of interest among transport proteins (Table S20), receptors (Tables S21 and S29), Monoclonal Antibodies (Table S22), Factor, Regulator and Hormones (Table S23), Structural Proteins (Table S24), Signaling Proteins (Table S25), Ion Channels (Table S26), Lipid Binding Protein (Table S28), and other Enzymes (Table S30) are Transferrin, Metabotropic Glutamate Receptor 2, Immunoglobulin lambda Light Chain Dimer (Mcg), Heat Shock 70 kDa protein 8, Myosin light chain kinase family, Ran-GPPNHP-RanBP1-RanGAP, Voltage-Gated Potassium Channel, KES1 protein and Lanosterol Synthase (LS), with values of −9.0 kcal/mol, −9.8 kcal/mol, −9.0 kcal/mol, −8.7 kcal/mol, −9.4 kcal/mol, −9.2 kcal/mol, −8.5 kcal/mol, −9.1 kcal/mol, and −10.1 kcal/mol, respectively. 

The performance of inverse virtual screening for circadian clock related proteins is given in Table S31, among the ten receptors, CK II alpha′ (−8.7 kcal/mol), CLK1 (−8.5 kcal/mol), CLK2 (−8.2 kcal/mol), CLK4 (−8.1 kcal/mol) and CK II alpha (−8.0 kcal/mol) give the binding score lower than −8.0 kcal/mol. 

As shown in Table S32, eleven targets are favorable for binding insulin receptor pathway proteins toward TDT, thereinto, the best docking affinity value is found for the complexes formed by TDT and PKA C-alpha, with docking score of −9.7 kcal/mol. 

Several targets, SL-2, Topo1, ADAM-TS 5, HLA-A, ADAM-TS 1, ADAM 22, MMP-13, HLA-B8, ADAM 17, ADAM-TS 4, AE 1 and ADORA2A from serum proteins (Table S33) have shown excellent docking scores, with values of −9.1 kcal/mol, −8.9 kcal/mol, −8.8 kcal/mol, −8.6 kcal/mol, −8.6 kcal/mol, −8.4 kcal/mol, −8.3 kcal/mol, −8.2 kcal/mol, −8.2 kcal/mol, −8.2 kcal/mol, −8.1 kcal/mol, and −8.1 kcal/mol, respectively. 

The breast cancer proteins (Table S34) with good docking affinities in silico toward TDT are Beta-2 adrenoceptor, MMP-16, MMP−9, DNA topoisomerase II and CHEK2, with binding scores of −9.5 kcal/mol, −9.1 kcal/mol, −8.8 kcal/mol, −8.5 kcal/mol and −8.2 kcal/mol, respectively. 

### 2.2. Refinement Docking

For further screening, a refinement docking procedure was carried out on dityrosine/targets complexes with binding scores lower than −8.0 kcal/mol (the results obtained from inverse virtual screening). 

#### 2.2.1. For CDT

**Figure 1:**
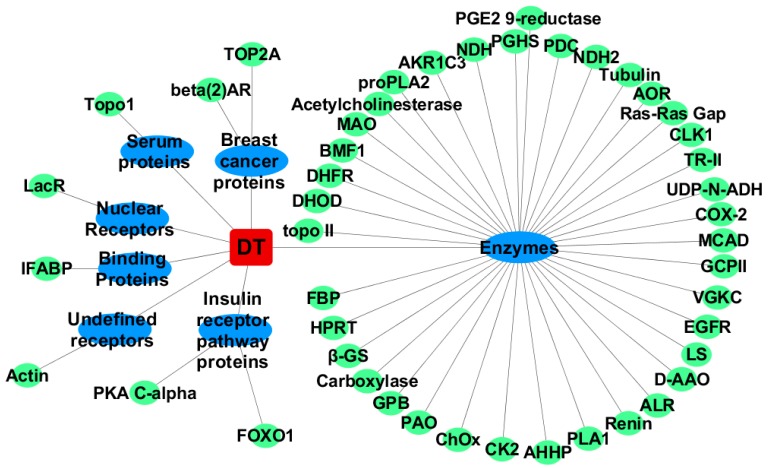
The network of CDT and the screened targets.

After refinement docking, the scoring function results of the complexes with binding affinities lower than −9.0 kcal/mol are shown in Table S35 and Figure 1. As expected, the ligand-target complex with the highest docking score is αT-CDT, with an average of −11.0 ± 0.0 kcal/mol, which belongs to the group of enzymes. In addition, Aldehyde oxidoreductase, Glutamate carboxypeptidase II and Beta-2 adrenoceptor also display excellent binding scores for CDT, with docking scores of −9.9 kcal/mol, −9.9 kcal/mol, and −9.8 kcal/mol, respectively. 

#### 2.2.2. For TDT

**Figure 2:**
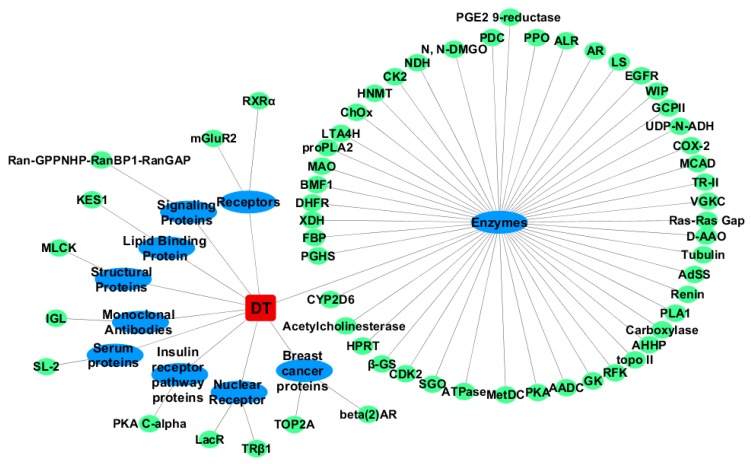
The network of TDT and the screened targets.

Similar to CDT, αT also shows the highest binding affinity for TDT, with an average score of −11.2 ± 0.0 kcal/mol (Table S36 and Figure 2). Moreover, Leukotriene A4 hydrolase (LTA4H), TRβ1, LS and Aldose Reductase (AR), with docking affinity values of −10.2 ± 0.0, −10.0 ± 0.0, −10.1 ± 0.0, and −10.0 ± 0.0 kcal/mol, respectively, also show promising affinities for TDT.

Considering the results of refinement docking, a conclusion would be drawn that both CDT and TDT can bind to the binding sites of targets derived from different groups of receptors.

### 2.3. Validation

To further validate the rationality of the docking procedure, and deeply screen the probable dityrosine binding receptors, a process of validation was performed on complexes with binding affinity lower than −9.0 kcal/mol, prior to docking analysis, a redocking process of the cocrystallized ligands into related receptors was performed, and a docking score was obtained. Simultaneously, dityrosine was docked into the related receptors, and the receptor would be preserved when dityrosine scored better docking affinity than the co-crystallized ligands. Ideally, complexes depicting binding affinities better than those generated for the crystal structures are likely to occur at a molecular level. 

#### 2.3.1. For CDT

After validation, the scoring function results of the eligible receptors are shown in Table S37. Accordingly, binding proteins, enzymes, insulin receptor pathway proteins and breast cancer proteins are involved in CDT binding. The best docking affinity values are found for the complexes formed by CDT and αT (Enzymes), with binding score of −11.0 kcal/mol, which is lower than the complex TXL-αT (−9.1 kcal/mol). 

#### 2.3.2. For TDT

The results of validation for TDT are listed in Table S38. Similar to CDT, the deeply screened targets belong to the group of enzymes, insulin receptor pathway proteins and breast cancer proteins. In addition, nuclear receptor (TRβ1) is also connected to TDT binding. Furthermore, the target involved in enzymes with the best binding score is αT with a value of −11.2 kcal/mol, better than the co-crystallized ligand (−9.1 kcal/mol). 

The validation results suggest that CDT and TDT structures resulting from the docking procedure possess different binding receptors, further illustrating that different conformations of dityrosine would choose diverse targets, then showing different physiological effects. 

### 2.4. RMSD (Root Mean Square Deviation)/RMSF (Root Mean Square Fluctuation) Analysis

**Figure 3:**
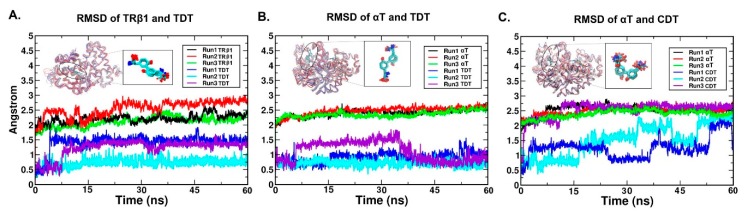
The RMSD of protein backbone atoms and heavy atoms of ligands along the production phase simulations. The RMSD of the protein backbone atoms and ligand atoms from TRβ1-TDT, αT-TDT and αT-CDT system are shown per panel (**A**), (**B**) and (**C**). Different replicates were displayed by the colour lines indicated in each legend box. The superposed proteins by tube were made by superposing 60 conformations (20 conformations per replicate) with 100 ps interval along the production phase. The degree of the dispersion of the tubes indicated the protein dynamics along simulation time series (‘red to blue′ represents ‘0 ns to 60 ns′). The ligands superposed with the same method were shown nearby and draw by sticks.

Molecular dynamics (MD) simulations were performed on the three docked systems with the purpose of collecting the more reliable binding conformations. The RMSD of the protein backbone atoms indicated that the proteins experienced little turbulence at the first 7.5 ns and stabilized during 7.5–60 ns (2.25 ± 0.15 Å for TRβ1, 2.43 ± 0.05 Å for αT bound with TDT, 2.62 ± 0.09 Å for αT bound with CDT) along all the production phase (Figure 3). The RMSD of the TDT and CDT had lower fluctuations compared to the proteins, and all stabilized during the last 15 ns in all systems. (Figure 3). Interestingly, CDT in αT model fluctuated a lot compared to TDT in the other two systems, especially the strong fluctuation of the NHCO_2_^−^ group (Figure 3C). The different binding poses other than the docked one might be expected in the production phase simulation.

**Figure 4:**
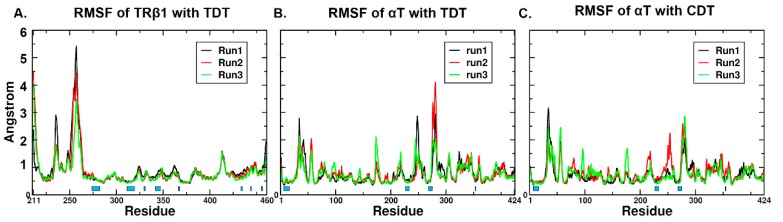
The RMSF of protein backbone atoms in all production phase simulations. The RMSF of backbone atoms in TRβ1-TDT, αT-TDT and αT-CDT system are shown per panel (**A**), (**B**) and (**C**). The residues involved in close contact with ligands (residues within 4.5 Å around the ligands) were marked underneath with blue boxes per panel.

The residue flexibility of the proteins was further checked by RMSF (Figure 4). The high fluctuations were only found from the loops of TRβ1 (K211, average 2.78 Å, Q235 average 2.98 Å, Q252-A258, average 3.32 Å) and αT (G34-Q35, average 2.56 Å, L248, average 2.87 Å, A281, average 2.94 Å). The residues involved in the binding of ligands resulted in smaller average fluctuations (0.42 Å for TRβ1-TDT, 0.45 Å for αT-TDT, 0.49 Å for αT-CDT), suggesting the reasonable conformations were obtained from the docking procedure. However, we observed higher residue fluctuations in the ligand-contact residues of loop^F270-Q279^ (3.41 Å for αT-CDT, 2.82 Å for αT-TDT) in αT systems. Since the loop ^F270-Q279^ was located at the rim of the pocket, it might account for the fluctuation of CDT because of the synergetic movements between the receptor and the ligand. 

### 2.5. The Stable Binding Conformation Analysis

#### 2.5.1. The Stable Binding Conformation of TRβ1-TDT

**Figure 5:**
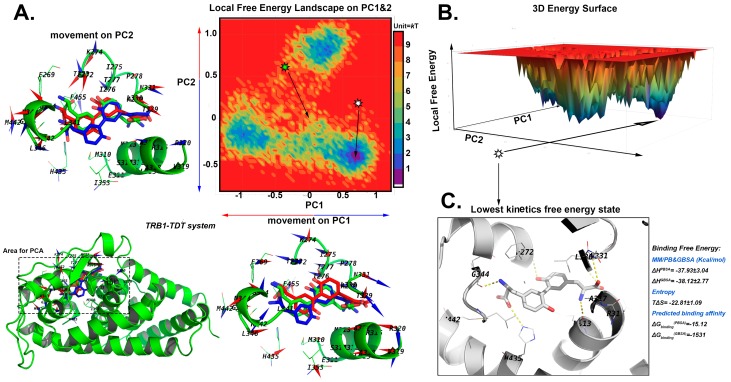
The search for the lowest free energy poses in TRβ1-TDT MD system by local free energy landscape (LFEL) and Molecular Mechanics MM/GB and PBSA calculations. (**A**) The 2D LFEL heat map. Green star shows the docked conformation with the coordinate (0, 0), and is viewed as a reference structure. The highest local free energy was defined as 10 kT, which can barely be sampled from all the conformational space. The bottom left shows the areas for which Principal Component Analysis (PCA) calculations were carried out. The docked pose is in green. The movements on the PC1 and 2 vectors from the production phase simulations are shown as porcupine plots alongside with the axis. The scales of arrows are proportional to the scale of movements observed along each PC. The movements towards positive direction of PC are in blue, and that of negative direction are in red. TDT are in sticks; blue and red sticks represent the minimum and maximum conformations observed by the PC vectors and surrounding residues are shown as cartoon. The docked pose is coloured in green. (**B**) The converted 3D surface of FEL with the same colour code as shown in panel A. (**C**) The collected state with lowest kinetic free energy. The protein is in white cartoon, TDT are shown by sticks, and the nearby residues are shown as white lines. The hydrogen bonds are indicated by yellow dashes. The collected conformation and the nearby 50 frames were used for calculating the binding free energy, which are displayed next to panel C.

The pocket and TDT in TRβ1 has little movements, and small scale of movements were found along principal component PC1 and 2 (Figure 5A). Interestingly, the pose with lowest local free energy (0.38 kT, Figure 5B) were found at a state in which the TDT was located deeper in the centre of the protein, and F272-I275 of Helix 5 moved up by 1.1 Å, and H6 (L341–G344) shifted outward by 1.2 Å compared to the docked pose, suggesting that the system reached a most stable conformation by enlarging the pocket for harbouring TDT and eliminated the bumps caused by the rigid docking process. The binding free energy of TRβ1-CDT is −15.12 kcal/mol with Poisson−Boltzmann Surface Area (PBSA) and −15.31 kcal/mol with Generalized Born Surface Area (GBSA), which was a little higher than predicted with the docking result.

#### 2.5.2. The Stable Binding Conformation of αT-TDT

**Figure 6:**
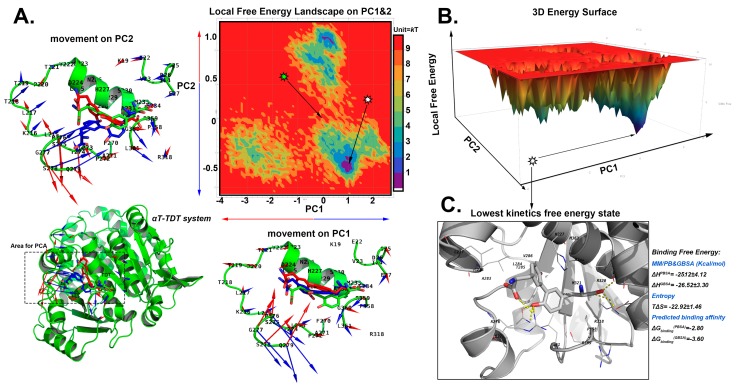
The search for the lowest free energy poses in αT-TDT MD system by local free energy landscape (LFEL) and MM/GB and PBSA calculations. (**A**) The 2D LFEL heat map. The green star shows the docked conformation with the coordinate (0, 0), and is viewed as a reference structure. The highest local free energy was defined as 10 kT, which can barely be sampled from all the conformational space. The bottom left shows the areas for which PCA calculations were carried out. The docked pose is in green. The movements on PC1 and 2 vectors from the production phase simulations are shown as a porcupine plot alongside with the axis. The scales of arrows are proportional to the scale of movements observed along each PC. The movements towards the positive direction of PC are in blue, and that of negative direction are in red. TDT are in sticks; blue and red sticks represent the minimum and maximum conformations observed by the PC vectors, and surrounding residues are shown as cartoon. The docked pose is coloured in green. (**B**) The converted 3D surface of FEL with the same colour code as shown in panel A. (**C**) The collected state with lowest kinetic free energy. The protein is in white cartoon, TDT are shown by sticks, and the nearby residues are shown as white lines. The hydrogen bonds are indicated by yellow dashes. The collected conformation and the nearby 50 frames were used for calculating the binding free energy, which are displayed nearby panel C.

A large movement of β turn 31–32 (A270–V275) was found in the local PCA in the αT-TDT system. The β turn 31–32 can move inward/outward by 2.5 Å (Figure 6A), due to its exposure to the solvent and loose secondary structure. Interestingly, the TDT did not shift much accordingly (around 1.12 Å). The pose with the lowest local free energy was found by the 3D Energy Surface (1.31 kT, Figure 6B). The binding free energy of αT-TDT is −2.80 kcal/mol with PBSA and −3.60 kcal/mol with GBSA.

#### 2.5.3. The Stable Binding Conformation of αT-CDT

**Figure 7:**
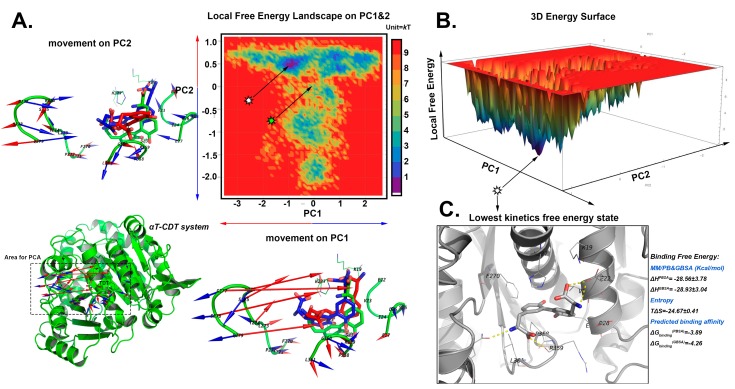
The search of lowest free energy poses in αT-CDT MD system by local free energy landscape (LFEL) and MM/GB and PBSA calculations. (**A**) The 2D LFEL heat map. The green star shows the docked conformation with the coordinate (0, 0), and is viewed as a reference structure. The highest local free energy was defined as 10 kT, which can barely be sampled from all the conformational space. The bottom left shows the areas for which PCA calculations carried out. The docked pose is in green. The movements on PC1 and 2 vectors from the production phase simulations are shown as a porcupine plot alongside with the axis. The scales of arrows are proportional to the scale of movements observed along each PC. The movements towards the positive direction of PC are in blue, and that of the negative direction are in red. CDT are in sticks; blue red sticks represent the minimum and maximum conformations observed by the PC vectors and surrounding residues are shown as cartoon. The docked pose is coloured in green. (**B**) The converted 3D surface of FEL with the same colour code as shown in panel A. (**C**) The collected state with lowest kinetic free energy. The protein is in white cartoon, CDT are shown by sticks, and the nearby residues are shown as white lines. The hydrogen bonds are indicated by yellow dashes. The collected conformation and the nearby 50 frames were used for calculating the binding free energy, which are displayed nearby panel C.

**Figure 8:**
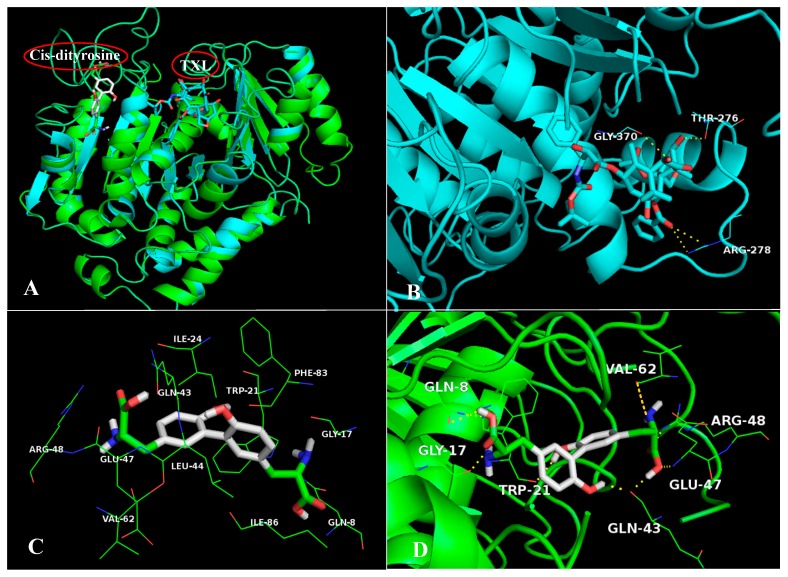
(**A**) Superposition between the crystallographic structures of the complex 1UTB/TXL with the resultant docking pose of 1UTB/CDT. Crystallographic structure and docking structure are represented in blue and green, respectively. (**B**) The enlargement for ligand TXL in the binding site of 1UTB, which is displayed as stick, hydrogen bonds are shown as dotted yellow lines, and the non-polar hydrogens were removed for clarity. (**C**) The active site amino acid residues around CDT. (**D**) The enlargement for ligand CDT in the binding site of 1UTB, which is displayed as stick, hydrogen bonds are shown as dotted yellow lines, and the non-polar hydrogens were removed for clarity.

Large synergetic movements between CDT and β turn 31–32 are found by the PC1 and PC2. With the swing movement (along PC1 axis) of β turn 31–32, the NHCO_2_^−^ group can either detach (blue direction) or attach (red direction) to the β turn 31–32 (Figure 7A). The most stable binding conformation was found from the LFEL map (0.779 kT, Figure 8A,B) as the NHCO_2_^−^ group interacts with β turn 31–32, and the binding pocket is more compact than the docked conformation with two more hydrogen bonds found between Q279:O-CDT:N1 and R359:O-CDT:O2. The binding free energy of αT-CDT is −3.89 kcal/mol with PBSA and −4.26 kcal/mol with GBSA.

### 2.6. Conformational Analysis

After MD simulation, the complexes were further visually inspected for binding types and specific interacting residue.

#### 2.6.1. For CDT

The results from ligand-receptor interaction analyses (Table S39) reveal that the interactions found in CDT/target complexes are mainly hydrophobic and electrostatic, as well as hydrogen bond donor and hydrogen bond acceptor. As shown in Table S39 and Figure 3C, the highest-scored receptor-αT has interactions with residues Gln8, Trp21, Ile24, Leu44, Glu47, Arg48, Val62, Phe83, and Ile86. For complex αT/TXL, the model of possible interacting hydrogen bonds is depicted in Figure 8B. The ligand TXL is hydrogen bonded with amino acid residues Thr276, Arg278 and Gly370, while compound CDT has hydrogen bond interaction with residues Gln8, Gly17, Trp21, Gln43, Glu47, Arg48 and Val62 (Figure 8D), accounting for the better docking score and probable elevated binding activity. Evidently, CDT and TXL are buried in different binding sites of the receptor αT (shown in Figure 8A) with different surrounding residues because the two compounds differ in structure and volume. In particular, the volume of ligand TXL is larger, thus it binds to a wider cavity. Conversely, CDT chooses to locate in a slender and narrowed cavity for enhanced activity. Therefore, the two compounds might bind to different binding sites and possess different orientations, further suggesting that a second binding site might exist on the surface of the receptor αT, showing a diverse function and physiological phenomenon. 

#### 2.6.2. For TDT

**Figure 9:**
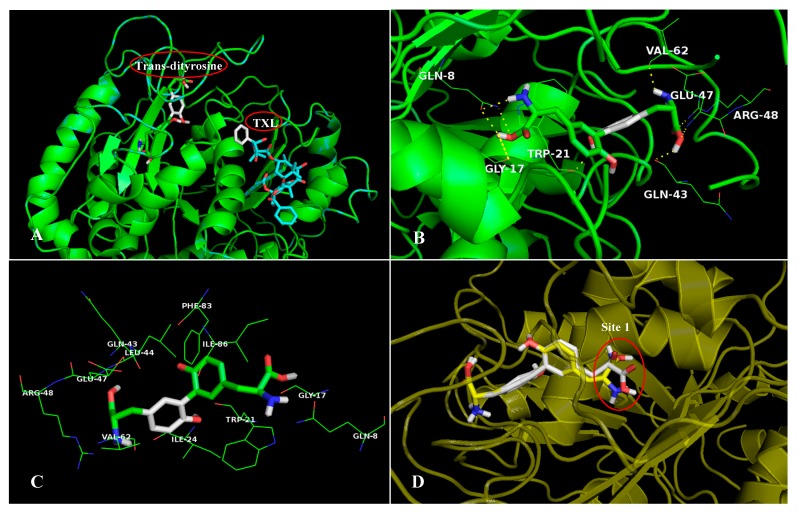
(**A**) Superposition between the crystallographic structures of the complex 1UTB/TXL with the resultant docking pose of 1UTB/TDT. Crystallographic structure and docking structure are represented in blue and green, respectively. (**B**) The enlargement for ligand TDT in the binding site of 1UTB, which is displayed as stick, hydrogen bonds are shown as dotted yellow lines, and the non-polar hydrogens were removed for clarity. (**C**) The active site amino acid residues around TDT. (**D**) Superposition between the resultant docking pose of 1UTB/CDT with the docking pose of 1UTB/TDT, which are represented in white and yellow, respectively.

As illustrated in Table S40, the TDT has hydrophobic, electrostatic hydrogen bonding interactions to the receptors with the highest docking scores, suggesting the importance of these interactions in TDT binding in screened targets. For instance, contact residues participating in the interaction between TDT and αT are principally Gln8, Gly17, Trp21, Ile24, Gln43, Leu44, Glu47, Arg48, Val62, Phe83 and Ile86 (Figure 9C). Compound TDT establishes hydrogen bond interactions with the same residues of the pocket occupied by CDT (Gln8, Gly17, Trp21, Gln43, Glu47, Arg48 and Val62), as shown in Figure 9B, illustrating that the two different configurations of dityrosine bind to the same active pocket. Nevertheless, TDT and TXL are not overlapped in the same pocket (Figure 9A), which is similar to CDT, indicating an alternative strategy of binding. However, there are also some differences between CDT and TDT. It is evidenced that the group at Site 1 for CDT turns ~90° relative to that of TDT; in mechanistic terms, we think that the trans-conformation is more stable than the cis-conformation. However, the two different conformations might exist simultaneously in nature, and transform each other, displaying dissimilar functions. 

Furthermore, αT is a popular target for numerous small ligands which would change microtubule dynamics, then resulting in cell cycle arrest and apoptosis [27]. In addition, evidence also indicates that misfolded αT monomers are highly toxic and would quickly degrade, leading to Parkinson′s disease [28]. This might hint that dityrosine could be responsible for mediating biochemical responses, such as cancer and Parkinson′s disease. 

**Figure 10:**
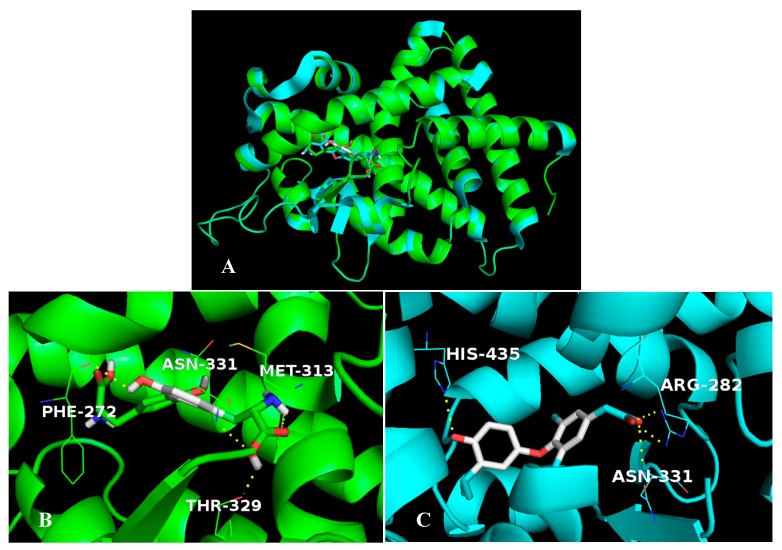
(**A**) Superposition between the crystallographic structures of the complex 1NAX/IH5 with the resultant docking pose of 1NAX/TDT. Crystallographic structure and docking structure are represented in blue and green, respectively. (**B**) The enlargement for ligand TDT in the binding site of 1NAX, which is displayed as stick, hydrogen bonds are shown as dotted yellow lines, and the non-polar hydrogens were removed for clarity. (**C**) The enlargement for ligand IH5 in the binding site of 1NAX, which is displayed as stick, hydrogen bonds are shown as dotted yellow lines, and the non-polar hydrogens were removed for clarity.

Shown in Figure 10 is another TDT possible binding receptor thyroid hormone receptor β1 (TRβ1). In order to identify the structural similarity and divergence of the active sites between TDT and IH5, the docking mode of TDT/1NAX is superimposed on the structure of IH5/1NAX, as shown in Figure 10A, the two compounds bind to the same pocket. As listed in Table S40, compound TDT is secured by residues Phe272a, Ile275a, Ile276a, Ala279a, Met310a, Met313a, Arg320a, Thr329a, Leu330a, Leu341a, Gly344a, and Leu346a. Compared with ligand IH5, TDT maintains the hydrogen bond interaction to Asn331 (Figure 10B,C), suggesting the importance of this interaction in ligand binding to TRβ1. The other ligand-receptor hydrogen bond interactions have diminished, which are compensated by alternative hydrogen bond interactions to residues Phe272, Met313 and Thr329. 

Additionally, TRβ1 is a ligand-dependent transcription factor, regulating gene expression located in skeletal, intestinal and cardiac muscles, the liver and the central nervous system, and controlling heart rate, and triglyceride and cholesterol levels [29,30]. Thus, the pharmacological actions of TRβ1 are related to obesity, hypercholeaterolemia and diabetes [31,32,33,34]. Although Baudry et al. [35] showed that dityrosine formation is related to a physiological process, thyroid hormone synthesis, mechanisms involving this receptor have not yet been considered. The theoretical molecular docking applied in this work showed that TDT might interact with nuclear receptor TRβ1, which was also proven in research by Li et al. [36], revealing that oxidized casein impaired the antioxidant defense system and induced hepatic and renal injury in mice. 

Besides the above discussed receptors, some other receptors are also targets for CDT and TDT. The binding scores for these targets toward CDT/TDT are higher than −10.0 kcal/mol, and lower than −9.0 kcal/mol. In addition, the docking scores are better than those co-crystallized ligands, suggesting that these receptors would be weak targets for CDT/TDT, shown in Tables S39 and S40. 

## 3. Materials and Methods 

### 3.1. Preparation of Ligand and Target Structures

**Figure 11:**
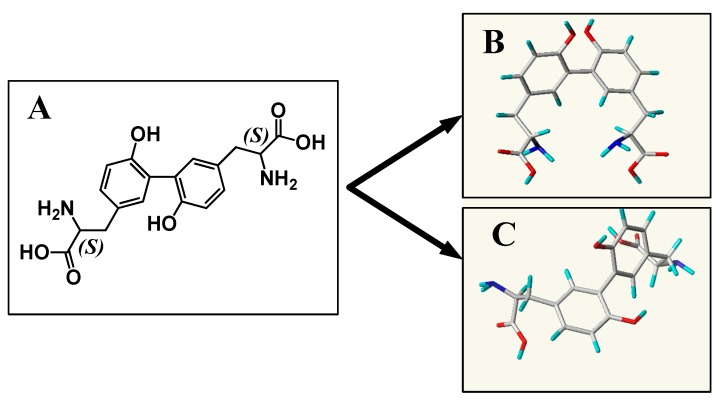
(**A**) Chemical structure of dityrosine. (**B**) Three-dimensional structure of CDT. (**C**) Three-dimensional structure of TDT.

Dityrosine structure was downloaded from PubChem Compound of NCBI. The three-dimensional structure was optimized by molecular dynamics simulations, which were carried out using the NAMD2.9 MD package [37]. Partial charges were calculated with AM1 in MOPAC7 and fitted into the AM1-bcc type of charge. The generalized AMBER force field (GAFF) [38] was assigned by using Antechamber [39]. During the procedure of minimization, two different conformations were identified, as shown in Figure 11 Then, the output file was translated to pdbqt format using AutoDockTools [40]. 

A text-mining step was firstly performed to select targets that may have connection with dityrosine in scientific reports, however, few studies have been committed to dityrosine-target systems. Therefore, the PDTD database, a useful resource for identifying receptors for active compounds or existing drugs [41] consisting of 1044 kinds of potential drug target molecules, was chosen as our candidate screening set. The database is related to diverse diseases, biological functions and signaling pathways, incorporating enzymes, receptors, monoclonal antibody, regulatory factors and hormones. Then, polar hydrogen atoms and Kollman charges were assigned to the potential drug targets, ensuring that the pronation states were right, and water molecules were removed from the crystal structures. 

### 3.2. Inverse Virtual Screening

For molecular docking, a grid box in AutoDockTools was used to determine the docking coordinates [42]. All dockings were confined in a grid box with dimensions of 60 × 60 × 60 Å, grid spacing of 0.375 Å, and the grid center was designated according to the original ligands embedded in the receptors [43]. Molecular docking simulations were performed using Autodock-Vina software, which combines knowledge-based potentials and empirical scoring functions and Iterated Local Search global optimizer algorithm for the local optimization [44]. It is widely used due to its speed and accuracy for docking calculations [45]. 

An initial screening procedure was performed to rank the receptors as dityrosine targets, and the parameters in the configuration file were set as follows: energy_range 1.5, num_ modes 20, and exhaustiveness 25. 

### 3.3. Refinement Docking Experiments

A refinement experiment was further carried out on receptors with lower docking scores (lower than −8.0 kcal/mol). In this step, energy range of 1.5, number of modes of 50, and an exhaustiveness of 100 were employed. 

### 3.4. Validation

The validation procedure requires the identification of molecules with known activity against the target. Thus, to validate the reasonability of molecular docking and further screen the dityrosine-target complexes with higher binding scores, molecular docking simulations were performed on complexes presenting high affinity scores lower than −9.0 kcal/mol using the screened targets and the co-crystallized ligands. During molecular docking, the original ligands, other substructures, and water molecules were removed from the crystal structures, and the relevant co-crystallized ligands and targets were prepared and docked using the same parameters as that described in Section 3.2, Section 3.3 and Section 3.4. 

The resulted docking scores were employed for further comparison, dityrosine-target complexes with binding scores higher than those co-crystalized ligand-target complexes would be retained. In addition, a detailed conformational analysis based on dityrosine-target and co-crystalized ligand-target was carried out to examine the docking pose predicted by AutoDock Vina with respect to the experimental data. 

### 3.5. MD Process

The docked poses of TDT and CDT with the receptors were used as the starting conformations for the MD simulations. The inputs for generating the parameters of ligands (TDT and CDT) were created by using Antechamber of Ambertools 18 [46]. The RESP charges [47] of the ligands were obtained by calculating the Density Function Theory (DFT) function of /6-31G** from Gaussian 09 [48,49]. The bond constants were obtained from Amber GAFF. In the refined homology model of alpha-tublin (αT), G2P was modelled as in the electron crystallography structure of αT (PDBID: 1TUB) [50]. The generation of G2P parameters followed the same approach as carried onto the ligands. Importantly, the dihedral angles of O1-P1-O2-P2 and O2-P2-C3A-P3 were frozen in the quantum mechanism calculation of the optimizing step to prevent the self-protonation of G2P. The proteins of the 3 systems were then defined by AMBER 14SB force field [51], and counter ions (Cl- and Na+) were added in the systems to neutralise the overall charges of the systems. Following this, the periodic solvent box with Å TIP3P water layers was added in each system [52]. The final system of TRB1-TDT was 89.27 × 77.89 × 72.48 Å^3^ (40,815 atoms), and αT-CDT: 86.46 × 89.86 × 85.91 Å^3^ (54,041 atoms), αT-TDT: 86.46 × 89.86 × 85.91 Å^3^ (54,047 atoms).

The MD processes were performed by the AMBER v18 (PMEMD) with CUDA acceleration on a 2 × NVIDIA GTX1080 Centos 7.2 × 86 computer [47]. The solvated systems were firstly minimized by 10,000 steps to remove bad contacts between water, ions and the complex systems. The equilibrium simulations with Langevin thermostats included 3 steps: 50 ps of heating and 50 ps of density equilibration with weak restraints (2 kcal/mol) on the complex followed by 500 ps of constant pressure (1 atm) equilibration at 300 K at a time step of 2 fs. The nonbonded cut-off was set to 10.0 Å and SHAKE [53] was applied for the hydrogen bonds. The production phase was then conducted with NVT resemble and conformations were collected every 10 ps. Each system was simulated for 60 ns with 3 replicates by setting different velocities of random seeds.

### 3.6. Stability Analysis

The resilience and the elasticity of the proteins and the ligands were investigated by calculating the root mean square deviation (RMSD) of the backbone atoms (Cα, N, O, C) and the heavy atoms of the docked-in ligands along the production phase simulations. The residue flexibility was checked by calculating the root mean square fluctuation (RMSF) of the backbone atoms based on each residue contribution. The trajectories were aligned by the Cα atoms of the starting structures of MD simulations beforehand to eliminate the rotation and transitions of the whole systems. The RMSD/RMSF analysis were performed by using Gromacs v 5.11 [54].

### 3.7. Local PCA

To search each stable binding pose with minimum local free energy of the binding pocket, principle component analysis (PCA) was performed firstly on the combined replicates of each production phase MD system by Gromacs v 5.11 [54]. In each system, the residues within 4.5 Å around the ligand and the heavy atoms of the ligand were both used for generating the displacement matrix of PCA. In the essential dynamic analysis (EDA), the top two PC vectors calculated from PCA were used for distinguishing the top two significant movements of the binding pocket in each system. The combined trajectories were then projected onto a subspace which was define by PC1 and PC2. We then calculated the possibility of the microstates found in the subspace for the following analysis.

### 3.8. Local Free Energy Landscape

The local free energy F(a) of each microstate a was estimated by a weighted-histogram analysis (WHAM) approach [55,56], In the WHAM approach, the local free energy of a given number of microstates that represented the configurations observed by the PC1 and 2 subspace. Thus, the free energy of a microstate a was described as:

(1) $$F\left(a\right)=-Tlog\frac{\sum_{i}n_{a}^{i}}{\sum_{j}e^{\frac{1}{T}\left(f^{j}-V_{a}^{j}\right)}}$$

where $n_{a}^{i}$ represents the frequency of the microstate a observed in trajectory i. The normalization constants $f^{j}$ are self-consistently determined as in the WHAM method [56]. We used the correction method from Marinelli et al. (2009) to describe the variation of the bias over different conformations assigned to the same cluster a [56]. $V_{a}^{i}$ is the bias potential acting on microstate a, which was estimated as the time average of the history-dependent potential acting on the trajectory i multiplied by $S_{a}$, the center of microstate a:

(2) $$V_{a}^{i}=\bar{V_{G}^{i}}\;\left(S_{a}\right)=\frac{1}{t_{sim}-t_{eq}}\int_{t_{eq}}^{t_{sim}}dt^{\prime }V_{G}^{i}\left(S_{a},t^{\prime }\right)$$

where $t_{sim}$ is the 60 ns total production simulation time of each system and $t_{eq}$ is the beginning of production phase, which is the last time frame of equilibrium when the bias potentials become stable. Therefore, according to Equations (1) and (2), we converted the possibility 2D map to the 2D local free energy landscape (LFEL) heat map and 3D LFEL surface map using Gromacs v5.11 [54] and Mathematica v11.3 [57].

### 3.9. Binding Free Energy Calculation

Since the states with lowest local free energy were found by LFEL, the nearest poses (100 frames) around that with lowest local free energy were chosen to further calculate the Gibbs binding free energies ($\Delta G_{binding}$) between receptors (TRβ1 and αT) and ligands (TDT, CDT) by performing MM/PB and GBSA [58,59] with MMPBSA.py.MPI [60]. The process can be summarized as:

$$\Delta G_{binding}=\Delta G_{com}-\left(\Delta G_{rec}+\Delta G_{lig}\right)$$

$$\Delta G_{com/rec/lig}=\Delta H-T\Delta S$$

$$\Delta H=\Delta E_{gas}+\Delta G_{sol}$$

$$\Delta E_{gas}=\Delta E_{int}+\Delta E_{vdw}+\Delta E_{ele}$$

$$\Delta G_{sol}=\Delta G_{PB/GB}+\Delta G_{NP}$$

$$\Delta G_{NP}=\gamma SASA+\beta$$

where $\Delta G_{com}$, $\Delta G_{rec}$ and $\Delta G_{lig}$ represent the free energies of the complex, the receptor and the ligand, respectively. Each of the terms ($\Delta G_{com/rec/lig}$) is equal to the discrepancy between enthalpy contribution (ΔH) and the conformational entropy (TΔS). T is the temperature of the simulated environment. ΔS is the entropy of the molecule, estimated from a normal-mode analysis of harmonic frequencies calculated at the molecular mechanics (MM) level [58]. Enthalpy (ΔH) consists of the internal energy from the gas phase ($\Delta E_{gas}$) and the solvation free energy ($\Delta G_{sol}$). $\Delta E_{gas}$ is the standard gas phase energy, which includes internal energy ($\Delta E_{int}$), van der Waals interactions ($\Delta E_{vdw}$), and electrostatic energies ($\Delta E_{ele}$). Since complex molecular dynamic simulations were only performed here, the $\Delta E_{int}$ to the binding free energy was zero. The solvation energy $\Delta G_{sol}$ is the sum of a non-polar energy ($\Delta G_{NP}$) and an electrostatic energy ($\Delta G_{PB/GB}$). was calculated from the Poisson–Boltzmann function with the default cavity radii from AMBER pomtop files. The dielectric constant was set to 1 for the interior solute and 80 for the exterior solvent. $\Delta G_{GB}$ is an alternative part for $\Delta G_{GB}$ which uses the Hawkins, Cramer, and Truhlar pairwise generalized Born model [61,62] with the parameters described by Tsui and Case [63]. The linear combinations of pairwise overlaps (LCPO) approach [64] was used to calculate the solvent accessible surface area (SASA) for the estimation of the non-polar solvation. γ (0.00542 kcal/mol × Å^2^) and β (0.92 kcal/mol) are taken from a linear regression of the solvation free energy of a set of small apolar molecules in water [65,66].

## 4. Conclusions

In this work, a library of receptors was screened against dityrosine, including CDT and TDT, on the basis of the inverse virtual screening method. The results of inverse virtual screening were sorted by energies from the highest to the lowest binding scores. Therefore, the targets with good binding affinities were identified by evaluation of the predicted binding energies. Taken as a whole, some receptors related to significant diseases, such as cancer, Parkinson′s disease, obesity, hypercholeaterolemia, diabetes, and inflammation, would be considered as possible targets for CDT/TDT, which would lay the foundation for further experimental analysis.

## Supplementary Materials

Supplementary materials can be found at http://www.mdpi.com/1422-0067/20/1/115/s1.
