# Supplementary material for: Discovery of High Affinity Receptors for Dityrosine through Inverse Virtual Screening and Docking and Molecular Dynamics
Source: Int J Mol Sci. 2018 Dec 29;20(1):115. doi: 10.3390/ijms20010115 (PMC6337580; doi:10.3390/ijms20010115)
Supplement: Supplementary file 1 [file ijms-20-00115-s001.pdf]

Article

Discovery of High Affinity Receptors for Dityrosine through Inverse Virtual Screening and Docking and Molecular Dynamics

Fangfang Wang [1,*,†], Wei Yang [2,3,†] and Xiaojun Hu [1,*]

[1] School of Life Science, Linyi University, Linyi 276000, China; huxiaojun@lyu.edu.cn
[2] Department of Microbiology, Biomedicine Discovery Institute, Monash University, Clayton, VIC 3800, Australia, zhuxiaoqing88@163.com
[3] Arieh Warshel Institute of Computational Biology, the Chinese University of Hong Kong, 2001 Longxiang Road, Longgang District, Shenzhen 518000, China
* Corresponding author: wangfangfang@lyu.edu.cn
† These authors contributed equally to this work.

Received: 09 December 2018; Accepted: 23 December 2018; Published: date

Table S1. Docking affinity scores for cis-dityrosine binding to binding proteins.

Target name	PDB/UniProtKB	Type	Affinity (kcal/mol)
Galectin-1	1A78/P56217	Lectin	-6.2±0.0
Annexin III	1AXN/P12429	Calcium/phospholipid Binding Protein	-7.5±0.0
Calmodulin	1CTR/P62158	Calcium Binding Protein	-5.8±0.0
Seminal Plasma Protein Pdc-109	1H8P/P02784	Phosphorylcholine Binding Protein	-6.6±0.0
Annexin V	1HAK/P08758	Calcium/phospholipid Binding	-7.4±0.0
Alpha 1 antitrypsin	1HP7/P01009	Protein Binding	-7.6±0.0
Histidine-Binding Protein	1HSL/P0AEU0	Binding Protein	-6.3±0.0
Intestinal Fatty Acid Binding Protein	1ICN/P02693	Binding Protein(fatty Acid)	**-9.1±0.0***
Migration Inhibitory Factor-Related Protein 14	1IRJ/P06702	Metal Binding Protein	-7.0±0.0
Lysine-, Arginine-, Ornithine-Binding Protein	1LST/P02911	Amino Acid Binding Protein	-6.5±0.0
Streptavidin	1SRJ/P22629	Biotin Binding Protein	**-8.1±0.0***
Uteroglobin	1UTR/P17559	Mammalian Pcb Binding Protein	**-8.2±0.0***
Annexin A8	1W3W/P13928	Coagulation	-7.4±0.0

Annexin A2	1W7B/P07355	Calcium Binding Protein	-6.5±0.0
Insulin-like growth factor binding protein 1	1ZT3/P08833	Peptide Binding Protein	-5.8±0.0
D-Galactose D-Glucose Binding Protein	2GBP/P0AEE5	Periplasmic Binding Protein	-6.5±0.0
Intestinal Fatty Acid Binding Protein	2IFB/P02693	Fatty Acid Binding Protein	**-8.9±0.0***

*this value was calculated with ten runs instead of one run.

Table S2. Docking affinity scores for cis-dityrosine binding to nuclear receptors.

Target name	PDB/UniProtKB	Type	Affinity (kcal/mol)
EAR-1	1A6Y/P20393	Transcription/DNA	-7.7±0.0
Cellular Retinoic-Acid-Binding Protein Type II	1CBS/P29373	Retinoic Acid Transport	**-8.0±0.0***
Nuclear Vitamin D Receptor	1DB1/P11473	Gene Regulation	**-8.3±0.0***
Lac Repressor	1EFA/P03023	Transcription/dna	**-9.1±0.0***
Epididymal Retinoic Acid-Binding Protein	1EPB/P06911	Retinoic Acid Binding Protein	-7.8±0.0
Retinoid X Receptor, Beta	1H9U/P28702	Nuclear Receptor	-7.2±0.0
Oestrogen Receptor beta	1HJ1/Q62986	Nuclear Receptor	-6.8±0.0
Vitamin D3 Receptor	1IE9/P11473	Gene Regulation	-7.9±0.0
Orphan nuclear receptor PXR	1ILH/O75469	Gene Regulation	-7.8±0.0
ERR-beta	1LO1/O95718	Hormone/growth factor receptor/DNA	-7.2±0.0
Hepatocyte nuclear factor 4-gamma	1LV2/Q14541	Transcription	-7.0±0.0
Nuclear receptor ROR-alpha	1N83/P35398	Lipid Binding Protein	**-8.2±0.0***
Thyroid hormone receptor Beta-1	1NAX/P10828	Membrane Protein	-7.4±0.0
Thyroid hormone receptor Alpha-1	1NAV/P10827	Membrane Protein	**-8.2±0.0***
Bile acid receptor	1OSH/Q96RI1	Transcription	-7.4±0.0
Bile acid receptor	1OSV/Q62735	DNA Binding Protein	-7.8±0.0
Orphan nuclear receptor NURR1	1OVL/P43354	Transcription	-6.5±0.0
Hepatocyte nuclear factor 4-alpha	1PZL/P41235	Transcription	-7.5±0.0
Estrogen Receptor beta	1QKM/Q92731	Nuclear Receptor	-6.8±0.0
Estradiol receptor	1QKT/P03372	Nuclear Receptor	-7.2±0.0
Plasma retinol-binding protein precursor	1RBP/P02753	Retinol Transport	-6.9±0.0

Transthyretin	1RLB/P02766	Complex (protein/protein)	-7.7±0.0
progesterone receptor	1SQN/P06401	Hormone/growth Factor Receptor	-7.5±0.0
Liver X receptor alpha	1UHL/P28702	DNA Binding Protein	**-8.0±0.0***
Retinoic acid receptor beta	1XAP/P10826	Transcription	-6.6±0.0
Estrogen receptor alpha	1XPC/P03372	Hormone/growth Factor Receptor	-7.8±0.0
Retinoic acid receptor RXR-alpha	1XVP/Q14994	DNA Binding Protein	**-8.4±0.0***
Orphan nuclear receptor NR5A2	1YUC/Q15466	Transcription Regulation	-7.9±0.0
Mineralocorticoid receptor	2AA2/P08235	Transcription	-7.5±0.0
Androgen receptor	2AM9/P10275	Hormone/growth Factor Receptor	-6.5±0.0
Peroxisome proliferator activated receptor delta	2AWH/Q03181	Gene Regulation	**-8.7±0.0***
Estrogen-related receptor gamma	2E2R/P62508	Transcription	-7.9±0.0
COUP transcription factor 1	2EBL/P10589	Transcription	-6.0±0.0
Peroxisome Proliferator Activated Receptor gamma	2F4B/P37231	Transcription Activator	-7.3±0.0
Retinoic acid receptor RXR-gamma	2GL8/P48443	Hormone/growth Factor Receptor	-6.1±0.0
Retinoic acid receptor gamma	2LBD/P13631	Nuclear Receptor	-7.3±0.0
Peroxisome proliferator-activated receptor alpha	2P54/Q07869	Transcription	-7.8±0.0
Orphan nuclear receptor NR4A1	2QW4/P22736	Hormone Receptor	-6.7±0.0
Orphan nuclear receptor NR1D2	2V0V/Q14995	Transcription	-6.5±0.0
Glucocorticoid receptor	3E7C/P04150	Transcription	-6.5±0.0
Estrogen Receptor alpha	3ERT/P03372	Nuclear Receptor	-7.0±0.0
Steroid hormone receptor ERR1	3K6P/P11474	Hormone Receptor	-6.5±0.0
Retinoic acid receptor RXR-alpha	3KWY/P19793	Transcription	-7.0±0.0
Nuclear receptor ROR-gamma	3L0L/P51449	Transcription	-7.8±0.0
Peroxisome proliferator-activated receptor gamma	3LMP/P37231	Transcription	-7.4±0.0

Table S3. Docking affinity scores for cis-dityrosine binding to transport proteins.

Target name	PDB/UniProtKB	Type	Affinity (kcal/mol)
Transforming protein RhoA	1A2B/P61586	Oncogene Protein	-6.6±0.0
Flavodoxin	1BU5/P00323	Electron Transport	-6.8±0.0
Cellular Retinoic-Acid-Binding Protein	1CBS/P29373	Retinoic Acid Transport	**-8.0±0.0***

Nonspecific lipid transfer protein	1CZ2/P24296	Lipid Binding Protein	**-8.1±0.0***
Retinol Binding Protein	1FEN/P18902	Transport Protein	-7.9±0.0
Clathrin Coat Assembly Protein Ap50	1HES/P84092	Endocytosis/exocytosis	-6.7±0.0
Cytochrome C2	1I8O/P00091	Electron Transport	**-8.7±0.0***
Plasma retinol-binding protein	1IIU/P41263	Transport Protein	**-8.4±0.0***
Neutrophil-Activating Protein A	1JI4/P43313	Metal Transport	-7.3±0.0
Lysine, Arginine, Ornithine-Binding Protein	1LAH/P02911	Amino Acid Transport	-6.7±0.0
Transforming protein RhoA	1OW3/Q07960	Gene Regulation/signaling Protein	-7.8±0.0
Potassium/Sodium Hyperpolarization-Activated	1Q3E/O88703	Transport Protein	-6.0±0.0
human retinol-binding protein	1QAB/P02753	Transport Protein	**-8.3±0.0***
Cystic fibrosis transmembrane regulator	1R0W/P26361	Transport Protein	-6.8±0.0
Hemoglobin	1SPG/P56251	Oxygen Transport	-7.2±0.0
Transferrin	1TFD/P19134	Iron Transport Protein	-6.9±0.0
Archaerhodopsin-1	1UAZ/P69051	Proton Transport	-6.2±0.0
Glutamate transporter	1XFH/O59010	Transport Protein	-6.7±0.0
Constitutive androstane alpha receptor	1XNX/O35627	Ligand Receptor/transcription Regulation	-7.3±0.0
Serum protein	2BX8/P02768	Transport Protein	**-8.1±0.0***
C-H-RAS P21 Protein	5P21/P01112	Oncogene Protein	-7.9±0.0

Table S4. Docking affinity scores for cis-dityrosine binding to receptors.

Target name	PDB/UniProtKB	Type	Affinity (kcal/mol)
Progesterone Receptor	1A28/P06401	Progesterone Receptor	-6.6±0.0
Tumor necrosis factor alpha	1A8M/P01375	Lymphokine	-7.5±0.0
Cytokine receptor common beta chain	1C8P/P32927	Membrane Protein	-6.1±0.0
Granulocyte Colony-stimulating Factor	1CD9/P40223	Cytokine	-6.1±0.0
Tumor Necrosis Factor Receptor Associated Protein 2	1D0A/Q12933	Apoptosis	-5.7±0.0
Elongation Factor Tu	1D2E/P49410	RNA Binding Protein	-7.0±0.0
Elongation Factor Tu	1D8T/Q7M0J8	Hydrolase/antibiotic	-6.9±0.0
Retinoid X Receptor-alpha	1DKF/P10276	Hormone/growth Factor Receptor	**-8.8±0.0***

Androgen Receptor	1E3G/P10275	Androgen Receptor	-6.7±0.0
Progesterone Receptor	1E3K/P06401	Human Progesterone Receptor	-7.5±0.0
High affinity immunoglobulin receptor alpha-subunit	1F2Q/P12319	Immune System	-6.9±0.0
Peroxisome Proliferator Activated Receptor gamma (PPAR gamma)	1FM6/P37231	Transcription	**-8.1±0.0***
Glutama receptor subunit 2	1FTK/P19491	Membrane Protein	-7.5±0.0
LIR-1	1G0X/Q8NHL6	Immune System	-7.6±0.0
Type I IL-1 receptor	1G0Y/P14778	Immune System	-7.1±0.0
androgen Receptor	1GS4/P10275	Androgen Receptor	-7.1±0.0
CD2	1HNF/P06729	T Lymphocyte Adhesion Glycoprotein	-5.9±0.0
Pyridoxine 5'-Phosphate Synthase	1HO4/P0A794	Biosynthetic Protein	-5.8±0.0
Peroxisome Proliferator Activated Receptor alpha (PPAR alpha)	1I7G/Q07869	Transcription	-6.8±0.0
Intercellular Adhesion Molecule-1	1IAM/P05362	Viral Protein Receptor	-5.9±0.0
Troponin C, Slow Skeletal and Cardiac Muscles	1IH0/P63316	Contractile Protein	-5.9±0.0
Herpesvirus entry mediator	1JMA/Q92956	Viral Protein	-6.9±0.0
Glucocorticoid-like receptor	1NHZ/P04150	Hormone Receptor	-7.4±0.0
Farnesoid X receptor	1OSH/Q96RI1	Transcription	-7.4±0.0
Farnesoid X receptor	1OSV/Q62735	DNA Binding Protein	-7.7±0.0
Metabotropic Glutamate Receptor 2	1P1N/P19491	Membrane Protein	-7.5±0.0
Glucocorticoid Receptor	1P93/P04150/Q15596	Hormone Receptor	-7.8±0.0
Estrogen Receptor	1PCG/P03372	Transcription/inhibitor	-6.6±0.0
Phosphatidylinositol 3-kinase	1PIC/P27986	Complex (phosphotransferase/receptor)	**-8.1±0.0***
Histocompatibility Leukocyte Antigen (Hla)-Cw4 (Heavy Chain)	1QQD/P30504	Immune system	-7.7±0.0
Ecdysone Receptor	1R1K/Q7SIF6	Hormone/growth Factor Receptor	-7.0±0.0
CT610	1RCW/O84616	Oxidoreductase	-6.1±0.0
Glutamate receptor-6	1S50/P42260	Membrane Protein	-7.1±0.0
Troponin C slow skeletal-cardiac muscle	1SCV/P09860	Contractile Protein	-5.9±0.0
Ionotropic glutamate receptor	1TXF/P22756	Membrane Protein	-6.4±0.0
Retinoic acid receptor RXR-beta	1UHL/Q13133	DNA Binding Protein	-7.9±0.0
Oxysterols receptor LXR-beta	1UPV/P55055	Receptor	**-8.5±0.0***
Aryl hydrocarbon receptor	1X0O/P27540	Transcription	-6.4±0.0
Constitutive androstane alpha receptor	1XNX/O35627	Ligand Receptor/transcription Regulation	**-8.1±0.0***
Peroxisome Proliferator Activated Receptor delta(PPAR delta)	1Y0S/Q03181	Hormone/growth Factor Receptor	-7.6±0.0
Glutamate receptor-5	1YCJ/P22756	Membrane Protein	-6.7±0.0
Maltose-binding periplasmic protein	1YTV/P0AEX9	Sugar Binding Protein	**-8.5±0.0***
Troponin T	1YTZ/P12620	Contractile Protein	-6.3±0.0
Troponin T fast skeletal muscle	1YV0/P12620	Contractile Protein	-6.6±0.0
HLA class I histocompatibility antigen, B-27 alpha chain	2A83/P03989	Immune System	-7.2±0.0
Glutamate receptor-2	2AIX/P19491	Membrane Protein	-6.6±0.0

IL-4 receptor	2CYK/P05112	Cytokine	-6.2±0.0
D-Galactose D-Glucose Binding Protein	2GBP/P0AEE5	Periplasmic Binding Protein	-6.6±0.0
Interleukin-10	2ILK/P22301	Cytokine	-6.6±0.0
Hexokinase B	2YHX/P04807	Transferase	-7.3±0.0

Table S5. Docking affinity scores for cis-dityrosine binding to Monoclonal Antibodies.

Target name	PDB/UniProtKB	Type	Affinity (kcal/mol)
IGG1-KAPPA 59.1 FAB	1ACY/ P01869	Complex (antibody/hiv 1 Fragment)	-7.2±0.0
Anti-dinitrophenyl-spin-label monoclonal antibody Fab fragment	1BAF	Immune System	-7.3±0.0
Mucosal Addressin Cell Adhesion Molecule-1	1BQS/Q13477	Membrane Protein	-6.6±0.0
Fab' Fragment Of Monoclonal Antibody Db3	1DBM/P01868	Immunoglobulin	**-8.4±0.0***
Anti-Tgf alpha Antibody Fab-Fragment	1E4X/P01837	Immune System	-6.8±0.0
IGG2B-KAPPA 17E8 FAB	1EAP	Catalytic Antibody	-7.7±0.0
Histocompatibility	1EEY/P01892	Immune System	-6.9±0.0
Immunoglobulin E	1F2Q/P12319	Immune System	-6.8±0.0
LIR-1	1G0X/Q8NHL6	Immune System	-6.9±0.0
Type I IL-1 receptor	1G0Y/P14778	Immune System	-7.1±0.0
Pinch protein	1G47/P48059	Cell Adhesion	-6.5±0.0
CD81 Antigen	1G8Q/P60033	Immune System	-6.1±0.0
Human Class I Histocompatibility Antigen	1HHG/P01892	Histocompatibility Antigen	**-8.4±0.0***
Human Leukocyte Antigen Class II	1I4F/P01892	Immune System	-7.1±0.0
IGG2A-KAPPA 26-10 FAB	1IGJ	Immunoglobulin	-7.9±0.0
Immunoglobulin lambda Light Chain Dimer (Mcg)	1MCJ	Immunoglobulin	**-8.9±0.0***
MHC CLASS I H-2KB HEAVY CHAIN	1OSZ/P01901	Complex (mhc I/peptide)	-7.3±0.0
CD44 antigen	1POZ/P16070	Cell Adhesion	-6.8±0.0
Integrin alpha-1/beta-1	1QCY/P56199	Cell Adhesion	-6.3±0.0
Gonadotropin alpha Subunit	1QFW/P01215	Immune System	-7.3±0.0
Histocompatibility Leukocyte Antigen (Hla)-Cw4 (Heavy Chain)	1QQD/P30504	Immune System	-7.7±0.0
MGDF receptor	1V7M/P40225	Immune System/cytokine	-7.0±0.0
Mhc Class I H-2Kb Heavy Chain	1VAC/Q7SIF6	Complex (mhc I/peptide)	-6.9±0.0
Icam-2	1ZXQ/P13598	Cell Adhesion	-5.8±0.0
Igg2B Fab Fragment	2CGR/A2P1G9	Immunoglobulin	-6.7±0.0
IGG1-KAPPA DB3 FAB	2DBL/P01868	Immunoglobulin	**-8.2±0.0***
Immunoglobulin McPC603 Fab-Phosphocholine	2MCP/P01789	Immunoglobulin	-7.1±0.0
Cell adhesion molecule	2NCM/P13595	Cell Adhesion	-6.3±0.0

murine MHC class I H-2Kb	2VAA/P01901	Complex (mhc I/peptide)	-6.9±0.0
Mhc Class I H-2Kb Heavy Chain	2VAB/P01901	Complex (mhc I/peptide)	-6.9±0.0
4-4-20 (IgG2A) Fab Fragment	4FAB/P01865	Immunoglobulin	**-8.1±0.0***

Table S6. Docking affinity scores for cis-dityrosine binding to Factor, Regulator and Hormones.

Target name	PDB/UniProtKB	Type	Affinity (kcal/mol)
Tau protein	1B5L/P56828	Cytokine	-7.3±0.0
Microtubule Protein	1CZ7/P20480	Contractile Protein	-7.7±0.0
Coagulation Factor VIII Precursor	1D7P/P00451	Blood Clotting	-6.7±0.0
Coagulation Factor VIII Precursor	1DAR/Q5SHN5	Blood Clotting	-7.2±0.0
E-selectin	1G1T/P16581	Immune System	-5.8±0.0
Hepatocyte Growth Factor Receptor	1GMN/P14210	Hormone/growth Factor	-7.0±0.0
Growth Factor Bound Protein 2	1GRI/P62993	Signal Transduction Adaptor	-6.5±0.0
Elongation Factor Tu	1HA3/Q5SHN6	Translation	**-8.1±0.0***
Heparin-Binding Growth Factor 1	1HKN/P05230	Growth Factor	-5.9±0.0
Heat Shock 70 kDa protein 8	1HX1/P19120	Chaperone/chaperone Inhibitor	**-8.5±0.0***
Elongation Factor 1-alpha	1IJE/P02994	Translation	-7.6±0.0
Migration Inhibitory Factor-Related Protein 14	1IRJ/P06702	Metal Binding Protein	-6.8±0.0
Neutrophil-Activating Protein A	1JI4/P43313	Metal Transport	-6.9±0.0
Neurophysin 2	1JK4/P01180	Neuropeptide	-6.6±0.0
Imaginal Disc Growth Factor-2	1JND/Q9V3D4	Hormone/growth Factor	-7.6±0.0
Elongation Factor 1-alpha	1JNY/P35021	Translation	-7.3±0.0
Nuclear Orphan Receptor Lxr-beta	1K4W/P45446	Hormone/growth Factor	**-8.4±0.0***
Bone Morphogenetic Protein-7	1M4U/P18075	Hormone/growth Factor	-6.3±0.0
Glucocorticoid-like receptor	1NHZ/P04150	Hormone Receptor	-7.4±0.0
Glucocorticoid Receptor	1P93/P04150	Hormone Receptor	-7.0±0.0
Insulin	1Q4V/P01308	Hormone/growth Factor	-6.5±0.0
Gonadotropin alpha subunit	1QFW/P01215	Immune System	**-8.6±0.0***
FGF-4 receptor	1RML/P05230	Growth Factor	-6.4±0.0
Elongation Factor 1-alpha	1TUI/Q01698	Elongation Factor	-6.3±0.0
Insulin	1TYL/P01308	Hormone	-6.5±0.0
VEGF receptor	1VPP/P15692	Growth Factor/growth Factor Inhibitor	-6.0±0.0
Chaperonin	1VQ0/Q9X1B4	Chaperone	-6.4±0.0

deamino-oxytocin	1XY1/P01175	Hormone	-5.0±0.0
IGF binding protein-1	1ZT3/P08833	Peptide Binding Protein	-5.8±0.0
Heat shock protein 16.3	2BYU/Q41560	Chaperone	-7.2±0.0
Kinesin-like protein KIF11	2FKY/P52732	Cell Cycle	-7.5±0.0
Methylparaben Insulin	3MTH/P01315	Hormone	-6.3±0.0
Concanavalin A	5CNA/P02866	Lectin(agglutinin)	-6.6±0.0
Galectin-7	5GAL/P47929	Lectin	-6.3±0.0

Table S7. Docking affinity scores for cis-dityrosine binding to Structural Proteins.

Target name	PDB/UniProtKB	Type	Affinity (kcal/mol)
Yeast Hypothetical Protein	1CT5/P38197	Structural Genomics	-6.0±0.0
Ribosomal Protein S6 Kinase	1DMG/P38516	Gene Regulation	-6.3±0.0
Cardiac Troponin C	1DTL/P09860	Structural Protein	-7.9±0.0
ATP binding cassette	1F3O/Q58206	Structural Genomics	**-8.0±0.0***
HIV gp41 protein	1FAV/P03069	Viral Protein	-6.4±0.0
Pinch protein	1G47/P48059	Cell Adhesion	-6.3±0.0
Troponin C, Slow skeletal and cardiac muscles	1IH0/P63316	Contractile Protein	-6.2±0.0
Tubulin alpha chain	1JFF/P02550	Structural Protein	**-8.1±0.0***
CD44 antigen	1POZ/P16070	Cell Adhesion	-6.6±0.0
Integrin alpha-1/beta-1	1QCY/P56199	Cell Adhesion	-6.6±0.0
GP41 Envelope Protein	1QR8/Q76270	Viral Protein	-6.0±0.0
Troponin C, slow skeletal-cardiac muscle	1SCV/P09860	Contractile Protein	-6.3±0.0
Nucleotidyltransferase	1WOT/P83814	Structural Genomics	-6.4±0.0
Troponin T, fast skeletal muscle	1YV0/P12620	Contractile Protein	-6.8±0.0
Icam-2	1ZXQ/P13598	Cell Adhesion	-5.8±0.0
Myosin light chain kinase family	2BKH/Q29122	Motor Protein/metal Binding Protein	-7.9±0.0
Neural cell adhesion molecule	2NCM/P13595	Cell Adhesion	-6.2±0.0

Table S8. Docking affinity scores for cis-dityrosine binding to Signaling Proteins.

Target name	PDB/UniProtKB	Type	Affinity (kcal/mol)
Rho	1DS6/P52566	Signaling Protein	-7.8±0.0

Neutrophil Cytosol Factor 2	1E96/P19878	Signaling Protein	**-7.5±0.0**
Metabotropic Glutamate Receptor Subtype 1	1EWK/P23385	Signaling Protein	-6.9±0.0
Soluble tumor necrosis factor receptor 1	1FT4/P19438	Signaling Protein	-6.3±0.0
Rhodopsin	1HZX/P02699	Signaling Protein	-7.2±0.0
Na+ H+ ion exchange	1I92/O14745	Signaling Protein	-6.4±0.0
Metabotropic Glutamate Receptor Subtype 1	1ISS/P23385	Signaling Protein	-7.7±0.0
Ran-GPPNHP-RanBP1-RanGAP	1K5D/P62826	Signaling Protein	**-9.0±0.0***
Transforming protein RhoA	1KMQ/P61586	Signaling Protein	-7.3±0.0
Voltage-gated calcium channel beta2a subunit	1T0J/Q8VGC3	Signaling Protein	-7.4±0.0
Sonic hedgehog	1VHH/Q62226	Signaling Protein	-6.7±0.0
Signal Transducer and Activator Of Transcription 1-alpha/beta	1YVL/P42224	Signaling Protein	-6.5±0.0
Regulator of G-protein signaling 17	1ZV4/Q9UGC6	Signaling Protein	-6.1±0.0

Table S9. Docking affinity scores for cis-dityrosine binding to Ion Channels.

Target name	PDB/UniProtKB	Type	Affinity (kcal/mol)
Hypothetical Protein Slr1257	1II5/P73797	Membrane Protein	-7.9±0.0
Voltage-Gated Potassium Channel	1JVM/P0A334	Membrane Protein	**-8.6±0.0***
Chloride intracellular channel protein 4	2AHE/Q9Y696	Metal Transport	-6.5±0.0

Table S10. Docking affinity scores for cis-dityrosine binding to RNA.

Target name	PDB/UniProtKB	Type	Affinity (kcal/mol)
Elongation Factor TU	1D2E/P49410	RNA Binding Protein	-7.0±0.0
Elongation Factor TU	1D8T/P0CE47	Hydrolase/antibiotic	-6.9±0.0

Table S11. Docking affinity scores for cis-dityrosine binding to Lipid Binding Protein.

Target name	PDB/UniProtKB	Type	Affinity (kcal/mol)
Adipocyte Lipid-Binding Protein	1LIC/P04117	Lipid Binding Protein	-7.6±0.0
KES1 protein	1ZHY/P35844	Lipid Binding Protein	**-8.6±0.0***

Table S12. Docking affinity scores for cis-dityrosine binding to other undefined receptors.

Target name	PDB/UniProtKB	Type	Affinity (kcal/mol)
C-reactive protein	1B09/P02741	Immune System	-7.7±0.0
bilin binding protein (BBP)	1BBP/P09464	Bilin Binding	**-8.4±0.0***
Congerin I	1C1L/P26788	Sugar Binding Protein	-7.4±0.0
C-H-Ras P21 Protein	1CRP/P01112	Oncogene Protein	-7.4±0.0
GLGF-Domain Protein Homer	1DDV/Q9Z214	Signaling Protein	-6.5±0.0
Human Neutrophil Gelatinase	1DFV/P80188	Sugar Binding Protein	-7.0±0.0
Cholera Toxin B	1EEI/Q57193	Toxin	-7.6±0.0
Transthyretin	1ETA/P02766	Transport(thyroxine)	-5.9±0.0
Congerin II	1IS3/Q9YIC2	Sugar Binding Protein	-6.2±0.0
Catabolite Gene Activator Protein	1J59/P0ACJ8	Gene Regulation/dna	-7.7±0.0
Galectin-3	1KJL/P17931	Sugar Binding Protein	-5.7±0.0
Major Urinary Protein	1MUP/P02762	Pheromone Binding	-5.7±0.0
Holo-Neocarzinostatin	1NCO/P0A3R9	Antibacterial and Antitumor Protein	-7.1±0.0
Actin, alpha skeletal muscle	1NWK/P68135	Contractile Protein	**-8.9±0.0***
ROP Protein	1ROP/P03051	Transcription Regulation	-5.4±0.0
Galectin-2	1ULE/Q9P4R8	Sugar Binding Protein	-6.2±0.0
STAT Protein	1UUS/O00910	Signal Transduction	-7.6±0.0
Trp RNA-Binding Attenuation Protein	1WAP/P19466	RNA Binding Attenuation Protein	-6.3±0.0
Galectin	1WW7/Q6WY08	Sugar Binding Protein	-6.0±0.0

Table S13. Docking affinity scores for cis-dityrosine binding to other Enzymes.

Target name	PDB/UniProtKB	Therapeutic area	Affinity (kcal/mol)
Calcium/calmodulin-dependent protein kinase	1A06/Q63450	Kinase	-6.9±0.0
Protein Kinase C, beta Type	1A25/P68403	Calcium Binding Protein	-6.6±0.0
Branched-Chain Amino Acid Aminotransferase	1A3G/P0AB80	Aminotransferase	-6.7±0.0
Adenosine Deaminase	1A4M/P03958	Hydrolase	-7.7±0.0
Alpha-thrombin	1A4W/P00734	Hydrolase/hydrolase Inhibitor	**-8.5±0.0***

Plasminogen Activator Inhibitor Type 1	1A7C/P05121	Hydrolase Inhibitor/peptide	**-8.1±0.0***
Protein Disulfide Oxidoreductase	1A8L/Q51760	Oxidoreductase	-6.4±0.0
Retroviral protease	1A94/P03367	Hydrolase/hydrolase Inhibitor	-7.6±0.0
HIV Protease	1AAQ/P03367	Hydrolase/hydrolase Inhibitor	-7.4±0.0
DNA gyrase	1AB4/P0AES4	Topoisomerase	-7.0±0.0
Abl tyrosine kinase	1ABO/P55194	Complex (kinase/peptide)	-6.1±0.0
Cytochrome Peroxidase	1AC4/P00431	Oxidoreductase	-7.8±0.0
Cytochrome Peroxidase	1AC8/P00431	Oxidoreductase	**-8.2±0.0***
Acetylcholinesterase	1ACJ/P04058	Hydrolase(carboxylic Esterase)	-7.5±0.0
Aspartate Carbamoyltransferase	1ACM/P0A786	Transferase	-7.6±0.0
Aconitate Hydratase	1ACO/P20004	Lyase(carbon Oxygen)	-7.9±0.0
Alcohol Dehydrogenase	1ADB/P00327	Oxidoreductase (nad(a) Choh(d))	-7.7±0.0
Alcohol Dehydrogenase	1ADC/P00327	Oxidoreductase (nad(a) Choh(d))	**-8.0±0.0***
Adenosine Deaminase	1ADD/P03958	Hydrolase(acting in Cyclicamidines)	-7.8±0.0
Alcohol Dehydrogenase	1ADF/P00327	Oxidoreductase(nad(a) Choh(d))	-7.3±0.0
Serine Proteinase alpha-thrombin	1AE8/P00734	Hydrolase/hydrolase Inhibitor	**-8.1±0.0***
Cytochrome C Peroxidase	1AEB/P00431	Oxidoreductase	-7.9±0.0
Actinidin	1AEC/P00785	Hydrolase	-7.0±0.0
Cytochrome C Peroxidase	1AEV/P00431	Oxidoreductase	-7.9±0.0
Aldehyde dehydrogenase	1AG8/P20000	Oxidoreductase	-7.8±0.0
Serine protease	1AH2/P27693	Serine Protease	-6.7±0.0
Phospholipase C	1AH7/P09598	Hydrolase	-7.8±0.0
Alpha-Momorcharin	1AHA/P16094	Glycosidase	-6.9±0.0
HIV-1 Protease	1AJV/P03366	Aspartyl Protease	-7.8±0.0
HIV-1 Protease	1AJX/P03366	Aspartyl Protease	-7.7±0.0
Glycolate Oxidase	1AL8/P05414	Flavoprotein	-6.9±0.0
Pepsin	1AM5/P56272	Aspartyl Protease	-7.6±0.0
Angiogenin	1ANG/P03950	Hydrolase (vascularization)	-6.1±0.0
cAMP-dependent Protein Kinase	1APM/P05132	Transferase(phosphotransferase)	-7.1±0.0
Penicillopepsin	1APV/P00798	Hydrolase/hydrolase Inhibitor	-7.7±0.0
Aspartate Aminotransferase	1ASE/P00509	Aminotransferase	-7.5±0.0

Atrolysin C	1ATL/P15167	Hydrolase/hydrolase Inhibitor	-6.9±0.0
Carboxylesterase	1AUR/Q53547	Hydrolase	-7.2±0.0
Carbonic Anhydrase II	1AVN/P00918	Lyase	-6.8±0.0
Human Fibroblast Collagenase	1AYK/P03956	Metalloprotease	-7.1±0.0
Phosphoenolpyruvate carboxykinase	1AYL/P22259	Kinase (transphosphorylating)	-7.3±0.0
Carbonic Anhydrase I	1AZM/P00915	Lyase(oxo Acid)	-7.3±0.0
Signal peptidase-I	1B12/P00803	Hydrolase	-6.8±0.0
TGF-beta receptor type I	1B6C/P36897	Complex (isomerase/protein Kinase)	**-8.6±0.0***
Haloalkane Dehalogenase	1B6G/P22643	Hydrolase	-6.4±0.0
Glutamate racemase	1B74/P56868	Isomerase	-7.8±0.0
malate dehydrogenase	1B8P/Q9ZF99	Oxidoreductase	-7.1±0.0
Glutamate Dehydrogenase	1BGV/P24295	Oxidoreductase	-7.5±0.0
17-beta Hydroxysteroid-Dehydrogenase Type 1,Estradiol 17 beta dehydrogenase	1BHS/P14061	Oxidoreductase	-7.9±0.0
Thymidylate Synthetase	1BID/P0A884	Methyltransferase	-7.5±0.0
Fructose biphosphatase	1BIF/P25114	Bifunctional Enzyme	**-9.4±0.0***
Nadph-Flavin Oxidoreductase	1BKJ/Q56691	Oxidoreductase	-7.6±0.0
beta-Lactamase	1BLH/P00807	Hydrolase(beta Lactamase)	-7.4±0.0
Chymotrypsin-like elastase family member 1	1BMA/P00772	Hydrolase/hydrolase Inhibitor	-7.2±0.0
Map Kinase P38	1BMK/Q16539	Transferase	-7.5±0.0
Serine Proteinase alpha-thrombin	1BMN/P00734	Hydrolase/hydrolase Inhibitor	**-8.1±0.0***
Interleukin-beta convertase	1BMQ/P29466	Hydrolase	-6.9±0.0
Methionine Synthase	1BMT/P13009	Methyltransferase	-7.5±0.0
Histone acetyltransferase	1BOB/Q12341	Acetyltransferase	-7.9±0.0
Cytosine DNA methyltransferase	1BOO/P11409	Transferase	-7.8±0.0
Dihydrofolate Reductase	1BOZ/P00374	Oxidoreductase	**-8.3±0.0***
Trypsin	1BRA/P00763	Proteinase/inhibitor	-6.9±0.0
Activin type-II receptor	1BTE/P27038	Transferase	-6.3±0.0
Liver Alcohol Dehydrogenase	1BTO/P00327	Oxidoreductase	**-8.2±0.0***
Delta 5-3-ketosteroid isomerase	1BUQ/P00947	Isomerase	-7.2±0.0
Protein (Cytochrome P450 BM-3)	1BVY/P14779	Oxidoreductase	**-8.6±0.0***

NADPH Dehydrogenase 1	1BWK/Q02899	Oxidoreductase	-7.2±0.0
Beta-Amylase	1BYB/P10538	Hydrolase(o Glycosyl)	**-8.2±0.0***
Dihydrofolate Reductase	1BZF/P00381	Oxidoreductase	**-8.1±0.0***
Carbonic Anhydrase I	1BZM/P00915	Lyase(oxo Acid)	-6.7±0.0
Neutrophil Collagenase	1BZS/P22894	Hydrolase	**-8.5±0.0***
Csdb Protein	1C0N/P77444	Lyase	-7.0±0.0
Ras-Binding Domain of the Serine/Threonine Kinase C-Raf1	1C1Y/P62834	Signaling Protein	**-8.3±0.0***
Glycinamide Ribonucleotide Formyltransferase	1C2T/P08179	Transferase	-7.5±0.0
Lumazine Synthase	1C41/Q9UVT8	Transferase	**-9.6±0.0***
Ornithine Decarboxylase	1C4K/P43099	Lyase	**-8.0±0.0***
Aspartate Aminotransferase, Cytoplasmic	1C9C/P00509	Transferase	-7.3±0.0
Fkbp-rapamycin Associated Protein	1C9H/P68106	Immune System	-6.4±0.0
Glutamate Mutase	1CB7/P80078	Isomerase	**-8.3±0.0***
Cobalt-Precorrin-4 Transmethylase	1CBF/O87696	Methyltransferase	**-8.4±0.0***
Carboxypeptidase A	1CBX/P00730	Hydrolase(c Terminal Peptidase)	-6.7±0.0
Arginase	1CEV/P53608	Hydrolase	-7.3±0.0
Cathepsin G	1CGH/P08311	Hydrolase/hydrolase Inhibitor	-7.1±0.0
n/a	1CGZ/P30074	Transferase	-6.9±0.0
Pnp Oxidase	1CI0/P38075	Oxidoreductase	**-8.2±0.0***
Protein (Triosephosphate Isomerase)	1CI1/P52270	Triosephosphate Isomerase	-6.6±0.0
Thymidylate Synthase	1CI7/P13100	Transferase	**-8.2±0.0***
Carbonic Anhydrase II	1CIL/P00918	Lyase(oxo Acid)	-7.7±0.0
Carbonic Anhydrase II	1CIM/P00918	Lyase(oxo Acid)	-6.8±0.0
Carbonic Anhydrase II	1CIN/P00918	Lyase(oxo Acid)	-7.0±0.0
Serine Hydroxymethyltransferase, Mitochondrial	1CJ0/P07511	Transferase	-7.3±0.0
MRNA Capping Enzyme	1CKN/Q84424	Capping Enzyme	-7.0±0.0
Cholesterol Esterase	1CLE/P32947	Lipase	-7.1±0.0
Nitrate reductase	1CNF/P17571	Oxidoreductase	-7.5±0.0
Carbonic Anhydrase II	1CNW/P00918	Lyase (oxo Acid)	-7.0±0.0
Carbonic Anhydrase II	1CNX/P00918	Lyase (oxo Acid)	-6.8±0.0
Carbonic AnhydraseII	1CNY/P00918	Lyase (oxo Acid)	-7.0±0.0

Cholesterol Oxidase	1COY/P22637	Oxidoreductase(oxygen Receptor)	**-9.0±0.0***
Aminopeptidase	1CP7/P80561	Hydrolase	-6.3±0.0
Carboxypeptidase A	1CPS/P00730	Hydrolase(c Terminal Peptidase)	-6.9±0.0
Cytochrome P450-Terp	1CPT/P33006	Oxidoreductase(oxygenase)	**-8.5±0.0***
Epoxide hydrolase	1CQZ/P34914	Hydrolase	**-8.4±0.0***
DNA Primase/Helicase	1CR1/P03692	Transferase	-7.4±0.0
Basic-type creatine kinase	1CRK/P11009	Transferase	-6.7±0.0
Cathepsin B	1CSB/P07858	Hydrolase/hydrolase Inhibitor	-7.1±0.0
Casein kinase I	1CSN/P40233	Phosphotransferase	**-8.1±0.0***
Cytidine Deaminase	1CTT/P0ABF6	Hydrolase	-7.3±0.0
Prostatic acid phosphatase	1CVI/P15309	Hydrolase	-7.7±0.0
Gingipain R	1CVR/P95493	Hydrolase/hydrolase Inhibitor	-6.1±0.0
Coagulation Factor VIIa	1CVW/P08709	Hydrolase/hydrolase Inhibitor	-7.3±0.0
Tryptophan Synthase	1CW2/P00929	Lyase	-7.4±0.0
Cyclooxygenase-2	1CX2/Q05769	Oxidoreductase	**-8.8±0.0***
DNA Topoisomerase I	1CY1/P06612	Isomerase	-7.1±0.0
DNA Topoisomerase I	1CY9/P06612	Isomerase	-6.1±0.0
Cyclophilin Receptor	1CYN/P23284	Isomerase/immunosuppressant	-6.8±0.0
Microtubule Motor Protein NCD	1CZ7/P20480	Contractile Protein	-7.7±0.0
Alcohol Dehydrogenase	1D1T/P40394	Oxidoreductase	-7.9±0.0
Maly Protein	1D2F/P23256	Transferase	-6.5±0.0
Acid phosphatase	1D2T/Q9S1A6	Hydrolase	-7.4±0.0
Myeloperoxidase	1D2V/P05164	Oxidoreductase	**-8.0±0.0***
Dihydroorotate Dehydrogenase, Mitochondrial	1D3G/Q02127	Oxidoreductase	**-9.7±0.0***
Serine Proteinase alpha-thrombin	1D4P/P00734	Hydrolase/hydrolase Inhibitor	-7.6±0.0
Pokeweed Antiviral Protein	1D6A/P10297	Hydrolase	-7.5±0.0
Topoisomerase III	1D6M/P14294	Isomerase	-7.5±0.0
Hypoxanthine-Guanine Phosphoribosyltransferase	1D6N/P00492	Transferase	**-8.7±0.0***
O-acetylserine Sulfhydrylase	1D6S/P0A1E3	Lyase	-6.6±0.0
Copper Amine Oxidase	1D6U/P46883	Oxidoreductase	-7.4±0.0
Coagulation Factor VIIa	1DAN/P08709	Hydrolase/hydrolase Inhibitor	-7.6±0.0

Cytosolic Phospholipase A2	1DB4/P14555	Hydrolase/hydrolase Inhibitor	-7.3±0.0
Deacetoxycephalosporin C Synthase	1DCS/P18548	Oxidoreductase	-6.9±0.0
Dihydrofolate Reductase	1DF7/P9WNX1	Oxidoreductase	-7.2±0.0
Peptide deformylase	1DFF/P0A6K3	Hydrolase	-7.9±0.0
Dialkylglycine decarboxylase	1DGD/P16932	Lyase	-7.1±0.0
Adenosine kinase	1DGM/Q9TVW2	Transferase	-7.6±0.0
Dihydrofolate Reductase	1DHF/P00374	Oxido Reductase	**-8.0±0.0***
Dihydrofolate Reductase	1DHJ/P0ABQ4	Oxidoreductase	**-8.5±0.0***
Estrogenic 17-Beta Hydroxysteroid Dehydrogenase	1DHT/P14061	Oxidoreductase	**-8.1±0.0***
Cyclin-Dependent Kinase 2 (Cdk2)	1DI8/P24941	Transferase	**-8.4±0.0***
Methylenetetrahydrofolate Reductase	1DIA/P11586	Oxidoreductase	-7.9±0.0
D-Xylose Isomerase	1DID/P12070	Isomerase(intramolecular Oxidoreductase)	**-8.1±0.0***
HIV-1 Protease	1DIF/P03367	Aspartic Proteinase	-7.6±0.0
Microbial Dihydrofolate Reductase	1DIH/P04036	Oxidoreductase	-7.2±0.0
Dihydrofolate Reductase	1DIS/P00381	Oxido Reductase	**-9.1±0.0***
Prostaglandin H2 Synthase-1	1DIY/P05979	Oxidoreductase	-7.5±0.0
8-Amino-7-Oxonanoate Synthase	1DJE/P12998	Transferase	-7.9±0.0
Ribosomal Protein L4	1DMG/P38516	Gene Regulation	-6.1±0.0
Catechol 1,2-Dioxygenase	1DMH/P07773	Oxidoreductase	-7.5±0.0
Phenylalanine Hydroxylase	1DMW/P00439	Oxidoreductase	**-8.2±0.0***
Atrial Natriuretic Peptide Receptor A	1DP4/P18910	Hormone/growth Factor Receptor	-7.7±0.0
Protein (Antigen 85-C)	1DQY/P9WQN9	Immune System	-6.9±0.0
Dihydrofolate Reductase	1DR1/P00378	Oxidoreductase	**-8.3±0.0***
Dihydrofolate Reductase	1DRF/P00374	Oxidoreductase (ch Nh(d) NAD or Nadp(a))	-7.9±0.0
Protein Kinase C, Alpha type	1DSY/P05696	Transferase	-7.8±0.0
Biotin Carboxylase,Acetyl CoA carboxylase	1DV1/P24182	Ligase	-7.4±0.0
Serine Proteinase alpha-thrombin	1DWB/P00734	Hydrolase/hydrolase Inhibitor	-7.6±0.0
Serine Proteinase alpha-thrombin	1DWC/P00734	Hydrolase/hydrolase Inhibitor	**-8.0±0.0***
Serine Proteinase alpha-thrombin	1DWD/P00734	Hydrolase/hydrolase Inhibitor	-7.9±0.0
Class II Chitinase	1DXJ/O81934	Hydrolase	-7.1±0.0

Nonstructural Protein NS2,Hepatitis C virus NS3 protease	1DXP/P26662	Hydrolase	-6.2±0.0
Nonstructural Protein NS3,Hepatitis C virus NS3 helicase	1DY9/P26662	Hydrolase/hydrolase Inhibitor	-6.6±0.0
beta-Glucosidase	1E1F/P49235	Hydrolase	**-9.7±0.0***
Thymidine Kinase	1E2K/P03176	Transferase	-6.4±0.0
Steroid Delta-Isomerase	1E3V/P07445	Isomerase	-6.8±0.0
Delta-Aminolevulinic Acid Dehydratase	1E51/P13716	Lyase	-6.4±0.0
Methionine gamma-lyase	1E5F/O15564	Lyase	-6.8±0.0
GDP fucose synthetase	1E6U/P32055	Epimerase/reductase	**-8.1±0.0***
GDP-Fucose Synthetase	1E7S/P32055	Epimerase/reductase	**-8.3±0.0***
Pteridine Reductase	1E7W/Q01782	Oxidoreductase	**-8.4±0.0***
Cytochrome C	1E86/P00138	Electron Transport	-6.4±0.0
Enolase	1EBG/P00924	Carbon Oxygen Lyase	-7.0±0.0
Escherichia-Coli Chorismate Mutase	1ECM/P0A9J8	Chorismate Mutase	-6.3±0.0
Nitric Oxide Synthase	1ED5/P29473	Oxidoreductase	**-8.3±0.0***
Endothiapepsin	1EED/P11838	Hydrolase/hydrolase Inhibitor	-7.8±0.0
HIV-1 Reverse Transcriptase	1EET/P03366	Viral Protein	**-8.9±0.0***
Hydroxysteroid Sulfotransferase	1EFH/Q06520	Transferase	-7.9±0.0
Bovine Mitochondrial F1-ATPase	1EFR/P19483/P00829/ P05631	Hydrolase/antibiotic	**-9.6±0.0***
Modification Methylase Rsri	1EG2/P14751	Transferase	-7.7±0.0
DNA Gyrase B Subunit	1EI1/P0AES6	Isomerase	-7.5±0.0
Lumazine Synthase	1EJB/P50861	Transferase	-7.9±0.0
Branched Chain Amino Acid-dependent Aminotransferase	1EKV/O15382	Transferase	-7.3±0.0
Elastase	1ELA/P00772	Hydrolase/hydrolase Inhibitor	-7.3±0.0
Elastase	1ELC/P00772	Hydrolase/hydrolase Inhibitor	-7.5±0.0
Porcine Pancreatic Elastase	1ELF/P00772	Complex (hydrolase/inhibitor)	-7.3±0.0
Endothiapepsin	1EPP/P11838	Hydrolase/hydrolase Inhibitor	-7.7±0.0
Prostaglandin H2 Synthase-1, COX-1	1EQH/P05979	Oxidoreductase	-7.8±0.0
Thio oxidoreductase	1ERU/P10599	Oxidoreductase	-5.5±0.0
BONE MORPHOGENETIC PROTEIN-2(bmp2)	1ES7/P12643	Cytokine	-7.1±0.0
Epsilon- Thrombin	1ETR/P00735	Hydrolase/hydrolase Inhibitor	-7.0±0.0

Epsilon- Thrombin	1ETS/P00735	Hydrolase/hydrolase Inhibitor	-7.8±0.0
Epsilon- Thrombin	1ETT/P00735	Hydrolase/hydrolase Inhibitor	-7.8±0.0
D-Amino acid oxidase	1EVI/P00371	Oxidoreductase	**-8.6±0.0***
Glycerol-3-phosphate dehydrogenase	1EVZ/P90551	Oxidoreductase	**-8.0±0.0***
Phospholipase D	1F0I/P84147	Hydrolase	-6.9±0.0
Cruzain	1F2A/P25779	Hydrolase	-6.6±0.0
PAK-1 protein kinase	1F3M/Q13153	Transferase	-7.0±0.0
Carboxypeptidase A	1F57/P00730	Hydrolase	-6.8±0.0
A-G adenine DNA glycAosylase	1F6O/P29372	Hydrolase/dna	-6.7±0.0
Lipoxygenas-1	1F8N/P08170	Oxidoreductase	**-8.0±0.0***
Coagulation Factor Xa	1FAX/P00742	Coagulation Factor	-7.6±0.0
Fibroblast Growth Factor Receptor 1	1FGK/P11362	Phosphotransferase	-6.7±0.0
Oxalate oxidase	1FI2/P45850	Oxidoreductase	-6.3±0.0
Factor XIII	1FIE/P00488	Transferase	-7.6±0.0
Beta-Acrosin	1FIW/Q9GL10	Hydrolase	-6.8±0.0
Carboxylase	1FIY/P00864	Complex (lyase/inhibitor)	**-9.2±0.0***
3-Alpha hydroxysteroid dehydrogenase	1FJH/P80702	Oxidoreductase	-7.5±0.0
Fk506 Binding Protein (Fkbp)	1FKF/P62942	Isomerase	-6.7±0.0
Fk506 Binding Protein (Fkbp)	1FKG/P62942	Cis Trans Isomerase	-6.7±0.0
Fk506 Binding Protein (Fkbp)	1FKI/P62942	Cis Trans Isomerase	-6.8±0.0
Memapsin 2 (Beta-Secretase)	1FKN/P56817	Hydrolase/hydrolase Inhibitor	-7.8±0.0
Peptidase T	1FNO/P26311	Hydrolase	**-8.0±0.0***
Xanthine dehydrogenase	1FO4/P80457	Oxidoreductase	-7.2±0.0
Fructose-1,6-bisphosphatase	1FRP/P00636	Hydrolase(phosphoric Monoester)	-6.8±0.0
Protein farnesyl transferase	1FT2/Q04631	Transferase	**-8.4±0.0***
Methionine sulfoxide reductase	1FVG/P54149	Oxidoreductase	-6.3±0.0
Adenylate cyclase	1FX2/Q99279	Lyase	-6.8±0.0
Glucose phosphate thymidylyltransferase	1FXO/Q9HU22	Transferase	**-8.9±0.0***
Phosphotransferase	1FYN/P06241	Transferase	-5.4±0.0
Metalloendopeptidase	1G12/P81054	Hydrolase	-7.1±0.0
Glucosidase-471	1GAI/ P69327	Hydrolase	-7.5±0.0

Macrophage Migration Inhibitory Factor	1GCZ/P14174	Immune System	-6.8±0.0
Aspartate Aminotransferase	1GD9/O59096	Transferase	-6.9±0.0
Protein Tyrosine Phosphatase 1b	1GFY/P18031	Hydrolase	-6.3±0.0
Gamma Chymotrypsin	1GHB/P00766	Hydrolase/hydrolase Inhibitor	-6.9±0.0
Cell Division Protein Kinase 2(CDK2)	1GIH/P24941	Transferase	**-8.4±0.0***
Cyclin Dependent Kinase 2(Cdk2)	1GII/P24941	Transferase	**-8.1±0.0***
Aspartate-semialdehyde Dehydrogenase	1GL3/P0A9Q9	Oxidoreductase	-7.4±0.0
Tec tyrosine kinase	1GL5/P24604	Transferase	-6.1±0.0
Glutathione S-transferase	1GLP/P19157	Transferase(glutathione)	-7.0±0.0
Glutathione S-transferase	1GLQ/P19157	Transferase(glutathione)	-6.2±0.0
Monoamine Oxidase	1GOS/P27338	Oxidoreductase	**-9.3±0.0***
Spinach glycolate oxidase	1GOX/P05414	Oxidoreductase (oxygen(a))	-7.3±0.0
Leucoanthocyanidin Dioxygenase	1GP6/Q96323	Oxidoreductase	-7.4±0.0
Glycogen Phosphorylase B	1GPB/P00489	Glycogen Phosphorylase	**-8.9±0.0***
Acetylcholinesterase	1GPK/P04058	Hydrolase	**-8.4±0.0***
GMP synthetase	1GPM/P04079	Transferase (glutamine Amidotransferase)	**-8.3±0.0***
Acetylcholinesterase	1GPN/P04058	Hydrolase	-7.8±0.0
Transketolase 1	1GPU/P23254	Transferase	-7.7±0.0
Acetylcholinesterase	1GQS/P04058	Hydrolase	**-8.0±0.0***
Glutathione reductase	1GRE/P00390	Oxidoreductase	**-8.0±0.0***
Glutathione Transferase A1-1	1GSF/P08263	Transferase (glutathione)	**-8.7±0.0***
Thymidine Kinase	1GSI/P9WKE1	Transferase	-6.9±0.0
Glutathione S-transferase	1GTB/P08515	Glutathione Transferase	-6.2±0.0
Cyclin Dependent Kinase 2(Cdk2)	1GZ8/P24941	Transferase	
Leukocyte Elastase	1H1B/P08246	Hydrolase	-6.2±0.0
Nitrogenase Molybdenum Iron Protein Alpha Chain	1H1L/P00466	Oxidoreductase	**-8.3±0.0***
CDK2	1H28/P24941	Cell Cycle/transferase Substrate	**-8.0±0.0***
Sulfurtransferase	1H4K/P52197	Transferase	-6.5±0.0
Glycogen phosphorylase	1H5U/P00489	Glycogen Metabolism	-7.1±0.0
Dihydropyrimidine dehydrogenase	1H7X/Q28943	Electron Transfer	**-8.0±0.0***
Polyamine oxidase	1H82/O64411	Oxidoreductase	**-9.3±0.0***

Serine Proteinase alpha-thrombin	1H8D/P00734	Hydrolase/hydrolase Inhibitor	-7.4±0.0
Proto-Oncogene Tyrosine-Protein Kinase LCK	1H92/P06239	Transferase	-6.1±0.0
Phosphatidylinositol 3-kinase	1H9O/P27986	Transferase/receptor	-5.8±0.0
Elongation Factor Tu	1HA3/Q5SHN6	Translation	**-8.2±0.0***
Isopenicillin N Synthase	1HB2/P05326	Antibiotic Biosynthesis	**-8.7±0.0***
HIV-1 Protease	1HBV/P03366	Hydrolase (acid Protease)	-7.5±0.0
3-alpha-hydroxysteroid Dehydrogenase	1HDC/P19992	Oxidoreductase	-7.8±0.0
Galectin-10	1HDK/Q05315	Serine Esterase	-6.2±0.0
Biliverdin Ix Beta Reductase	1HDO/P30043	Biliverdin Ix Beta Reductase	-7.7±0.0
Serine Proteinase alpha-thrombin	1HDT/P00734	Hydrolase/hydrolase Inhibitor	-7.5±0.0
Alcohol Dehydrogenase	1HDY/P00325	Oxidoreductase(nad(a) Choh(d))	-7.8±0.0
HIV-1 Protease	1HEF/P03366	Hydrolase/hydrolase Inhibitor	-6.2±0.0
Fibroblast Collagenase	1HFC/P03956	Metalloprotease	-7.3±0.0
L-Asparaginase	1HFW/P06608	Asparaginase	-6.2±0.0
Hypoxanthine-guanine-xanthine Phosphoribosyltransferase	1HGX/P51900	Transferase (glycosyltransferase)	-7.3±0.0
Human Class I Histocompatibility Antigen	1HHJ/P01892	Histocompatibility Antigen	**-8.3±0.0***
HIV-1 Protease	1HIH/P03366	Hydrolase (aspartic Proteinase)	-7.7±0.0
Diaminopimelate Decarboxylase	1HKV/P9WIU7	Lyase	-7.6±0.0
Fucosidase alpha	1HL9/Q9WYE2	Hydrolase	-7.6±0.0
Alcohol Dehydrogenase	1HLD/P00327	Oxidoreductase(ch Oh(d) Nad(a))	**-8.4±0.0***
Hypoxanthine-Guanine Phosphoribosyltransferase	1HMP/P00492	Transferase (glycosyltransferase)	-7.0±0.0
Prophospholipase A2	1HN4/P00592	Hydrolase	**-9.3±0.0***
Glutathione S-transferase	1HNA/P28161	Transferase (glutathione)	-6.3±0.0
Human Neutrophil Elastase	1HNE/P08246	Hydrolase/hydrolase Inhibitor	-6.1±0.0
HIV-1 Reverse Transcriptase	1HNI/P03366	Nucleotidyltransferase	-7.0±0.0
Phenylethanolamine N-Methyltransferase	1HNN/P11086	Transferase	-6.5±0.0
Pyridoxine 5'-Phosphate Synthase	1HO4/P0A794	Biosynthetic Protein	-7.7±0.0
HIV-1 Protease	1HOS/P03366	Hydrolase(acid Proteinase)	-7.5±0.0
Beta-N-acetylhexosaminidase	1HP5/O85361	Hydrolase	-7.7±0.0
Glutamic Acid-Specific Protease	1HPG/Q07006	Hydrolase/hydrolase Inhibitor	-6.6±0.0

Adenosinetriphosphatase	1HPM/P19120	Hydrolase (acting On Acid Anhydrides)	-7.3±0.0
HIV-1 Protease	1HPS/P03366	Hydrolase(acid Proteinase)	**-8.2±0.0***
Human Rhinovirus 14	1HRI/P03303	Virus	-7.3±0.0
Leukotriene A4 hydrolase	1HS6/P09960	Hydrolase	**-9.5±0.0***
Prostaglandin G/H Synthase 1, COX-1	1HT8/P05979	Oxidoreductase	**-8.2±0.0***
UDP-n-acetylglucosamine Pyrophosphorylase	1HV9/P0ACC7	Transferase	-7.7±0.0
D-alanyl-D-alanine Carboxypeptidase	1HVB/P15555	Hydrolase	-7.6±0.0
Estrogen Sulfotransferase	1HY3/P49888	Transferase	-7.0±0.0
Thermolysin	1HYT/P00800	Hydrolase(metalloproteinase)	-7.8±0.0
Riboflavin Synthase	1HZE/P0AFU8	Transferase	-7.1±0.0
Riboflavin alpha synthase	1I18/P0AFU8	Transferase	-7.5±0.0
Precorrin-8X Methylmutase	1I1H/P21638	Isomerase	-6.3±0.0
4-Amino-4-Deoxychorismate Lyase	1I2K/P28305	Lyase	-7.0±0.0
Insulin Receptor	1I44/P06213	Transferase	-7.1±0.0
Carbonic anhydrase	1I6O/P61517	Lyase	-6.6±0.0
DNA-directed RNA Polymerase II 19 KDa Polypeptide	1I6V/Q9KWU8	Transcription	-7.6±0.0
Apolipoprotein(a)	1I71/P08519	Hydrolase	-5.7±0.0
Neutrophil Collagenase, MMP8	1I76/P22894	Hydrolase	**-8.1±0.0***
Methionine decarboxylase	1I7B/P17707	Lyase	**-8.4±0.0***
Riboflavin Synthase	1I8D/P0AFU8	Transferase	-7.2±0.0
Glutamate Mutase	1I9C/P80078	Isomerase	-7.5±0.0
Phosphatidylinositol phosphatase	1I9Z/O43001	Hydrolase	-6.7±0.0
Dihydrofolate Reductase	1IA1/P22906	Oxidoreductase	-7.8±0.0
Transient Receptor Potential-Related Protein,Atypical protein kinase	1IA9/Q923J1	Transferase	-7.8±0.0
1-Aminocyclopropane-1-Carboxylate Synthase 2	1IAY/P18485	Lyase	-7.4±0.0
14-3-3 Zeta: Serotonin	1IB1/P63104	Signaling Protein/transferase	-7.3±0.0
Cystathionine beta-lyase	1IBJ/P53780	Lyase	-7.8±0.0
Interleukin-1 Beta Converting Enzyme	1ICE/P29466	Hydrolase/hydrolase Inhibitor	-6.2±0.0
12-Oxophytodienoate Reductase 1	1ICP/Q9XG54	Oxidoreductase	-7.6±0.0
Nitroreductase	1ICR/P38489	Oxidoreductase	-7.8±0.0

HIV-2 Protease	1IDA/P04584	Hydrolase/hydrolase Inhibitor	-7.6±0.0
NH(3)-dependent NAD(+) Synthetase	1IFX/P08164	Ligase	-7.5±0.0
Thiamin Pyrophosphokinase	1IG0/P35202	Transferase	-7.9±0.0
Thiamin Pyrophosphokinase	1IG3/Q9R0M5	Transferase	**-8.4±0.0***
Cyclophilin 40	1IHG/P26882	Isomerase	-6.7±0.0
InaD	1IHJ/Q24008	Signaling Protein	-6.2±0.0
Peptide N-myristoyltransferase	1IIC/P14743	Transferase	-7.5±0.0
Cholesterol Oxidase	1IJH/P12676	Oxidoreductase	**-8.9±0.0***
Histidinol Phosphate Aminotransferase	1IJI/P06986	Transferase	-6.9±0.0
Pol Polyprotein	1IKX/P03366	Transferase	-6.7±0.0
Inositol monophosphatase	1IMB/P29218	Hydrolase	-7.7±0.0
Holliday Junction DNA Helicase Ruvb	1IN4/Q56313	DNA Binding Protein	-7.1±0.0
Influenza A Subtype N2 Neuraminidase	1ING/P06820	Hydrolase (o Glycosyl)	-6.8±0.0
Dipeptidase	1ITU/P16444	Hydrolase	-6.8±0.0
Phosphotransketolase	1ITZ/Q7SIC9	Transferase	-6.8±0.0
Microbial Transglutaminase	1IU4/P81453	Transferase	-7.3±0.0
Influenza A Subtype N2 Neuraminidase	1IVD/P06820	Hydrolase (o Glycosyl)	-7.6±0.0
Neuraminidase	1IVE/P06820	Hydrolase (o Glycosyl)	-7.1±0.0
Aspartate Aminotransferase, Cytoplasmic	1IX6/P00509	Transferase	-7.5±0.0
Branched Chain Amino Acid-dependent Aminotransferase	1IYD/P0AB80	Transferase	-6.7±0.0
Hematopoietic Prostagladin D Synthase	1IYH/O60760	Isomerase	-7.8±0.0
Oxygenase	1J02/P06762	Oxidoreductase	-6.2±0.0
1-Aminocyclopropane-1-Carboxylate Deaminase	1J0E/Q7M523	Lyase	-7.8±0.0
Glycogen synthase kinase-3 beta	1J1B/P49841	Transferase	-7.8±0.0
Aspartate Aminotransferase, Cytoplasmic	1J32/Q8RR70	Transferase	-7.0±0.0
cAMP-dependent protein kinase catalytic subunit	1J3H/P05132	Transferase	-6.9±0.0
Fk506-binding Protein 1a	1J4R/P62942	Isomerase	-6.4±0.0
Heme oxygenase	1J77/Q9RGD9	Oxidoreductase	-6.2±0.0
Deoxyribonucleoside Kinase	1J90/Q9XZT6	Transferase	-7.2±0.0
Aldo-Keto Reductase Family 1 Member C3	1J96/P52895	Oxidoreductase	**-8.9±0.0***
Alcohol Sulfotransferase	1J99/Q06520	Transferase	-7.7±0.0

Metalloprotease 8	1JAP/P22894	Complex (metalloprotease/inhibitor)	-6.6±0.0
CAMP-DEPENDENT PROTEIN KINASE, ALPHA-CATALYTIC SUBUNIT	1JBP/P05132	Transferase	-7.4±0.0
Cystathionine beta-synthase	1JBQ/P35520	Lyase	-7.8±0.0
IMP-1 dehydrogenase	1JCN/P20839	Oxidoreductase	-7.8±0.0
L-Isoaspartyl D-aspartyl O-methyltransferase	1JG1/Q8TZR3	Transferase	-5.9±0.0
Amylosucrase	1JGI/Q9ZEU2	Transferase	-7.2±0.0
Cyclic nucleotide phosphodiesterase	1JH7/O04147	Hydrolase	-7.9±0.0
Neutrophil-Activating Protein A	1JI4/P43313	Metal Transport	-6.9±0.0
Porcine Pancreatic Elastase	1JIM/P00772	Hydrolase(serine Proteinase)	-7.2±0.0
Phenylalanyl-tRNA Synthetase	1JJC/Q5SGX2	Ligase	-7.7±0.0
HIV Protease	1JKH/P04585	Transferase	**-8.0±0.0***
Death-Associated Protein Kinase	1JKL/P53355	Transferase	-7.0±0.0
Glycinamide Ribonucleotide Formyltransferase	1JKX/P08179	Transferase	-6.8±0.0
cAMP-dependent Protein Kinase	1JLU/P05132	Transferase/transferase Inhibitor	-7.7±0.0
Jun N terminal kinase	1JNK/P53779	Transferase	-6.9±0.0
Pyridoxine 5'-Phosphate Oxidase	1JNW/P0AFI7	Oxidoreductase	-6.5±0.0
NADH Peroxidase	1JOA/P37062	Oxidoreductase	-7.9±0.0
Dihydrofolate Reductase	1JOM/P0ABQ4	Oxidoreductase	-6.6±0.0
Methylthioadenosine phosphorylase	1JP7/P50389	Transferase	**-8.2±0.0***
Phospholipase A2	1JQ9/P59071	Hydrolase/hydrolase Inhibitor	-7.9±0.0
Histamine N-methyltransferase	1JQE/P50135	Transferase	**-8.4±0.0***
Insulin-like Growth Factor I Receptor	1JQH/P08069	Transferase	-6.8±0.0
Inosine-5'-monophosphate Dehydrogenase 2,IMP-2 dehydrogenase	1JR1/P12269	Oxidoreductase	**-8.4±0.0***
Catechol O-Methyltransferase	1JR4/P22734	Transferase	-6.9±0.0
Aromatic-L-amino-acid Decarboxylase	1JS3/P80041	Lyase	**-8.4±0.0***
Dopa decarboxylase	1JS6/P80041	Lyase	-7.6±0.0
Hydroxysteroid-Dehydrogenase Type 1	1JTV/P14061	Oxidoreductase	-7.4±0.0
Dihydroorotate Dehydrogenase	1JUE/A2RJT9	Oxidoreductase	**-8.9±0.0***
DXP reductoisomerase	1JVS/P45568	Oxidoreductase	-7.3±0.0
Casein Kinase II, Alpha Chain	1JWH/P68400	Transferase	-7.2±0.0

Prothrombin	1JWT/P00734	Hydrolase	-7.7±0.0
Methylthioadenosine nucleosidase,S-Adenosylhomocysteine nucleosidase	1JYS/P0AF12	Hydrolase	-6.6±0.0
NADPH Dehydrogenase	1K02/Q02899	Oxidoreductase	**-9.8±0.0***
Tryptophan Synthase Alpha chain	1K3U/P00929	Lyase	-7.1±0.0
Glutathione Transferase A1-1	1K3Y/P08263	Transferase	**-8.9±0.0***
Nicotinate-nucleotide Adenylyltransferase	1K4M/P0A752	Transferase	**-8.3±0.0***
Cytosine deaminase	1K6W/P25524	Hydrolase	-7.8±0.0
5-methyltetrahydrofolate--homocysteine Methyltransferase,Methionine Synthase	1K7Y/P13009	Transferase	-7.4±0.0
Caspase-7	1K86/P55210	Apoptosis	-6.6±0.0
Triacylglycerol Lipase, Pancreatic	1K8Q/P80035	Hydrolase	-7.8±0.0
Transforming Protein P21/H-Ras-1	1K8R/P01112	Signaling Protein	**-8.6±0.0***
Histidinol Dehydrogenase	1KAE/P06988	Oxidoreductase	**-8.1±0.0***
Neutrophil Collagenase	1KBC/P22894	Metalloproteinase	-7.1±0.0
Thymidine Kinase	1KIM/P03176	Transferase	-6.9±0.0
Urease	1KRA/P18316	Hydrolase (urea Amido)	-7.3±0.0
Alpha-N-acetylgalactosaminidase	1KTB/Q90744	Hydrolase	-6.5±0.0
Tgf-beta Type II Receptor	1KTZ/P10600	Cytokine/cytokine Receptor	-6.3±0.0
Mycolic Acid Synthase	1L1E/P9WPB3	Transferase	**-8.2±0.0***
CMV protease	1LAY/P16753	Serine Protease	-6.8±0.0
Inositol 1 phosphatase	1LBV/O30298	Hydrolase	**-8.2±0.0***
Lysophospholipase	1LCL/Q05315	Serine Esterase	-6.0±0.0
Adipocyte-derived Leucine Aminopeptidase	1LCP/P00727	Hydrolase (alpha Aminoacylpeptide)	-7.5±0.0
Alcohol Dehydrogenase	1LDE/P00327	Dehydrogenase	**-8.2±0.0***
L-lactate Dehydrogenase	1LDM/P00341	Oxidoreductase	-7.9±0.0
Alcohol Dehydrogenase	1LDY/P00327	Dehydrogenase	-7.6±0.0
Glutamine Synthetase	1LGR/P0A1P6	Ligase(amide Synthetase)	-7.4±0.0
Lysozyme	1LMO/P11941	Hydrolase (o Glycosyl)	-7.1±0.0
Thermolysin	1LNA/P00800	Metalloprotease	-7.9±0.0
Casein Kinase II, Protein Kinase Ck2	1LP4/P28523	Transferase	**-9.0±0.0***
Triacylglycerol Lipase, Pancreatic	1LPM/P20261	Hydrolase	-7.1±0.0
Homocysteine S methyltransferase	1LT8/Q93088	Transferase	-6.7±0.0

ZC protein kinase	1LVG/Q64520	Transferase	-7.8±0.0
Cathepsin D	1LYW/P07339	Aspartic Protease	-7.6±0.0
Memapsin 2 (Beta-Secretase)	1M4H/P56817	Hydrolase/hydrolase Inhibitor	**-8.4±0.0***
Caspase-1	1M72/P89116	Hydrolase/hydrolase Inhibitor	-7.0±0.0
Nitric Oxide Synthase	1M9M/P29474	Oxidoreductase	**-8.6±0.0***
Tgf-beta Type II Receptor	1M9Z/P37173	Hormone/growth Factor	-6.7±0.0
Mandelate Racemase	1MDR/P11444	Racemase	-7.2±0.0
Inosine-5'-Monophosphate Dehydrogenase	1ME8/P50097	Oxidoreductase	-7.8±0.0
Matrilysin	1MMQ/P09237	Metalloprotease	-7.1±0.0
ATPase	1MO7/P06685	Hydrolase	-6.9±0.0
Glucosamine-6-Phosphate Synthase	1MOQ/P17169	Glutamine Amidotransferase	-7.7±0.0
Cytidine deaminase	1MQ0/P32320	Hydrolase	-7.4±0.0
Alpha-Momorcharin	1MRG/P16094	Ribosome Inactivating Protein	-7.0±0.0
Alpha-Trichosanthin	1MRK/P09989	Ribosome Inactivating Protein	-7.9±0.0
Aurora-2 protein kinase	1MUO/O14965	Transferase	-7.7±0.0
Phenylethanolamine N-Methyltransferase	1N7I/P11086	Transferase	-6.6±0.0
Lysyl oxidase	1N9E/Q96X16	Oxidoreductase	-7.9±0.0
Carnitine Acetyltransferase	1NDF/P47934	Transferase	-7.8±0.0
Succinate dehydrogenase	1NEK/P0AC41	Oxidoreductase/electron Transport	-7.5±0.0
Carnitine acyltransferase	1NM8/P43155	Transferase	**-8.1±0.0***
Chymase	1NN6/P23946	Hydrolase	-7.4±0.0
Neuramidase	1NNB/P03472	Hydrolase(o Glycosyl)	-7.4±0.0
Neuraminidase N9	1NNC/P03472	Hydrolase (o Glucosyl)	-7.9±0.0
3-dehydroquinate Synthase	1NR5/P07547	Lyase	**-8.4±0.0***
Neuramidase	1NSC/P27907	Hydrolase(o Glycosyl)	-7.7±0.0
MAP kinase-activated protein kinase 2	1NY3/P49137	Transferase	-7.7±0.0
Tyrosine-protein kinase transforming protein SRC	1NZL/P00524	Transferase	-6.1±0.0
Aspartate Aminotransferase, Cytoplasmic,Aspartate Transaminase	1O4S/Q9X0Y2	Transferase	-7.4±0.0
Activated Akt/Protein Kinase B	1O6L/P31751	Transferase	-7.7±0.0
Alpha mannosidase	1O7D/Q29451	Hydrolase	**-8.0±0.0***
Fibroblast Growth Factor Receptor 2	1OEC/P21802	Transferase	**-8.1±0.0***

Aldehyde Dehydrogenase, Mitochondrial	1OF7/P05091	Oxidoreductase	-7.1±0.0
Cytochrome P450 2C9	1OG5/P11712	Electron Transport	-7.4±0.0
Glucosylceramidase	1OGS/P04062	Hydrolase	-7.1±0.0
4-AMINOBUTYRATE AMINOTRANSFERASE	1OHY/P80147	Transferase	-7.2±0.0
Cell Division Protein Kinase 2	1OI9/P24941	Kinase	-7.9±0.0
Cell Division Protein Kinase 2	1OIU/P24941	Kinase	**-8.5±0.0***
Hyaluronate Lyase	1OJN/Q54873	Lyase	-7.6±0.0
Carbonic Anhydrase II	1OKL/P00918	Lyase	-6.8±0.0
3-methyl-2-oxobutanoate Dehydrogenase (Lipoamide)	1OLS/P12694	Oxidoreductase	-7.9±0.0
Oxygen-insensitive NAD(P)H nitroreductase	1OOQ/P38489	Oxidoreductase	**-8.1±0.0***
Peptidylglycine Alpha-Hydroxylating Monooxyg	1OPM/P14925	Oxidoreductase	-7.5±0.0
Ornithine Decarboxylase	1ORD/P43099	Carboxy Lyase	-7.6±0.0
Thermolysin	1OS0/P00800	Hydrolase/hydrolase Inhibitor	-7.8±0.0
Hepatitis C virus NS5B polymerase	1OS5/P26663	Transferase	-7.4±0.0
Dihydroorotate Dehydrogenase, Mitochondrial	1OVD/A2RJT9	Oxidoreductase	-7.8±0.0
Indole-3-Pyruvate Decarboxylase	1OVM/P23234	Lyase	-7.7±0.0
Aspartate Aminotransferase, Cytoplasmic	1OXO/P00508	Aminotransferase	-7.5±0.0
NADPH Dehydrogenase	1OYA/Q02899	Oxidoreductase (flavoprotein)	**-8.4±0.0***
Acetolactate Synthase, Catabolic	1OZF/P27696	Lyase	-7.7±0.0
Isopentenyl-diphosphate delta-isomerase	1P0N/P50740	Isomerase	-7.0±0.0
Glycogen Phosphorylase, Muscle Form	1P2D/P00489	Transferase	**-8.9±0.0***
p38 MAP kinase	1P38/P47811	Transferase	-7.3±0.0
glutamyl-endopeptidase	1P3C/Q9EXR9	Hydrolase	-6.9±0.0
Heme oxygenase 1	1P3U/Q9RGD9	Oxidoreductase	-6.4±0.0
Cysteine desulfurase	1P3W/P0A6B7	Lyase	-6.6±0.0
N-acetylglucosamine deacetylase	1P42/O67648	Hydrolase	-7.5±0.0
Glycogen phosphorylase, muscle form	1P4G/P00489	Transferase	-7.4±0.0
Riboflavin Kinase	1P4M/Q969G6	Transferase	-7.4±0.0
Thrombin	1P8V/P07359	Membrane Protein/hydrolase	-7.6±0.0
P-Hydroxybenzoate Hydroxylase	1PBD/P00438	Oxidoreductase	**-8.6±0.0***
Purine Nucleoside Phosphorylase	1PBN/P55859	Pentosyltransferase	-7.0±0.0

Coagulation Factor IX	1PFX/P16293	Hydrolase/hydrolase Inhibitor	-7.3±0.0
Aspartate Carbamoyltransferase	1PG5/Q55338	Transferase	-7.8±0.0
6-phosphogluconate Dehydrogenase	1PGP/P00349	Oxidoreductase (choh(d) Nadp+(a))	-6.7±0.0
Cytochrome P450-Cam	1PHD/ P00183	Oxidoreductase(oxygenase)	**-8.0±0.0***
Phosphatidylinositol 3-kinase	1PIC/P27986	Complex (phosphotransferase/receptor)	**-8.0±0.0***
alpha-Amylase	1PIG/P00690	Glycosyltransferase	-7.9±0.0
Cis-trans isomerase	1PIN/Q13526	Isomerase	-7.2±0.0
N, N-Dimethylglycine Oxidase	1PJ6/Q9AGP8	Oxidoreductase	**-8.9±0.0***
Casein Kinase II, Alpha Chain	1PJK/P68400	Transferase	**-8.5±0.0***
Sorbitol dehydrogenase	1PL7/Q00796	Oxidoreductase	-7.6±0.0
Glutathione transferase	1PMT/P15214	Transferase	-6.9±0.0
Phospholipase A2	1POC/P00630	Hydrolase	-7.9±0.0
Trypsin	1PPC/P00760	Hydrolase/hydrolase Inhibitor	-6.9±0.0
Trypsin	1PPH/P00760	Hydrolase/hydrolase Inhibitor	-6.7±0.0
alpha-Amylase	1PPI/P00690	Hydrolase (o Glycosyl)	-7.9±0.0
Penicillopepsin	1PPK/P00798	Hydrolase/hydrolase Inhibitor	-7.6±0.0
Cytochrome P450 2C8	1PQ2/P10632	Oxidoreductase	-7.6±0.0
Arginase II, mitochondrial precursor	1PQ3/P78540	Hydrolase	-6.8±0.0
2,4-Dienoyl-CoA Reductase	1PS9/P42593	Oxidoreductase	**-8.3±0.0***
Pepsin 3A	1PSO/P0DJD7	Hydrolase/hydrolase Inhibitor	-7.5±0.0
Phosphatidylinositol-Specific Phosphodiesterase C	1PTG/P14262	Hydrolase (phosphoric Diester)	-7.0±0.0
Protein kinase C delta	1PTR/P28867	Phosphotransferase	-5.8±0.0
Protein Tyrosine Phosphatase 1b	1PTY/P18031	Hydrolase	-7.9±0.0
DNA Topoisomerase II	1PVG/P06786	Isomerase	**-9.4±0.0***
Ornithine carbamoyltransferase	1PVV/Q51742	Transferase	-7.5±0.0
3,4-dihydroxy-2-butanone-4-phosphate Synthase	1PVY/Q60364	Isomerase	-6.3±0.0
Cyclin-dependent kinase-2	1PW2/P24941	Transferase	-6.8±0.0
Prostaglandin G/H Synthase 2	1PXX/Q05769	Oxidoreductase	**-9.1±0.0***
TGF-beta receptor type I	1PY5/P36897	Transferase	**-8.7±0.0***
Penicillin Binding Protein 2	1PYY/P59676	Transpeptidase	-7.8±0.0

2-amino-4-hydroxy-6-hydroxymethyldihydropteridine pyrophosphokinase	1Q0N/P26281	Transferase	**-8.3±0.0***
Histone deacetylase-2	1Q1A/P53686	Gene Regulation	-7.3±0.0
Intestinal Glcnac-6-sulfotransferase	1Q1Z/O00204	Transferase	-7.9±0.0
Steroid Sulphotransferase	1Q44/P52839	Transferase	-6.9±0.0
PLK-1 protein kinase	1Q4O/P53350	Transferase	-6.9±0.0
Prostaglandin-E2 9-reductase	1Q5M/P80508	Oxidoreductase	**-9.5±0.0***
3-hydroxy-3-methylglutaryl-coenzyme A Reductase	1QAX/P13702	Oxidoreductase	**-8.3±0.0***
Pokeweed Antiviral Protein	1QCI/P10297	Antiviral Protein	-7.4±0.0
Phospholipase A1	1QD6/P0A921	Membrane Protein	**-8.0±0.0***
Pentosyltransferase	1QE5/P81989	Transferase	-7.4±0.0
ATP-Dependent Helicase Pcra	1QHG/P56255	Hydrolase	-7.5±0.0
Thymidine Kinase	1QHI/P03176	Transferase	-6.9±0.0
Stromelysin	1QIA/P08254	Hydrolase	**-8.1±0.0***
Acetylcholinesterase	1QIJ/P04058	Hydrolase	-6.8±0.0
Lactoylglutathione lyase	1QIP/Q04760	Lyase	-7.8±0.0
Isopenicillin N Synthetase	1QIQ/P05326	Antibiotic Biosynthesis	**-8.2±0.0***
Ketosteroid Isomerase	1QJG/P00947	Isomerase	-6.7±0.0
Esterase	1QLW/Q7SIA5	Hydrolase(carboxylic Esterase)	-7.2±0.0
Methionine adenosyltransferase	1QM4/P13444	Transferase	-6.9±0.0
Thioredoxin peroxidase	1QMV/P32119	Oxidoreductase	-7.1±0.0
Elastase	1QNJ/P00772	Hydrolase (serine Protease)	-7.3±0.0
Pyruvate Decarboxylase	1QPB/P06169	Lyase	**-9.2±0.0***
LCK kinase	1QPC/P06239	Transferase	-7.2±0.0
LCK Tyrosine Kinase	1QPJ/P06239	Transferase	-7.7±0.0
Quinone reductase-2	1QR2/P16083	Oxidoreductase	-6.4±0.0
Quinone reductase	1QRD/P05982	Quinone Reductase (cytosolic)	-7.1±0.0
HIV-1 Integrase	1QS4/P12497	Transferase	-6.4±0.0
Ornithine Decarboxylase	1QU4/P07805	Lyase	-7.9±0.0
Hepatocyte Growth Factor Receptor	1R0P/P08581	Transferase	-7.2±0.0
ADP-ribosyl cyclase	1R12/P29241	Hydrolase	**-8.0±0.0***
Methyltransferase	1R18/Q27869	Transferase	**-8.1±0.0***

3-hydroxy-3-methylglutaryl-coenzyme A reductase	1R31/P13702	Oxidoreductase	-7.4±0.0
Lipase	1R50/P37957	Hydrolase	-5.9±0.0
Chorismate synthase	1R53/P28777	Lyase	-6.7±0.0
ADAM-33	1R55/Q9BZ11	Hydrolase	-7.1±0.0
Glycosyltransferase	1R82/P16442	Transferase	-6.7±0.0
Cytochrome P450 2C9	1R9O/P11712	Oxidoreductase	-7.8±0.0
Dihydrofolate Reductase	1RA2/P0ABQ4	Oxidoreductase	-7.0±0.0
CT610	1RCW/O84616	Oxidoreductase	-6.0±0.0
Methylmalonyl-coa Mutase	1REQ/P11653	Isomerase	**-8.0±0.0***
Coagulation Factor IX	1RFN/P00740	Coagulation Factor	-7.0±0.0
Ribonuclease	1RGE/P05798	Hydrolase (guanyloribonuclease)	-6.3±0.0
Renin	1RNE/ P00797	Hydrolase(acid Proteinase)	**-8.9±0.0***
Ribonuclease A	1ROB/P61823	Hydrolase(endoribonuclease)	-7.0±0.0
Deoxyhypusine synthase	1ROZ/P49366	Transferase	-6.8±0.0
HIV-1 Reverse Transcriptase	1RT6/P04585	Nucleotidyltransferase	**-8.9±0.0***
DNA Polymerase/reverse Transcriptase,HIV-1 Reverse Transcriptase	1RTD/P03366	Transferase/dna	-6.7±0.0
Murine Double Minute-2 (MDM2)	1RV1/Q00987	Ligase	-5.9±0.0
Mitogen-Activated Protein Kinase Kinase 2 (MEK2)	1S9I/P36507	Transferase	-7.5±0.0
Protoporphyrinogen oxidase	1SEZ/O24164	Oxidoreductase	**-8.5±0.0***
4-aminobutyrate aminotransferase	1SFF/P22256	Transferase	-7.6±0.0
Alanine Racemase	1SFT/P10724	Isomerase	-7.1±0.0
NRH dehydrogenase [quinone] 2	1SG0/P16083	Oxidoreductase	**-9.2±0.0***
Tyrosine-protein kinase ITK/TSK	1SM2/Q08881	Transferase	-7.8±0.0
Amylase	1SMD/P04745	Hydrolase (o Glycosyl)	-7.6±0.0
Thermonuclease Precursor	1SNC/P00644	Hydrolase (phosphoric Diester)	-7.9±0.0
Adenylosuccinate synthetase	1SON/P0A7D4	Ligase	**-8.1±0.0***
Pantothenate kinase	1SQ5/P0A6I3	Transferase	**-8.2±0.0***
Nuclease	1STB/P00644	Hydrolase(phosphoric Diester)	-6.5±0.0
Camp-dependent Protein Kinase A Type I	1STC/P00517	Complex (transferase/inhibitor)	**-8.2±0.0***
Glucokinase	1SZ2/P0A6V9	Transferase	-7.3±0.0

Alpha-galactosidase	1SZN/P0A6V9	Transferase	-6.7±0.0
Atrial Natriuretic Peptide Receptor A,Natriuretic peptide receptor A	1T34/P18910	Signaling Protein	**-8.2±0.0***
Aldehyde Reductase	1T41/P15121	Oxidoreductase	**-9.4±0.0***
Kit tyrosine kinase	1T46/P10721	Transferase Activator	**-8.0±0.0***
TAQ DNA Polymerase	1TAQ/P19821	Nucleotidyltransferase	-7.8±0.0
Hypoxanthine phosphoribosyltransferase	1TC1/Q4DRC4	Transferase	-7.6±0.0
Serine/Threonine Phosphatase B2	1TCO/P48452	Complex (hydrolase/isomerase)	-7.9±0.0
HIV Protease	1TCX/P04587	Hydrolase (acid Protease)	-7.6±0.0
Thymidylate Synthetase	1TDB/P00469	Transferase (methyltransferase)	-7.2±0.0
Threonine Deaminase	1TDJ/P04968	Allostery	-7.8±0.0
Beta-galactosidase	1TG7/Q700S9	Hydrolase	**-8.5±0.0***
Thymidylate Synthase	1TIS/P00471	Transferase(methyltransferase)	-7.9±0.0
S-adenosylmethionine decarboxylase	1TLU/Q9WZC3	Lyase	-5.7±0.0
Thermolysin	1TMN/P00800	Hydrolase/hydrolase Inhibitor	-7.8±0.0
Trypsin	1TNG/P00760	Hydrolase/hydrolase Inhibitor	-6.8±0.0
Tyrosine hydroxylase	1TOH/P04177	Hydroxylase	**-8.5±0.0***
Triosephosphate Isomerase	1TPH/P00940	Triosephosphate Isomerase	-6.7±0.0
Tyrosine Phenol-Lyase	1TPL/P31013	Lyase(carbon Carbon)	-7.5±0.0
Beta-Trypsin	1TPP/P00760	Hydrolase/hydrolase Inhibitor	-6.9±0.0
Beta-Secretase	1TQF/P56817	Hydrolase	-7.2±0.0
Thymidylate Synthetase	1TSD/P0A884	Transferase (methyltransferase)	-7.9±0.0
Tubulin	1TUB/P02550	Microtubules	**-8.1±0.0***
Reverse Transcriptase	1TVR/P03366	Aspartyl Protease	-7.8±0.0
Dihydropteroate Synthetase	1TX0/Q81VW8	Transferase	-7.3±0.0
P50-Rhogap	1TX4/Q07960	Complex (gtpase Activatn/proto Oncogene)	-7.9±0.0
Trypanothione Reductase	1TYP/P39040	Oxidoreductase	**-8.6±0.0***
Chalcone synthase 2	1U0W/P30074	Transferase	**-8.1±0.0***
L-Lactate dehydrogenase	1U4O/Q27743	Oxidoreductase	-7.0±0.0
Uracil-DNA Glycosylase	1UDH/P10186	Hydrolase	-7.1±0.0
Metalloprotease 3	1UEA/P08254	Complex (metalloprotease/inhibitor)	-7.8±0.0

Enoyl ACP reductase	1UH5/Q9BJJ9	Oxidoreductase	**-8.4±0.0***
Threonine Synthase	1UIM/P83823	Lyase	-7.7±0.0
SARS Coronavirus Main Proteinase (SARS-CoV 3CLpro)	1UK4/P0C6X7	Hydrolase	-7.2±0.0
Uridylmonophosphate/Cytidylmonophosphate Kinase	1UKE/P20425	Nucleotide Monophosphate Kinase	-6.1±0.0
c-jun N-terminal kinase (JNK1)	1UKI/P45983	Transferase	-7.4±0.0
Uridylate Kinase	1UKZ/P15700	Transferase	-6.3±0.0
Purine Nucleoside Phosphorylase	1ULB/P00491	Pentosyltransferase	-7.4±0.0
Aldose Reductase	1US0/P15121	Oxidoreductase	-7.9±0.0
5'-Nucleotidase	1USH/P07024	Hydrolase	-7.6±0.0
PDK1 protein kinase	1UU7/O15530	Transferase	-7.3±0.0
Thrombin	1UVT/P00735	Serine Protease	**-8.2±0.0***
Glyceraldehyde-3-phosphate dehydrogenase	1UXV/O57693	Oxidoreductase	-7.5±0.0
DNA primase small subunit	1V33/O57934	Transferase	-6.9±0.0
Glucokinase Isoform 2	1V4S/P35557	Transferase	-7.2±0.0
Adenosylhomocysteinase	1V8B/P50250	Hydrolase	**-8.6±0.0***
Arylesterase	1VA4/P22862	Hydrolase	-6.5±0.0
Acylamino-acid-releasing enzyme	1VE6/Q9YBQ2	Hydrolase	-7.9±0.0
D-amino acid oxidase	1VE9/P00371	Oxidoreductase	**-9.2±0.0***
Beta-amylase	1VEM/P36924	Hydrolase	**-8.3±0.0***
NAD(P)H\:FMN Oxidoreductase	1VFR/P46072	Oxidoreductase	-7.9±0.0
Shikimate kinase	1VIA/Q0PBC3	Transferase	-6.3±0.0
Catechol O-Methyltransferase	1VID/P22734	Transferase (methyltransferase)	-6.8±0.0
Urokinase	1VJA/P00749	Hydrolase	-6.8±0.0
Alpha glucosidase	1VJT/Q9WZL1	Hydrolase	-7.9±0.0
Histone deacetylase-8	1VKG/Q9BY41	Hydrolase	-7.9±0.0
Aldehyde oxidoreductase	1VLB/Q46509	Oxidoreductase	**-9.9±0.0***
Acetylcholinesterase	1VOT/P04058	Hydrolase	-7.9±0.0
ABC transporter, ATP-binding protein	1VPL/Q9WZ14	Lyase	-6.9±0.0
Creatine Kinase, M chain	1VRP/P04414	Transferase	-7.1±0.0
Thymidine Kinase	1VTK/P03176	Transferase	-6.9±0.0

Acetylcholinesterase	1VXR/P04058	Hydrolase	**-8.8±0.0***
Acyl CoA oxidase-1	1W07/O65202	Oxidoreductase	**-8.4±0.0***
Lysozyme	1W08/P61626	Hydrolase	-6.7±0.0
Beta-secretase-1	1W50/P56817	Hydrolase	-6.6±0.0
Phenylethylamine Oxidase	1W6G/P46881	Oxidoreductase	-7.2±0.0
Lanosterol Synthase	1W6K/P48449	Isomerase	**-8.6±0.0***
Kynurenine oxoglutarate transaminase	1W7L/Q16773	Transferase	-7.6±0.0
Platelet-activating Factor Acetylhydrolase	1WAB/Q29460	Platelet Factor	-6.8±0.0
Protein Tyrosine Phosphatase 1b	1WAX/P18031	Hydrolase	-6.4±0.0
Protein arginine deiminase IV	1WDA/Q9UM07	Hydrolase	-6.6±0.0
A-G adenine DNA glycosylase	1WEI/P17802	Hydrolase	**-8.1±0.0***
Transglycosylase	1WKD/P28720	TRNA Modifying Enzyme	**-8.2±0.0***
Guanine deaminase	1WKQ/O34598	Hydrolase	-6.7±0.0
Polyporopepsin	1WKR/P17576	Hydrolase/hydrolase Inhibitor	-7.1±0.0
Isoleucyl tRNA synthetase	1WNY/P56690	Ligase	**-8.1±0.0***
Agmatinase	1WOH/Q9RZ04	Hydrolase	-7.5±0.0
Ras-Ras Gap	1WQ1/P01112	Complex (gtp Binding/gtpase Activation)	**-8.9±0.0***
Uricase	1WRR/Q00511	Oxidoreductase	-7.0±0.0
GTP cyclohydrolase-I	1WUR/Q5SH52	Hydrolase	-6.7±0.0
TrkA receptor	1WWA/P04629	Transferase	-5.7±0.0
TrkB receptor	1WWB/Q16620	Transferase	-7.1±0.0
TrkC receptor	1WWC/Q16288	Transferase	-6.6±0.0
Tyrosinase	1WXC/Q83WS2	Oxidoreductase/metal Transport	-7.1±0.0
Protein geranylgeranyl transferase	1WY0/O58799	Transferase	-6.9±0.0
Undecaprenyl diphosphate synthetase	1X07/P60472	Transferase	**-8.1±0.0***
Syk tyrosine kinase	1XBA/P43405	Transferase	-7.1±0.0
hypothetical protein PA0115	1XEB/Q9I717	Transferase	**-8.1±0.0***
Glutamate decarboxylase	1XEY/P69908	Lyase	-7.2±0.0
putative acetyltransferase/acyltransferase	1XHD/Q816R4	Transferase	-6.6±0.0
Alanine transaminase	1XI9/Q9P9M8	Transferase	-6.8±0.0

D-Xylose Isomerase	1XID/P24300	Isomerase(intramolecular Oxidoreductase)	-7.9±0.0
Coagulation Factor Xa	1XKA/P00742	Blood Coagulation Factor	-7.8±0.0
Epidermal Growth Factor Receptor(EGFR)	1XKK/P00533	Transferase	**-9.3±0.0***
Peroxisomal carnitine O-octanoyltransferase	1XMC/Q9DC50	Transferase	**-8.4±0.0***
Thrombin light chain	1XMN/P00734	Hydrolase/hydrolase Inhibitor	-6.7±0.0
cAMP-specific 3',5'-cyclic phosphodiesterase 4D	1XOQ/Q08499	Hydrolase	**-8.1±0.0***
cAMP-specific 3',5'-cyclic phosphodiesterase 4B	1XOS/Q07343	Hydrolase	**-8.3±0.0***
3-hydroxy-3-methylglutaryl CoA synthase	1XPM/Q79ZY6	Transferase	-7.3±0.0
PIM-1 protein kinase	1XQZ/P11309	Transferase	-7.4±0.0
Glycerol kinase	1XUP/O34153	Transferase	**-8.2±0.0***
Uridine phosphorylase	1Y1T/P0A1F6	Transcription	-7.1±0.0
11-Hydroxysteroid dehydrogenase	1Y5M/P50172	Oxidoreductase	**-8.1±0.0***
Peptidyl dipeptidase Dcp	1Y79/P24171	Hydrolase	**-8.2±0.0***
Aspartate Aminotransferase	1YAA/P23542	Aminotransferase	-7.0±0.0
Heat shock protein 90	1YET/P07900	Chaperone Protein	-7.1±0.0
Yeast Glycogen Phosphorylase	1YGP/P06738	Glycosyltransferase	**-8.1±0.0***
Proto-oncogene tyrosine-protein kinase Src	1YOL/P12931	Transferase	-7.0±0.0
Inorganic Pyrophosphatase	1YPP/P00817	Hydrolase	-7.1±0.0
Serine carboxypeptidase	1YSC/P00729	Hydrolase(carboxypeptidase)	-7.2±0.0
falcipain-2	1YVB/Q9N6S8	Hydrolase/hydrolase Inhibitor	-6.4±0.0
Tyrosine-protein kinase JAK3	1YVJ/P52333	Transferase	-7.8±0.0
Carbonic anhydrase-III	1Z93/P07451	Lyase	-6.9±0.0
Enoyl-[acyl-carrier-protein] Reductase	1ZID/P9WGR1	Oxidoreductase	-7.7±0.0
Mannan-binding lectin serine protease-2	1ZJK/O00187	Hydrolase	-6.7±0.0
Histone lysine methyltransferase	1ZKK/Q9NQR1	Transferase	-7.9±0.0
Voltage gated potassium channel	1ZSX/Q13303	Oxidoreductase	**-9.0±0.0***
Ribonucleotide reductase	1ZZD/P21524	Oxidoreductase	-6.4±0.0
Aldehyde reductase II	1ZZE/Q9UUN9	Oxidoreductase	-7.4±0.0
Mineralocorticoid receptor	2A3I/P08235	Transferase	-7.3±0.0
AMP deaminase	2A3L/O80452	Hydrolase	**-8.6±0.0***
WASP interacting protein	2A3Z/P68135	Structural Protein	-6.8±0.0

WASP interacting protein	2A41/P68135	Structural Protein	-7.0±0.0
Endonuclease III	2ABK/P0AB83	Endonuclease	-6.7±0.0
MAP kinase-interacting serine/threonine kinase 2	2AC3/Q9HBH9	Transferase	-6.9±0.0
Acetylcholinesterase	2ACE/P04058	Serine Hydrolase	**-8.5±0.0***
Acetylcholinesterase	2ACK/P04058	Hydrolase	**-8.0±0.0***
Adenosine Deaminase	2ADA/P03958	Hydrolase	-7.9±0.0
Tropinone Reductase-II	2AE2/P50163	Oxidoreductase	**-9.2±0.0***
Arginase I	2AEB/P05089	Hydrolase/hydrolase Inhibitor	-7.6±0.0
Orotate phosphoribosyltransferase	2AEE/Q9A076	Transferase	-7.1±0.0
Adamalysin II	2AIG/P34179	Hydrolast/hydrolase Inhibitor	-7.3±0.0
Adenylate Kinase Isoenzyme-3	2AK3/P08760	Transferase (phosphotransferase)	-7.0±0.0
Alkaline phosphatase	2ANH/P00634	Alkaline Phosphatase	-6.8±0.0
Plasma kallikrein, light chain	2ANY/P03952	Blood Clotting	-7.1±0.0
Insulin Receptor	2AUH/P06213	Transferase/signaling Protein	-7.1±0.0
Ubiquitin carboxyl-terminal hydrolase 14	2AYO/P54578	Hydrolase	-7.2±0.0
Methionine aminopeptidase 1	2B3K/P53582	Hydrolase	**-8.0±0.0***
NAD-dependent deacetylase sirtuin-5	2B4Y/Q9NXA8	Hydrolase	-7.0±0.0
Jak2 tyrosine kinase	2B7A/O60674	Transferase	**-8.0±0.0***
Cholesterol esterase	2BCE/P30122	Hydrolase	-7.6±0.0
Serine/threonine-protein kinase/endoribonuclease IRE1	2BE1/P32361	Transcription	-7.9±0.0
Glycogen synthase	2BFW/Q9V2J8	Transferase	-6.2±0.0
Glucose-6-phosphate dehydrogenase	2BH9/P11413	Oxidoreductase	**-8.3±0.0***
Mannosyltransferase	2BO6/Q9RFR0	Transferase	**-8.0±0.0***
Checkpoint kinase 1	2BRO/O14757	Transferase	**-8.1±0.0***
Pyruvate dehydrogenase kinase-2	2BU5/Q15119	Transferase	**-8.0±0.0***
5-Aminolevulinate synthase	2BWN/P18079	Transferase	-7.1±0.0
GTP cyclohydrolase-II	2BZ1/P0A7I7	Hydrolase	-7.2±0.0
Beta-mannosidase	2C0H/Q8WPJ2	Hydrolase	-7.3±0.0
PAK-6 protein kinase	2C30/Q9NQU5	Transferase	-6.2±0.0
Casein kinase-I gamma-2	2C47/P78368	Transferase	-7.7±0.0
Glutamate carboxypeptidase II	2C6C/Q04609	Hydrolase	**-9.9±0.0***

GMP reductase 2	2C6Q/Q9P2T1	Oxidoreductase	**-8.5±0.0***
Superoxide dismutase	2C9V/P00441	Oxidoreductase	-6.4±0.0
Carbonic anhydrase form B	2CAB/P00915	Hydro Lyase	-7.2±0.0
Ornithine Aminotransferase	2CAN/P04181	Transferase	-7.5±0.0
Hyaluronidase	2CBI/Q0TR53	Hydrolase	-7.1±0.0
Other phosphate group transferase	2CG5/Q9NRN7	Transferase/hydrolase	-7.8±0.0
Chorismate Mutase	2CHT/P19080	Isomerase	-7.6±0.0
Sentrin specific protease-1	2CKG/Q9P0U3	Hydrolase	-6.5±0.0
Protein Tyrosine Phosphatase 1b	2CMB/P18031	Hydrolase	-7.1±0.0
malate dehydrogenase	2CMD/P61889	Oxidoreductase(nad(a) Choh(d))	-7.4±0.0
Arginine deiminase	2CMU/O24890	Hydrolase	-6.3±0.0
Cytochrome P450 Reductase	2CPP/P00183	Oxidoreductase(oxygenase)	**-8.1±0.0***
Carboxypeptidase A	2CTC/P00730	Hydrolase(c-Terminal Peptidase)	-6.8±0.0
ADP ribosylglycohydrolase	2CWC/Q5SMG9	Hydrolase	-6.7±0.0
Acyl CoA oxidase-2	2DDH/P07872	Oxidoreductase	**-8.6±0.0***
D-alanine:D-alanine ligase	2DLN/P07862	Ligase(peptidoglycan Synthesis)	-6.7±0.0
Endothiapepsin	2ER6/P11838	Hydrolase/hydrolase Inhibitor	-6.9±0.0
Endothiapepsin	2ER7/P11838	Hydrolase/hydrolase Inhibitor	-7.8±0.0
Ribonuclease HII	2ETJ/Q9X017	Hydrolase	-7.2±0.0
CLK3 protein kinase	2EU9/P49761	Transferase	-7.9±0.0
2-Dehydropantoate 2-reductase	2EW2/Q831Q5	Oxidoreductase	-7.0±0.0
Acetyl-CoA acetyltransferase, mitochondrial	2F2S/P24752	Transferase	-7.7±0.0
Serine/threonine-protein kinase PAK 7	2F57/Q9P286	Transferase	-6.3±0.0
Cytochrome P450 2D6	2F9Q/P10635	Oxidoreductase	**-8.0±0.0***
Ubiquitin thiolesterase-7	2FOJ/Q93009	Hydrolase	-6.2±0.0
Geranyltranstransferase	2FTZ/Q9WY08	Transferase	-7.5±0.0
Dipeptidyl peptidase 4	2G5T/P27487	Hydrolase	-7.5±0.0
Falcipain 2	2GHU/Q9N6S8	Hydrolase	-6.9±0.0
Hepatitis A Protease 3C	2HAL/P08617	Hydrolase/hydrolase Inhibitor	-6.9±0.0
Hematopoetic Cell Kinase HCK	2HCK/P08631	Transferase	-7.8±0.0
Dipeptidyl Peptiidase IV (DPP4)	2HHA/P27487	Hydrolase	-7.2±0.0

HIV-1 Integrase	2ITG/P12497	DNA Integration	-6.8±0.0
p450 3A4 (CYP,CYP450)	2J0D/P08684	Oxidoreductase	-7.4±0.0
Glutamine Synthetase	2LGS/P0A1P6	Ligase(amide Synthetase)	-7.4±0.0
Inosine-Uridine Nucleoside N-Ribohydrolase	2MAS/Q27546	Hydrolase	-6.5±0.0
UDP-N-Acetylmuramate dehydrogenase	2MBR/P08373	Oxidoreductase	**-9.0±0.0***
Peptidylprolyl isomerase	2NUL/P23869	Isomerase	-6.5±0.0
PARP	2PAW/P26446	Transferase	**-8.6±0.0***
P-Hydroxybenzoate Hydroxylase	2PHH/P00438	Oxidoreductase	**-8.6±0.0***
Human Plasminogen Kringle 4	2PK4/P00747	Hydrolase(serine Protease)	-5.8±0.0
Poliovirus (Type 1, Mahoney Strain)	2PLV/P03300	Virus	-7.6±0.0
Rhinovirus 14 (HRV14)	2R07/P03303	Virus	-7.1±0.0
Neuraminidase	2SIM/P29768	Hydrolase	-7.8±0.0
HIV-1 Protease	2UPJ/P03367	Hydrolase (acid Protease)	-7.9±0.0
Penicillopepsin	2WEA/P00798	Hydrolase	-6.9±0.0
Penicillopepsin	2WEC/P00798	Hydrolase	-7.5±0.0
Hexokinase B	2YHX/P04807	Transferase	-7.3±0.0
Triose Phosphate Isomerase	2YPI/P00942	Triose Phosphate Isomerase	-7.5±0.0
Carbonic anhydrase-IV	2ZNC/Q64444	Lyase	-7.0±0.0
Beta lactamase	3BLM/P00807	Hydrolase	-7.1±0.0
Alcohol Dehydrogenase	3BTO/P00327	Oxidoreductase	-7.4±0.0
Histone deacetylase 7a	3C0Z/Q8WUI4	Hydrolase	-7.1±0.0
Type III Chloramphenicol Acetyltransferase	3CLA/P00484	Transferase (acyltransferase)	-7.5±0.0
Carboxypeptidase A	3CPA/P00730	Hydrolase (c Terminal Peptidase)	-6.4±0.0
Dihydrofolate Reductase	3DFR/P00381	Oxido Reductase	-7.9±0.0
Endothiapepsin	3ER3/P11838	Hydrolase/hydrolase Inhibitor	**-8.0±0.0***
Gamma Chymotrypsin	3GCH/P00766	Hydrolase (serine Proteinase)	-7.3±0.0
Heat shock protein 70	3HSC/P19120	Hydrolase (acting On Acid Anhydrides)	-7.7±0.0
Reverse Transcriptase	3HVT/P03366	Nucleotidyltransferase	**-8.5±0.0***
Medium chain acyl CoA dehydrogenase	3MDE/P41367	Oxidoreductase	**-9.1±0.0***
Phosphoglycerate kinase	3PGK/P00560	Transferase	-7.3±0.0
Trypsin	3PTB/P00760	Hydrolase (serine Proteinase)	-6.6±0.0

Thermolysin	3TMN/P00800	Hydrolase/hydrolase Inhibitor	-7.9±0.0
Trypsin	3TPI/P00760	Hydrolase/hydrolase Inhibitor	-6.5±0.0
Carbonic Anhydrase IV	3ZNC/Q64444	Lyase	-7.4±0.0
Methanol Dehydrogenase	4AAH/P38539	Oxidoreductase (pqq(a) Choh(d))	-7.3±0.0
Human Fibroblast Collagenase	4AYK/P03956	Matrix Metalloproteinase	**-8.3±0.0***
Phospholipase A2	4BP2/P00593	Carboxylic Ester Hydrolase Zymogen	-7.4±0.0
Carbonic anhydrase-II	4CAC/P00918	Lyase(oxo Acid)	-7.0±0.0
Chymotrypsin	4CHA/P00766	Hydrolase (serine Proteinase)	-6.9±0.0
Citrate Synthase	4CTS/P00889	Oxo Acid Lyase	**-8.1±0.0***
Dihydrofolate Reductase	4DFR/P0ABQ4	Oxido Reductase	**-8.2±0.0***
Endothiapepsin	4ER1/P11838	Hydrolase/hydrolase Inhibitor	-7.8±0.0
Pancreatic Elastase	4EST/P00772	Hydrolase/hydrolase Inhibitor	-7.2±0.0
influenza virus hemagglutinin	4HMG/P03437	Viral Protein	-6.5±0.0
Phenylalanine Hydroxylase	4PAH/P00439	Monooxygenase	**-8.0±0.0***
HIV-1 Protease	4PHV/P12497	Hydrolase(aspartic Proteinase)	**-8.1±0.0***
Phenylalanine Hydroxylase	4THI/P45741	Transferase	-7.8±0.0
Serine Proteinase alpha-thrombin	4THN/P00734	Hydrolase/hydrolase Inhibitor	-6.7±0.0
Thermolysin	4TMN/P00800	Hydrolase/hydrolase Inhibitor	**-8.0±0.0***
tyrosyl-tRNA synthetase	4TS1/P00952	Ligase (synthetase)	-7.8±0.0
Protein (Ribonuclease T1)	5BU4/P00651	Hydrolase	-7.4±0.0
Cytochrome P450Cam	5CP4/P00183	Oxidoreductase	**-8.2±0.0***
Carboxypeptidase A	5CPA/P00730	Hydrolase (c-Terminal Peptidase)	-7.3±0.0
Cytochrome P450 Reductase	5CPP/P00183	Oxidoreductase(oxygenase)	-7.8±0.0
Endothiapepsin	5ER2/P11838	Hydrolase/hydrolase Inhibitor	-7.9±0.0
Phospholipase A2	5P2P/P00592	Hydrolase(carboxylic Ester)	**-8.4±0.0***
Beta Trypsin	5PTP/P00760	Serine Protease	-6.8±0.0
Thermolysin	5TLN/P00800	Hydrolase (metalloproteinase)	-7.9±0.0
Thermolysin	5TMN/P00800	Hydrolase/hydrolase Inhibitor	-7.9±0.0
Cyclooxygenase-2	6COX/Q05769	Oxidoreductase	**-9.3±0.0***
Carboxypeptidase A	6CPA/P00730	Hydrolase (c-Terminal Peptidase)	-7.1±0.0
Cytochrome P450 Reductase	6CPP/P00183	Oxidoreductase(oxygenase)	**-8.1±0.0***

ribonuclease T1	6RNT/P00651	Hydrolase(endoribonuclease)	-6.4±0.0
Ribonuclease A	6RSA/P61823	Hydrolase	-6.6±0.0
Thermolysin	6TMN/P00800	Hydrolase/hydrolase Inhibitor	-7.8±0.0
Aspartate Aminotransferase	7AAT/P00508	Transferase(aminotransferase)	-7.5±0.0
Carboxypeptidase A	7CPA/P00730	Hydrolase(c-Terminal Peptidase)	-6.6±0.0
Cytochrome P450 Reductase	7CPP/P00183	Oxidoreductase(oxygenase)	**-8.1±0.0***
Triosephosphate Isomerase (bacterial)	7TIM/P00942	Intramolecular Oxidoreductase	-7.3±0.0
HIV-1 Protease	7UPJ/P03367	Hydrolase	-7.0±0.0
Protein (Hydroxynitrile Lyase)	7YAS/P52704	Lyase	-5.9±0.0
Catalase	8CAT/P00432	Oxidoreductase	**-8.2±0.0***
Cytochrome P450 Reductase	8CPP/P00183	Oxidoreductase(oxygenase)	**-8.0±0.0***
Gamma-chymotrypsin	8GCH/P00766	Hydrolase/peptide	-6.6±0.0
Triose Phosphate isomerase	8TIM/P00940	Isomerase	-7.0±0.0
Aspartate Aminotransferase	9AAT/P00508	Transferase (aminotransferase)	-7.1±0.0
HIV-1 Protease	9HVP/P12497	Hydrolase/hydrolase Inhibitor	-7.5±0.0
Isocitrate dehydrogenase	9ICD/P08200	Oxidoreductase (nad(a) Choh(d))	-6.9±0.0

Table S14. Docking affinity scores for cis-dityrosine binding to circadian clock related proteins.

Target name	PDB/UniProtKB	Type	Affinity (kcal/mol)
CK II beta	1QF8/P67870	Transferase	-6.1±0.0
CKI-gamma 1	2CMW/Q9HCP0	Transferase	-7.5±0.0
CK II alpha'	2E3B/P19784	Oxidoreductase	**-8.0±0.0***
CKI-gamma 3	2IZR/Q9Y6M4	Transferase	-7.9±0.0
EAR-1R	2V0V/Q14995	Transcription	-6.5±0.0
CLK1	1Z57/P49759	Transferase	**-9.1±0.0***
CLK4	2W96/P24385	Transferase	**-8.0±0.0***
CLK3	2WU6/P49761	Transferase	-7.8±0.0
CK II alpha	3H30/P68400	Transferase	-7.7±0.0
CLK2	3NR9/P49760	Transferase	**-8.3±0.0***

Table S15. Docking affinity scores for cis-dityrosine binding to insulin receptor pathway proteins.

Target Name	PDB/UniProtKB	Type	Affinity (kcal/mol)
Leptin	1AX8/P41159	Cytokine	-6.0±0.0
Protein sinI	1B0N/P23308	Transcription regulator	-6.5±0.0
PTEN	1D5R/P60484	Hydrolase	-7.7±0.0
SOS 1	1DBH/Q07889	Gene regulation	-6.5±0.0
PI3K-gamma	1E8Y/P48736	Serine/threonine protein kinase	**-8.8±0.0***
TNF-R1	1EXT/P19438	Signaling protein	-6.3±0.0
CBL	1FBV/P22681	Ligase	-7.6±0.0
GAPDH	1GPD/P00357	Oxidoreductase	-7.3±0.0
NEDD4	1I5H/Q62940	Ligase	-6.3±0.0
Insulin Receptor	1IR3/P06213	Transferase	-7.4±0.0
CRK	1JU5/P46108	Protein binding/transferase	-6.6±0.0
GRB2	1JYR/P62993	Signaling protein	-5.7±0.0
Insulin	1MSO/P01308	Hormone/growth factor	-6.4±0.0
Glucagon	1NAU/P01275	Hormone/growth factor	-6.1±0.0
GRB10	1NRV/Q13322	Signaling protein/cytokine	-5.9±0.0
SHC1	1OY2/P29353	Signaling protein	-6.1±0.0
Interleukin 6	1P9M/P05231	Signaling protein/cytokine	-6.9±0.0
IRS-1	1QQG/P35568	Signaling protein	-6.8±0.0
c-RAF	1RFA/P04049	Serine/threonine protein kinase	-5.4±0.0
PP-1B	1S70/P62140	Hydrolase	-7.5±0.0
EIF2B4	1T5O/O29877	Translation	-7.5±0.0
NCK2	1U5S/O43639	Metal binding protein	-6.4±0.0
GSK3B	1UV5/P49841	Transferase	-7.9±0.0
Synip	1WI4/Q9WV89	Protein binding	-6.7±0.0

Flot2	1WIN/Q60634	Cell adhesion	-6.2±0.0
eIF-4E	1WKW/P06730	Translation/protein binding	**-8.1±0.0***
SOS-1	1XD4/Q07889	Signaling protein	-7.0±0.0
PKC-theta	1XJD/Q04759	Transferase	**-8.1±0.0***
RHEB	1XTS/Q15382	Signaling protein	-7.6±0.0
ASP	1Y7J/P42127	Signaling protein	-6.2±0.0
Zinc finger protein 174	1Y7Q/Q15697	Transcription	-7.4±0.0
p59-Fyn	1ZBJ/P06241	Transferase	-7.7±0.0
PPAR-gamma	1ZGY/P37231	Transcription	-7.9±0.0
TC10	2ATX/P17081	Hydrolase	-7.4±0.0
EHBP1	2D89/Q8NDI1	Structural protein , protein binding	-6.1±0.0
hSTP	2EFK/Q15642	Endocytosis/exocytosis	-6.3±0.0
ERK2	2ERK/P63086	Phosphotransferase	-6.6±0.0
EHD1	2JQ6/Q9H4M9	Metal binding protein	-6.6±0.0
FOXO3	2K86/O43524	DNA binding protein	-5.7±0.0
mTOR	2NPU/P42345	Transferase	-7.5±0.0
MAPK 10	2P33/P53779	Transferase	**-8.0±0.0***
MEK1	2P55/Q02750	Transferase	-7.8±0.0
PKA C-alpha	2QCS/P05132	Transferase/transferase inhibitor	**-8.9±0.0***
AMPK subunit beta-2	2V8Q/O43741	Transferase	-7.6±0.0
PDE10A	2WEY/Q9Y233	Hydrolase	**-8.0±0.0***
Phospholipase C	2WXT/Q0TV31	Hydrolase	-7.1±0.0
AKT-3	2X18/Q9Y243	Transferase	-6.2±0.0
AKT-2	2X39/P31751	Transferase	**-8.1±0.0***
P70S6K1	3A62/P23443	Transferase	-7.8±0.0
FOXO1	3CO6/Q12778	Transcription/DNA	**-8.3±0.0***
Hemoglobin	3DUT/P69905/P68871	Oxygen binding	-5.9±0.0
PTP1B	3EAX/P18031	Hydrolase	-6.8±0.0
EIF2BA	3ECS/Q14232	Translation	-7.9±0.0

IRS2	3FQX/Q9Y4H2	Immune system	-7.0±0.0
SGK1	3HDN/O00141	Transferase	-7.8±0.0
iNOS	3HR4/P35228	Oxidoreductase/metal Binding Protein	**-8.7±0.0***
hPDK1	3IOP/O15530	Transferase	-7.6±0.0
PKC-alpha	3IW4/P17252	Transferase	-7.9±0.0
EIF2BE	3JUI/Q13144	Translation	-6.0±0.0
FOXO4	3L2C/P98177	Transcription/DNA	-7.7±0.0
TNF-a	3L9J/P01375	Immune system	-6.4±0.0
ACL	3MWD/P53396	Transferase	-7.5±0.0
SHP-2	3O5X/Q06124	Hydrolase	-7.0±0.0

Table S16. Docking affinity scores for cis-dityrosine binding to serum proteins.

Target name	PDB/UniProtKB	Type	Affinity (kcal/mol)
Topo1	1A35/P11387	Isomerase/DNA	**-8.2±0.0***
TCP228	1B3A/P13501	Anti HIV protein	-6.1±0.0
MCAF	1DOK/P13500	Chemoattractant	-6.1±0.0
ALB	1E7A/P02768	Carrier protein	-7.4±0.0
PTP-1C	1FPR/P29350	Signaling protein	-7.0±0.0
MIP-4-alpha	1G2S/Q9Y258	Cytokine	-6.5±0.0
CKB-8	1G91/P55773	Cytokine	-6.4±0.0
LAG-1	1HUM/P13236	Cytokine(chemotactic)	-5.6±0.0
AE 1	1HYN/P02730	Membrane protein	**-8.1±0.0***
ApoC-II	1I5J/P02655	Lipid transport	-5.2±0.0
BPK	1K2P/Q06187	Transferase	-6.3±0.0
TCPTP	1L8K/P17706	Hydrolase	-6.8±0.0
PAI-1	1LJ5/P05121	Membrane protein	-6.4±0.0
HLA-B8	1M05/P30460	Immune system	-7.8±0.0
MIP-3-alpha	1M8A/P78556	Cytokine	-6.2±0.0
APOE	1NFN/P02649	Lipid transport	-6.2±0.0
C1 esterase	1NZI/P09871	Hydrolase	-6.2±0.0
B2M	1PY4/P61769	Signaling protein	-6.2±0.0
SL-2	1Q3A/P09238	Hydrolase	**-8.6±0.0***
HLA-A	1QVO/P13746	Immune system	-7.4±0.0
ADAM33	1R54/Q9BZ11	Hydrolase	-6.4±0.0
G-CSF	1RHG/P09919	Growth factor	-5.8±0.0
R-PTP-mu	1RPM/P28827	Receptor	-7.0±0.0
APP	1RW6/P05067	Cell adhesion	-6.4±0.0
HLA-A1	1W72/P30443	Immune system	-7.4±0.0
R-PTP-delta	1X5Z/P23468	Hydrolase	-5.8±0.0
MME	1Y93/P39900	Hydrolase	-7.0±0.0
L-CA	1YGR/P08575	Hydrolase	-7.2±0.0
HEPTP	1ZC0/P35236	Hydrolase	-6.4±0.0

R-PTP-R	2A8B/Q15256	Hydrolase	-6.5±0.0
TNF-a	2AZ5/P01375	Blood clotting	-7.2±0.0
PTP-H1	2B49/P26045	Hydrolase	-6.6±0.0
STEP	2BIJ/P54829	Hydrolase	-6.2±0.0
PTPN14	2BZL/Q15678	Hydrolase	-6.7±0.0
R-PTP-kappa	2C7S/Q15262	Hydrolase	-6.3±0.0
SSAO	2C10/Q16853	Oxidoreductase	-6.4±0.0
PTP-1B	2CM2/P18031	Hydrolase	-6.9±0.0
ADAM 17	2DDF/P78536	Hydrolase	**-8.1±0.0***
R-PTP-S	2FH7/Q13332	Hydrolase	-6.8±0.0
PTP-U2	2GJT/Q16827	Hydrolase	-6.6±0.0
GM-CSF	2GMF/P04141	Growth factor	-6.5±0.0
R-PTP-gamma	2H4V/P23470	Hydrolase	-7.1±0.0
R-PTP-beta	2HC1/P23467	Hydrolase	-6.9±0.0
MIP-2G	2HDL/O95715	Chemokine	-5.3±0.0
C1Q	2JG9/P02745/P02746/P02747	Membrane adhesion	-6.7±0.0
ADAM-TS 1	2JIH/Q9UHI8	Hydrolase	**-8.4±0.0***
R-PTP-epsilon	2JJD/P23469	Hydrolase	-7.4±0.0
MMP-20	2JSD/O60882	Hydrolase	-7.1±0.0
PTPN18	2OC3/Q99952	Hydrolase	-6.5±0.0
R-PTP-T	2OOQ/O14522	Hydrolase	-6.3±0.0
MEG2	2PA5/P43378	Hydrolase	-6.8±0.0
F8/G253 antigen	2PF6/P50895	Cell adhesion	-6.1±0.0
IAR	2QEP/Q92932	Hydrolase	-7.3±0.0
R-PTP-N	2QT7/Q16849	Hydrolase	-5.6±0.0
C1R	2QY0/P00736	Hydrolase	-7.9±0.0
ADAM-TS 4	2RJP/O75173	Hydrolase	-7.8±0.0
CCR5	2RLL/P51681	Membrane protein	-5.2±0.0
MEG	2VPH/P29074	Hydrolase	-6.5±0.0
HLE	2Z7F/P08246	Hydrolase/hydrolase Inhibitor	-6.2±0.0
PTP-1D	3B7O/Q06124	Hydrolase	-7.1±0.0

ADAM-TS 5	3B8Z/Q9UNA0	Hydrolase	**-8.3±0.0***
TIMP-3	3CKI/P3562z5	Hydrolase, hydrolase inhibitor	-7.3±0.0
Alpha-1-antitrypsin	3DRM/P01009	Hydrolase inhibitor	-7.6±0.0
ADORA2A	3EML/P29274	Membrane protein , receptor	**-8.3±0.0***
Cnt-a1	3FEH/O75689	Hydrolase activator	-6.7±0.0
MIP-1-alpha	3FPU/P10147	Immune system	-6.4±0.0
ADAM 22	3G5C/Q9P0K1	Membrane protein	-7.8±0.0
ADAM-TS 13	3GHM/Q76LX8	Hydrolase	-7.4±0.0
Galactohydrolase	3HG3/P06280	Hydrolase	-6.7±0.0
ALT2	3IHJ/Q8TD30	Transferase	-6.9±0.0
TopBP1	3JVE/Q92547	Protein binding	-6.1±0.0
R-PTP-zeta	3JXF/P23471	Cell adhesion	-6.9±0.0
AGP 1	3KQ0/P02763	Signaling protein	-7.5±0.0
MMP-13	830C/P45452	Matrix metalloprotease	**-8.2±0.0***

Table S17. Docking affinity scores for cis-dityrosine binding to breast cancer proteins.

Target name	PDB/UniProtKB	Type	Affinity (kcal/mol)
TIMP-2	1BR9/P16035	Hydrolase	-7.3±0.0
MMP-2	1CK7/P08253	Hydrolase	-7.5±0.0
BAX	1F16/Q07812	Apoptosis	-6.5±0.0
Smac	1FEW/Q9NR28	Apoptosis	-6.0±0.0
IFNG	1FYH/P01579	Immune system	-7.0±0.0
MMP-9	1GKC/P14780	Hydrolase	**-8.7±0.0***
PS2 protein	1HI7/P04155	Growth factor	-5.4±0.0
Stromelysin-1	1HY7/P08254	Hydrolase	**-8.2±0.0***
IL1RN	1IRP/P18510	Cytokine	-6.5±0.0
LAR	1LAR/P10586	Hydrolase	**-8.2±0.0***
VHL	1LM8/P40337	Transcription	-6.8±0.0
RAD51	1N0W/Q06609	Gene regulation/antitumor protein	-7.4±0.0
Caspase-9	1NW9/P55211	Apoptosis	-6.8±0.0
MMP-16	1RM8/P51512	Hydrolase	**-8.6±0.0***
BRIP1	1T29/Q9BX63	Signaling protein	-6.4±0.0
ER-alpha	1XPC/P03372	Nuclear receptor	-6.7±0.0
TOP2A	1ZXM/P11388	Isomerase	**-9.3±0.0***
AIF	2G2B/P55008	Immune system	-7.1±0.0
Protein FADD	2GF5/Q13158	Apoptosis	-7.5±0.0
DEP-1	2NZ6/Q12913	Hydrolase	-6.6±0.0
Bcl-2	2O21/P10415	Apoptosis	-7.2±0.0
RAIDD	2O71/P78560	Apoptosis	-5.8±0.0
LyP	2P6X/Q9Y2R2	Hydrolase	-7.6±0.0
CHEK2	2W0J/O96017	Transferase	**-8.4±0.0***
PALB2	2W18/Q86YC2	Nuclear protein	-7.5±0.0
MMP-14	3C7X/P50281	Hydrolase	-6.5±0.0
p53	3D06/P04637	Transcription	-6.4±0.0

Caspase-3	3H0E/P42574	Hydrolase	-7.1±0.0
Caspase-8	3H11/Q14790	Apoptosis	-7.2±0.0
Beta-G1	3HN3/P08236	Hydrolase	-7.4±0.0
Mcl-1	3KJ0/Q07820	Transferase	-6.9±0.0
RAR-alpha	3KMR/P10276	Transcription	-6.9±0.0
FAP-1	3LNX/Q12923	Signaling protein	-6.3±0.0
Beta-2 adrenoceptor	3NY8/P07550	Membrane protein	**-9.8±0.0***

Table S18. Docking affinity scores for trans-dityrosine binding to binding proteins.

Target name	PDB/UniProtKB	Type	Affinity (kcal/mol)
Galectin-1	1A78/P56217	Lectin	-6.4±0.0
Annexin III	1AXN/P12429	Calcium/phospholipid Binding Protein	**-8.1±0.0***
Calmodulin	1CTR/P62158	Calcium Binding Protein	-5.8±0.0
Seminal Plasma Protein Pdc-109	1H8P/P02784	Phosphorylcholine Binding Protein	-6.7±0.0
Annexin V	1HAK/P08758	Calcium/phospholipid Binding	-7.8±0.0
Alpha 1 antitrypsin	1HP7/P01009	Protein Binding	-7.6±0.0
Histidine-Binding Protein	1HSL/P0AEU0	Binding Protein	-6.6±0.0
Intestinal Fatty Acid Binding Protein	1ICN/P02693	Binding Protein(fatty Acid)	**-8.5±0.0***
Migration Inhibitory Factor-Related Protein 14	1IRJ/P06702	Metal Binding Protein	-7.1±0.0
Lysine-, Arginine-, Ornithine-Binding Protein	1LST/P02911	Amino Acid Binding Protein	-6.8±0.0
Streptavidin	1SRJ/P22629	Biotin Binding Protein	**-8.7±0.0***
Uteroglobin	1UTR/P17559	Mammalian Pcb Binding Protein	**-8.1±0.0***
Annexin A8	1W3W/P13928	Coagulation	-7.8±0.0
Annexin A2	1W7B/P07355	Calcium Binding Protein	-7.2±0.0
Insulin-like growth factor binding protein 1	1ZT3/P08833	Peptide Binding Protein	-6.1±0.0
D-Galactose D-Glucose Binding Protein	2GBP/P0AEE5	Periplasmic Binding Protein	-6.5±0.0
Intestinal Fatty Acid Binding Protein	2IFB/P02693	Fatty Acid Binding Protein	**-8.7±0.0***

Table S19. Docking affinity scores for trans-dityrosine binding to nuclear receptors.

Target name	PDB/UniProtKB	Type	Affinity (kcal/mol)
EAR-1	1A6Y/P20393	Transcription/DNA	**-8.6±0.0***
Cellular Retinoic-Acid-Binding Protein Type II	1CBS/P29373	Retinoic Acid Transport	**-8.1±0.0***
Nuclear Vitamin D Receptor	1DB1/P11473	Gene Regulation	**-8.4±0.0***
Lac Repressor	1EFA/P03023	Transcription/dna	**-8.9±0.0***
Epididymal Retinoic Acid-Binding Protein	1EPB/P06911	Retinoic Acid Binding Protein	-7.7±0.0
Retinoid X Receptor, Beta	1H9U/P28702	Nuclear Receptor	-6.3±0.0
Oestrogen Receptor beta	1HJ1/Q62986	Nuclear Receptor	-7.0±0.0

Vitamin D3 Receptor	1IE9/P11473	Gene Regulation	**-8.8±0.0***
Orphan nuclear receptor PXR	1ILH/O75469	Gene Regulation	-7.8±0.0
ERR-beta	1LO1/O95718	Hormone/growth factor receptor/DNA	**-8.0±0.0***
Hepatocyte nuclear factor 4-gamma	1LV2/Q14541	Transcription	-6.9±0.0
Nuclear receptor ROR-alpha	1N83/P35398	Lipid Binding Protein	**-8.4±0.0***
Thyroid hormone receptor Beta-1	1NAX/P10828	Membrane Protein	**-9.0±0.0***
Thyroid hormone receptor Alpha-1	1NAV/P10827	Membrane Protein	-7.5±0.0
Bile acid receptor	1OSH/Q96RI1	Transcription	-7.7±0.0
Bile acid receptor	1OSV/Q62735	DNA Binding Protein	-7.7±0.0
Orphan nuclear receptor NURR1	1OVL/P43354	Transcription	-6.3±0.0
Hepatocyte nuclear factor 4-alpha	1PZL/P41235	Transcription	-7.5±0.0
Estrogen Receptor beta	1QKM/Q92731	Nuclear Receptor	-6.5±0.0
Estradiol receptor	1QKT/P03372	Nuclear Receptor	-6.6±0.0
Plasma retinol-binding protein precursor	1RBP/P02753	Retinol Transport	-6.9±0.0
Transthyretin	1RLB/P02766	Complex (protein/protein)	-7.6±0.0
progesterone receptor	1SQN/P06401	Hormone/growth Factor Receptor	-7.6±0.0
Liver X receptor alpha	1UHL/P28702	DNA Binding Protein	**-8.0±0.0***
Retinoic acid receptor beta	1XAP/P10826	Transcription	**-8.1±0.0***
Estrogen receptor alpha	1XPC/P03372	Hormone/growth Factor Receptor	-6.8±0.0
Retinoic acid receptor RXR-alpha	1XVP/Q14994	DNA Binding Protein	-7.3±0.0
Orphan nuclear receptor NR5A2	1YUC/Q15466	Transcription Regulation	**-8.0±0.0***
Mineralocorticoid receptor	2AA2/P08235	Transcription	-7.5±0.0
Androgen receptor	2AM9/P10275	Hormone/growth Factor Receptor	-7.5±0.0
Peroxisome proliferator activated receptor delta	2AWH/Q03181	Gene Regulation	**-8.5±0.0***
Estrogen-related receptor gamma	2E2R/P62508	Transcription	-7.7±0.0
COUP transcription factor 1	2EBL/P10589	Transcription	-6.5±0.0
Peroxisome Proliferator Activated Receptor gamma	2F4B/P37231	Transcription Activator	**-8.1±0.0***
Retinoic acid receptor RXR-gamma	2GL8/P48443	Hormone/growth Factor Receptor	-6.8±0.0
Retinoic acid receptor gamma	2LBD/P13631	Nuclear Receptor	-6.5±0.0
Peroxisome proliferator-activated receptor alpha	2P54/Q07869	Transcription	-6.7±0.0

Orphan nuclear receptor NR4A1	2QW4/P22736	Hormone Receptor	-6.7±0.0
Orphan nuclear receptor NR1D2	2V0V/Q14995	Transcription	-6.8±0.0
Glucocorticoid receptor	3E7C/P04150	Transcription	-6.9±0.0
Estrogen Receptor alpha	3ERT/P03372	Nuclear Receptor	-6.9±0.0
Steroid hormone receptor ERR1	3K6P/P11474	Hormone Receptor	-6.3±0.0
Retinoic acid receptor RXR-alpha	3KWY/P19793	Transcription	-7.0±0.0
Nuclear receptor ROR-gamma	3L0L/P51449	Transcription	-7.7±0.0
Peroxisome proliferator-activated receptor gamma	3LMP/P37231	Transcription	-7.7±0.0

Table S20. Docking affinity scores for trans-dityrosine binding to transport proteins.

Target name	PDB/UniProtKB	Type	Affinity (kcal/mol)
Transforming protein RhoA	1A2B/P61586	Oncogene Protein	-7.6±0.0
Flavodoxin	1BU5/P00323	Electron Transport	**-8.2±0.0***
Cellular Retinoic-Acid-Binding Protein	1CBS/P29373	Retinoic Acid Transport	-7.9±0.0
Nonspecific lipid transfer protein	1CZ2/P24296	Lipid Binding Protein	**-8.4±0.0***
Retinol Binding Protein	1FEN/P18902	Transport Protein	**-8.1±0.0***
Clathrin Coat Assembly Protein Ap50	1HES/P84092	Endocytosis/exocytosis	-7.2±0.0
Cytochrome C2	1I8O/P00091	Electron Transport	-7.6±0.0
Plasma retinol-binding protein	1IIU/P41263	Transport Protein	**-8.5±0.0***
Neutrophil-Activating Protein A	1JI4/P43313	Metal Transport	-7.1±0.0
Lysine, Arginine, Ornithine-Binding Protein	1LAH/P02911	Amino Acid Transport	-6.7±0.0
Transforming protein RhoA	1OW3/Q07960	Gene Regulation/signaling Protein	-7.6±0.0
Potassium/Sodium Hyperpolarization-Activated	1Q3E/O88703	Transport Protein	-7.5±0.0
human retinol-binding protein	1QAB/P02753	Transport Protein	**-8.2±0.0***
Cystic fibrosis transmembrane regulator	1R0W/P26361	Transport Protein	-7.1±0.0
Hemoglobin	1SPG/P56251	Oxygen Transport	-7.0±0.0
Transferrin	1TFD/P19134	Iron Transport Protein	**-9.0±0.0***
Archaerhodopsin-1	1UAZ/P69051	Proton Transport	-6.3±0.0
Glutamate transporter	1XFH/O59010	Transport Protein	-7.0±0.0

Constitutive androstane alpha receptor	1XNX/O35627	Ligand Receptor/transcription Regulation	-7.4±0.0
Serum protein	2BX8/P02768	Transport Protein	**-8.1±0.0***
C-H-RAS P21 Protein	5P21/P01112	Oncogene Protein	-7.9±0.0

Table S21. Docking affinity scores for trans-dityrosine binding to receptors.

Target name	PDB/UniProtKB	Type	Affinity (kcal/mol)
Progesterone Receptor	1A28/P06401	Progesterone Receptor	**-8.0±0.0***
Tumor necrosis factor alpha	1A8M/P01375	Lymphokine	-6.8±0.0
Cytokine receptor common beta chain	1C8P/P32927	Membrane Protein	-6.2±0.0
Granulocyte Colony-stimulating Factor	1CD9/P40223	Cytokine	-6.4±0.0
Tumor Necrosis Factor Receptor Associated Protein 2	1D0A/Q12933	Apoptosis	-5.7±0.0
Elongation Factor Tu	1D2E/P49410	RNA Binding Protein	-7.5±0.0
Elongation Factor Tu	1D8T/Q7M0J8	Hydrolase/antibiotic	-6.8±0.0
Retinoid X Receptor-alpha	1DKF/P10276	Hormone/growth Factor Receptor	**-8.6±0.0***
Androgen Receptor	1E3G/P10275	Androgen Receptor	-6.6±0.0
Progesterone Receptor	1E3K/P06401	Human Progesterone Receptor	-7.5±0.0
High affinity immunoglobulin receptor alpha-subunit	1F2Q/P12319	Immune System	-6.6±0.0
Peroxisome Proliferator Activated Receptor gamma (PPAR gamma)	1FM6/P37231	Transcription	**-8.2±0.0***
Glutama receptor subunit 2	1FTK/P19491	Membrane Protein	**-8.1±0.0***
LIR-1	1G0X/Q8NHL6	Immune System	-6.8±0.0
Type I IL-1 receptor	1G0Y/P14778	Immune System	-7.5±0.0
androgen Receptor	1GS4/P10275	Androgen Receptor	**-8.9±0.0***
CD2	1HNF/P06729	T Lymphocyte Adhesion Glycoprotein	-5.8±0.0
Pyridoxine 5'-Phosphate Synthase	1HO4/P0A794	Biosynthetic Protein	-6.7±0.0
Peroxisome Proliferator Activated Receptor alpha (PPAR alpha)	1I7G/Q07869	Transcription	-6.8±0.0
Intercellular Adhesion Molecule-1	1IAM/P05362	Viral Protein Receptor	-6.4±0.0
Troponin C, Slow Skeletal and Cardiac Muscles	1IH0/P63316	Contractile Protein	-6.6±0.0

Herpesvirus entry mediator	1JMA/Q92956	Viral Protein	-7.1±0.0
Glucocorticoid-like receptor	1NHZ/P04150	Hormone Receptor	-7.4±0.0
Farnesoid X receptor	1OSH/Q96RI1	Transcription	-7.6±0.0
Farnesoid X receptor	1OSV/Q62735	DNA Binding Protein	-7.9±0.0
Metabotropic Glutamate Receptor 2	1P1N/P19491	Membrane Protein	**-9.8±0.0***
Glucocorticoid Receptor	1P93/P04150/Q155 96	Hormone Receptor	-7.7±0.0
Estrogen Receptor	1PCG/P03372	Transcription/inhibitor	-6.9±0.0
Phosphatidylinositol 3-kinase	1PIC/P27986	Complex (phosphotransferase/receptor)	**-8.4±0.0***
Histocompatibility Leukocyte Antigen (Hla)-Cw4 (Heavy Chain)	1QQD/P30504	Immune system	-7.9±0.0
Ecdysone Receptor	1R1K/Q7SIF6	Hormone/growth Factor Receptor	-7.1±0.0
CT610	1RCW/O84616	Oxidoreductase	-6.2±0.0
Glutamate receptor-6	1S50/P42260	Membrane Protein	-7.4±0.0
Troponin C slow skeletal-cardiac muscle	1SCV/P09860	Contractile Protein	-6.3±0.0
Ionotropic glutamate receptor	1TXF/P22756	Membrane Protein	-6.5±0.0
Retinoic acid receptor RXR-beta	1UHL/Q13133	DNA Binding Protein	**-8.3±0.0***
Oxysterols receptor LXR-beta	1UPV/P55055	Receptor	**-8.3±0.0***
Aryl hydrocarbon receptor	1X0O/P27540	Transcription	-6.3±0.0
Constitutive androstane alpha receptor	1XNX/O35627	Ligand Receptor/transcription Regulation	-7.6±0.0
Peroxisome Proliferator Activated Receptor delta(PPAR delta)	1Y0S/Q03181	Hormone/growth Factor Receptor	**-8.0±0.0***
Glutamate receptor-5	1YCJ/P22756	Membrane Protein	-6.4±0.0
Maltose-binding periplasmic protein	1YTV/P0AEX9	Sugar Binding Protein	**-8.6±0.0***
Troponin T	1YTZ/P12620	Contractile Protein	-7.0±0.0
Troponin T fast skeletal muscle	1YV0/P12620	Contractile Protein	-7.0±0.0
HLA class I histocompatibility antigen, B-27 alpha chain	2A83/P03989	Immune System	-7.4±0.0
Glutamate receptor-2	2AIX/P19491	Membrane Protein	-7.0±0.0
IL-4 receptor	2CYK/P05112	Cytokine	-6.3±0.0
D-Galactose D-Glucose Binding Protein	2GBP/P0AEE5	Periplasmic Binding Protein	-6.7±0.0
Interleukin-10	2ILK/P22301	Cytokine	-6.7±0.0
Hexokinase B	2YHX/P04807	Transferase	-7.5±0.0

Table S22. Docking affinity scores for trans-dityrosine binding to Monoclonal Antibodies.

Target name	PDB/UniProtKB	Type	Affinity (kcal/mol)
IGG1-KAPPA 59.1 FAB	1ACY/ P01869	Complex(antibody/hiv 1 Fragment)	-7.1±0.0

Anti-dinitrophenyl-spin-label monoclonal antibody Fab fragment	1BAF	Immune System	-7.3±0.0
Mucosal Addressin Cell Adhesion Molecule-1	1BQS/Q13477	Membrane Protein	-6.9±0.0
Fab' Fragment Of Monoclonal Antibody Db3	1DBM/P01868	Immunoglobulin	**-8.9±0.0***
Anti-Tgf alpha Antibody Fab-Fragment	1E4X/P01837	Immune System	-7.1±0.0
IGG2B-KAPPA 17E8 FAB	1EAP	Catalytic Antibody	**-8.2±0.0***
Histocompatibility	1EEY/P01892/P61769	Immune System	-6.9±0.0
Immunoglobulin E	1F2Q/P12319	Immune System	-7.0±0.0
LIR-1	1G0X/Q8NHL6	Immune System	-7.0±0.0
Type I IL-1 receptor	1G0Y/P14778	Immune System	**-8.0±0.0***
Pinch protein	1G47/P48059	Cell Adhesion	-6.5±0.0
CD81 Antigen	1G8Q/P60033	Immune System	-6.9±0.0
Human Class I Histocompatibility Antigen	1HHG/P01892	Histocompatibility Antigen	-7.6±0.0
Human Leukocyte Antigen Class II	1I4F/P01892	Immune System	-7.2±0.0
IGG2A-KAPPA 26-10 FAB	1IGJ	Immunoglobulin	-7.7±0.0
Immunoglobulin lambda Light Chain Dimer (Mcg)	1MCJ	Immunoglobulin	**-9.0±0.0***
MHC CLASS I H-2KB HEAVY CHAIN	1OSZ/P01901	Complex (mhc I/peptide)	-7.6±0.0
CD44 antigen	1POZ/P16070	Cell Adhesion	-6.5±0.0
Integrin alpha-1/beta-1	1QCY/P56199	Cell Adhesion	-6.7±0.0
Gonadotropin alpha Subunit	1QFW/P01215	Immune System	-7.5±0.0
Histocompatibility Leukocyte Antigen (Hla)-Cw4 (Heavy Chain)	1QQD/P30504	Immune System	-7.9±0.0
MGDF receptor	1V7M/P40225	Immune System/cytokine	-7.0±0.0
Mhc Class I H-2Kb Heavy Chain	1VAC/Q7SIF6	Complex (mhc I/peptide)	**-8.2±0.0***
Icam-2	1ZXQ/P13598	Cell Adhesion	-5.8±0.0
Igg2B Fab Fragment	2CGR/A2P1G9	Immunoglobulin	-6.7±0.0
IGG1-KAPPA DB3 FAB	2DBL/P01868	Immunoglobulin	-7.9±0.0
Immunoglobulin McPC603 Fab-Phosphocholine	2MCP/P01789	Immunoglobulin	-7.0±0.0
Cell adhesion molecule	2NCM/P13595	Cell Adhesion	-6.1±0.0
murine MHC class I H-2Kb	2VAA/P01901	Complex (mhc I/peptide)	-7.4±0.0
Mhc Class I H-2Kb Heavy Chain	2VAB/P01901	Complex (mhc I/peptide)	-7.5±0.0
4-4-20 (IgG2A) Fab Fragment	4FAB/P01865	Immunoglobulin	**-8.1±0.0***

Table S23. Docking affinity scores for trans-dityrosine binding to Factor, Regulator and Hormones.

Target name	PDB/UniProtKB	Type	Affinity (kcal/mol)
Tau protein	1B5L/P56828	Cytokine	-7.4±0.0
Microtubule Protein	1CZ7/P20480	Contractile Protein	-7.4±0.0
Coagulation Factor VIII Precursor	1D7P/P00451	Blood Clotting	-6.6±0.0
Coagulation Factor VIII Precursor	1DAR/Q5SHN5	Blood Clotting	-7.2±0.0
E-selectin	1G1T/P16581	Immune System	-5.8±0.0
Hepatocyte Growth Factor Receptor	1GMN/P14210	Hormone/growth Factor	-6.7±0.0
Growth Factor Bound Protein 2	1GRI/P62993	Signal Transduction Adaptor	-7.0±0.0
Elongation Factor Tu	1HA3/Q5SHN6	Translation	**-8.6±0.0***
Heparin-Binding Growth Factor 1	1HKN/P05230	Growth Factor	-6.1±0.0
Heat Shock 70 kDa protein 8	1HX1/P19120	Chaperone/chaperone Inhibitor	**-8.7±0.0***
Elongation Factor 1-alpha	1IJE/P02994	Translation	**-8.1±0.0***
Migration Inhibitory Factor-Related Protein 14	1IRJ/P06702	Metal Binding Protein	-7.3±0.0
Neutrophil-Activating Protein A	1JI4/P43313	Metal Transport	-7.2±0.0
Neurophysin 2	1JK4/P01180	Neuropeptide	-6.8±0.0
Imaginal Disc Growth Factor-2	1JND/Q9V3D4	Hormone/growth Factor	**-8.1±0.0***
Elongation Factor 1-alpha	1JNY/P35021	Translation	-7.5±0.0
Nuclear Orphan Receptor Lxr-beta	1K4W/P45446	Hormone/growth Factor	**-8.2±0.0***
Bone Morphogenetic Protein-7	1M4U/P18075	Hormone/growth Factor	-6.5±0.0
Glucocorticoid-like receptor	1NHZ/P04150	Hormone Receptor	-7.6±0.0
Glucocorticoid Receptor	1P93/P04150	Hormone Receptor	-7.6±0.0
Insulin	1Q4V/P01308	Hormone/growth Factor	-6.5±0.0
Gonadotropin alpha subunit	1QFW/P01215	Immune System	**-8.5±0.0***
FGF-4 receptor	1RML/P05230	Growth Factor	-6.9±0.0
Elongation Factor 1-alpha	1TUI/Q01698	Elongation Factor	-6.4±0.0
Insulin	1TYL/P01308	Hormone	-6.4±0.0
VEGF receptor	1VPP/P15692	Growth Factor/growth Factor Inhibitor	-6.4±0.0
Chaperonin	1VQ0/Q9X1B4	Chaperone	-6.5±0.0
deamino-oxytocin	1XY1/P01175	Hormone	-5.2±0.0
IGF binding protein-1	1ZT3/P08833	Peptide Binding Protein	-6.2±0.0
Heat shock protein 16.3	2BYU/Q41560	Chaperone	-7.0±0.0
Kinesin-like protein KIF11	2FKY/P52732	Cell Cycle	-7.3±0.0
Methylparaben Insulin	3MTH/P01315	Hormone	-6.3±0.0

Concanavalin A	5CNA/P02866	Lectin(agglutinin)	-6.5±0.0
Galectin-7	5GAL/P47929	Lectin	-6.1±0.0

Table S24. Docking affinity scores for trans-dityrosine binding to Structural Proteins.

Target name	PDB/UniProtKB	Type	Affinity (kcal/mol)
Yeast Hypothetical Protein	1CT5/P38197	Structural Genomics	-6.4±0.0
Ribosomal Protein S6 Kinase	1DMG/P38516	Gene Regulation	-6.0±0.0
Cardiac Troponin C	1DTL/P09860	Structural Protein	-7.7±0.0
ATP binding cassette	1F3O/Q58206	Structural Genomics	-7.6±0.0
HIV gp41 protein	1FAV/P03069	Viral Protein	-6.6±0.0
Pinch protein	1G47/P48059	Cell Adhesion	-6.4±0.0
Troponin C, Slow skeletal and cardiac muscles	1IH0/P63316	Contractile Protein	-6.6±0.0
Tubulin alpha chain	1JFF/P02550/P02554	Structural Protein	**-8.2±0.0***
CD44 antigen	1POZ/P16070	Cell Adhesion	-6.4±0.0
Integrin alpha-1/beta-1	1QCY/P56199	Cell Adhesion	-6.5±0.0
GP41 Envelope Protein	1QR8/Q76270	Viral Protein	-6.3±0.0
Troponin C, slow skeletal-cardiac muscle	1SCV/P09860	Contractile Protein	-6.5±0.0
Nucleotidyltransferase	1WOT/P83814	Structural Genomics	-6.3±0.0
Troponin T, fast skeletal muscle	1YV0/P12620	Contractile Protein	-7.0±0.0
Icam-2	1ZXQ/P13598	Cell Adhesion	-5.7±0.0
Myosin light chain kinase family	2BKH/Q29122	Motor Protein/metal Binding Protein	**-9.4±0.0***
Neural cell adhesion molecule	2NCM/P13595	Cell Adhesion	-6.1±0.0

Table S25. Docking affinity scores for trans-dityrosine binding to Signaling Proteins.

Target name	PDB/UniProtKB	Type	Affinity (kcal/mol)
Rho	1DS6/P52566	Signaling Protein	-7.7±0.0
Neutrophil Cytosol Factor 2	1E96/P19878	Signaling Protein	**-8.3±0.0***
Metabotropic Glutamate Receptor Subtype 1	1EWK/P23385	Signaling Protein	-7.3±0.0

Soluble tumor necrosis factor receptor 1	1FT4/P19438	Signaling Protein	-6.5±0.0
Rhodopsin	1HZX/P02699	Signaling Protein	-7.4±0.0
Na+ H+ ion exchange	1I92/O14745	Signaling Protein	-6.5±0.0
Metabotropic Glutamate Receptor Subtype 1	1ISS/P23385	Signaling Protein	-7.7±0.0
Ran-GPPNHP-RanBP1-RanGAP	1K5D/P62826 /P43487/P41391	Signaling Protein/signaling Activator	**-9.2±0.0***
Transforming protein RhoA	1KMQ/P61586	Signaling Protein	-7.7±0.0
Voltage-gated calcium channel beta2a subunit	1T0J/Q8VGC3	Signaling Protein	-7.1±0.0
Sonic hedgehog	1VHH/Q62226	Signaling Protein	-6.3±0.0
Signal Transducer and Activator Of Transcription 1-alpha/beta	1YVL/P42224	Signaling Protein	-6.6±0.0
Regulator of G-protein signaling 17	1ZV4/Q9UGC6	Signaling Protein	-6.3±0.0

Table S26. Docking affinity scores for trans-dityrosine binding to Ion Channels.

Target name	PDB/UniProtKB	Type	Affinity (kcal/mol)
Hypothetical Protein Slr1257	1II5/P73797	Membrane Protein	**-8.2±0.0***
Voltage-Gated Potassium Channel	1JVM/P0A334	Membrane Protein	**-8.5±0.0***
Chloride intracellular channel protein 4	2AHE/Q9Y696	Metal Transport	-6.5±0.0

Table S27. Docking affinity scores for trans-dityrosine binding to RNA.

Target name	PDB/UniProtKB	Type	Affinity (kcal/mol)
Elongation Factor TU	1D2E/P49410	RNA Binding Protein	-7.5±0.0
Elongation Factor TU	1D8T/P0CE47	Hydrolase/antibiotic	-6.8±0.0

Table S28. Docking affinity scores for trans-dityrosine binding to Lipid Binding Protein.

Target name	PDB/UniProtKB	Type	Affinity (kcal/mol)
Adipocyte Lipid-Binding Protein	1LIC/P04117	Lipid Binding Protein	-7.8±0.0
KES1 protein	1ZHY/P35844	Lipid Binding Protein	**-9.1±0.0***

Table S29. Docking affinity scores for trans-dityrosine binding to other undefined receptors.

Target name	PDB/UniProtKB	Type	Affinity (kcal/mol)
C-reactive protein	1B09/P02741	Immune System	-7.7±0.0
bilin binding protein (BBP)	1BBP/P09464	Bilin Binding	**-8.3±0.0***
Congerin I	1C1L/P26788	Sugar Binding Protein	-7.2±0.0
C-H-Ras P21 Protein	1CRP/P01112	Oncogene Protein	-7.4±0.0
GLGF-Domain Protein Homer	1DDV/Q9Z214	Signaling Protein	-6.5±0.0
Human Neutrophil Gelatinase	1DFV/P80188	Sugar Binding Protein	-7.3±0.0
Cholera Toxin B	1EEI/Q57193	Toxin	-7.6±0.0
Transthyretin	1ETA/P02766	Transport(thyroxine)	-6.1±0.0
Congerin II	1IS3/Q9YIC2	Sugar Binding Protein	-6.5±0.0
Catabolite Gene Activator Protein	1J59/P0ACJ8	Gene Regulation/dna	**-8.0±0.0***
Galectin-3	1KJL/P17931	Sugar Binding Protein	-6.4±0.0
Major Urinary Protein	1MUP/P02762	Pheromone Binding	-6.0±0.0
Holo-Neocarzinostatin	1NCO/P0A3R9	Antibacterial and Antitumor Protein	-6.9±0.0
Actin, alpha skeletal muscle	1NWK/P68135	Contractile Protein	**-8.7±0.0***
ROP Protein	1ROP/P03051	Transcription Regulation	-5.2±0.0
Galectin-2	1ULE/Q9P4R8	Sugar Binding Protein	-5.8±0.0
STAT Protein	1UUS/O00910	Signal Transduction	-7.4±0.0
Trp RNA-Binding Attenuation Protein	1WAP/P19466	RNA Binding Attenuation Protein	-6.3±0.0
Galectin	1WW7/Q6WY08	Sugar Binding Protein	-6.1±0.0

Table S30. Docking affinity scores for trans-dityrosine binding to other Enzymes.

Target name	PDB/UniProtKB	Type	Affinity (kcal/mol)
Calcium/calmodulin-dependent protein kinase	1A06/Q63450	Kinase	-6.9±0.0
Protein Kinase C, beta Type	1A25/P68403	Calcium Binding Protein	-6.6±0.0
Branched-Chain Amino Acid Aminotransferase	1A3G/P0AB80	Aminotransferase	-7.6±0.0
Adenosine Deaminase	1A4M/P03958	Hydrolase	-7.5±0.0

Alpha-thrombin	1A4W/P00734	Hydrolase/hydrolase Inhibitor	**-8.2±0.0***
Plasminogen Activator Inhibitor Type 1	1A7C/P05121	Hydrolase Inhibitor/peptide	-7.6±0.0
Protein Disulfide Oxidoreductase	1A8L/Q51760	Oxidoreductase	-6.9±0.0
Retroviral protease	1A94/P03367	Hydrolase/hydrolase Inhibitor	-7.6±0.0
HIV Protease	1AAQ/P03367	Hydrolase/hydrolase Inhibitor	-7.5±0.0
DNA gyrase	1AB4/P0AES4	Topoisomerase	-7.3±0.0
Abl tyrosine kinase	1ABO/P55194	Complex (kinase/peptide)	-5.9±0.0
Cytochrome Peroxidase	1AC4/P00431	Oxidoreductase	-7.9±0.0
Cytochrome Peroxidase	1AC8/P00431	Oxidoreductase	-7.7±0.0
Acetylcholinesterase	1ACJ/P04058	Hydrolase(carboxylic Esterase)	**-8.6±0.0***
Aspartate Carbamoyltransferase	1ACM/P0A786	Transferase	-7.9±0.0
Aconitate Hydratase	1ACO/P20004	Lyase(carbon Oxygen)	-7.4±0.0
Alcohol Dehydrogenase	1ADB/P00327	Oxidoreductase (nad(a) Choh(d))	-7.7±0.0
Alcohol Dehydrogenase	1ADC/P00327	Oxidoreductase (nad(a) Choh(d))	-7.5±0.0
Adenosine Deaminase	1ADD/P03958	Hydrolase(acting in Cyclicamidines)	-7.7±0.0
Alcohol Dehydrogenase	1ADF/P00327	Oxidoreductase(nad(a) Choh(d))	-7.2±0.0
Serine Proteinase alpha-thrombin	1AE8/P00734	Hydrolase/hydrolase Inhibitor	**-8.1±0.0***
Cytochrome C Peroxidase	1AEB/P00431	Oxidoreductase	-7.8±0.0
Actinidin	1AEC/P00785	Hydrolase	-7.1±0.0
Cytochrome C Peroxidase	1AEV/P00431	Oxidoreductase	**-8.1±0.0***
Aldehyde dehydrogenase	1AG8/P20000	Oxidoreductase	-7.6±0.0
Serine protease	1AH2/P27693	Serine Protease	-7.0±0.0
Phospholipase C	1AH7/P09598	Hydrolase	-7.7±0.0
Alpha-Momorcharin	1AHA/P16094	Glycosidase	-6.8±0.0
HIV-1 Protease	1AJV/P03366	Aspartyl Protease	-7.6±0.0
HIV-1 Protease	1AJX/P03366	Aspartyl Protease	-7.9±0.0
Glycolate Oxidase	1AL8/P05414	Flavoprotein	**-8.4±0.0***
Pepsin	1AM5/P56272	Aspartyl Protease	**-8.0±0.0***
Angiogenin	1ANG/P03950	Hydrolase (vascularization)	-6.2±0.0
cAMP-dependent Protein Kinase	1APM/P05132	Transferase(phosphotransferase)	-7.3±0.0
Penicillopepsin	1APV/P00798	Hydrolase/hydrolase Inhibitor	-7.8±0.0

Aspartate Aminotransferase	1ASE/P00509	Aminotransferase	-7.4±0.0
Atrolysin C	1ATL/P15167	Hydrolase/hydrolase Inhibitor	-7.1±0.0
Carboxylesterase	1AUR/Q53547	Hydrolase	-6.9±0.0
Carbonic Anhydrase II	1AVN/P00918	Lyase	-6.8±0.0
Human Fibroblast Collagenase	1AYK/P03956	Metalloprotease	-7.9±0.0
Phosphoenolpyruvate carboxykinase	1AYL/P22259	Kinase (transphosphorylating)	-7.7±0.0
Carbonic Anhydrase I	1AZM/P00915	Lyase(oxo Acid)	-7.6±0.0
Signal peptidase-I	1B12/P00803	Hydrolase	-6.9±0.0
TGF-beta receptor type I	1B6C/P36897	Complex (isomerase/protein Kinase)	**-8.4±0.0***
Haloalkane Dehalogenase	1B6G/P22643	Hydrolase	-6.4±0.0
Glutamate racemase	1B74/P56868	Isomerase	-7.8±0.0
malate dehydrogenase	1B8P/Q9ZF99	Oxidoreductase	-7.1±0.0
Glutamate Dehydrogenase	1BGV/P24295	Oxidoreductase	-7.3±0.0
17-beta Hydroxysteroid-Dehydrogenase Type 1,Estradiol 17 beta dehydrogenase	1BHS/P14061	Oxidoreductase	**-8.3±0.0***
Thymidylate Synthetase	1BID/P0A884	Methyltransferase	-7.4±0.0
Fructose biphosphatase	1BIF/P25114	Bifunctional Enzyme	**-9.5±0.0***
Nadph-Flavin Oxidoreductase	1BKJ/Q56691	Oxidoreductase	-7.3±0.0
beta-Lactamase	1BLH/P00807	Hydrolase(beta Lactamase)	-7.1±0.0
Chymotrypsin-like elastase family member 1	1BMA/P00772	Hydrolase/hydrolase Inhibitor	-7.1±0.0
Map Kinase P38	1BMK/Q16539	Transferase	**-8.5±0.0***
Serine Proteinase alpha-thrombin	1BMN/P00734	Hydrolase/hydrolase Inhibitor	**-8.1±0.0***
Interleukin-beta convertase	1BMQ/P29466	Hydrolase	-6.8±0.0
Methionine Synthase	1BMT/P13009	Methyltransferase	-7.9±0.0
Histone acetyltransferase	1BOB/Q12341	Acetyltransferase	-7.8±0.0
Cytosine DNA methyltransferase	1BOO/P11409	Transferase	**-8.5±0.0***
Dihydrofolate Reductase	1BOZ/P00374	Oxidoreductase	-7.9±0.0
Trypsin	1BRA/P00763	Proteinase/inhibitor	-7.1±0.0
Activin type-II receptor	1BTE/P27038	Transferase	-6.5±0.0
Liver Alcohol Dehydrogenase	1BTO/P00327	Oxidoreductase	**-8.3±0.0***

Delta 5-3-ketosteroid isomerase	1BUQ/P00947	Isomerase	-7.6±0.0
Protein (Cytochrome P450 BM-3)	1BVY/P14779	Oxidoreductase	**-8.0±0.0***
NADPH Dehydrogenase 1	1BWK/Q02899	Oxidoreductase	**-8.7±0.0***
Beta-Amylase	1BYB/P10538	Hydrolase(o Glycosyl)	**-8.5±0.0***
Dihydrofolate Reductase	1BZF/P00381	Oxidoreductase	**-8.2±0.0***
Carbonic Anhydrase I	1BZM/P00915	Lyase(oxo Acid)	-7.2±0.0
Neutrophil Collagenase	1BZS/P22894	Hydrolase	**-8.4±0.0***
Csdb Protein	1C0N/P77444	Lyase	-6.9±0.0
Ras-Binding Domain of the Serine/Threonine Kinase C-Raf1	1C1Y/P62834	Signaling Protein	**-8.8±0.0***
Glycinamide Ribonucleotide Formyltransferase	1C2T/P08179	Transferase	-7.5±0.0
Lumazine Synthase	1C41/Q9UVT8	Transferase	**-9.2±0.0***
Ornithine Decarboxylase	1C4K/P43099	Lyase	**-8.0±0.0***
Aspartate Aminotransferase, Cytoplasmic	1C9C/P00509	Transferase	**-8.3±0.0***
Fkbp-rapamycin Associated Protein	1C9H/P68106	Immune System	-6.3±0.0
Glutamate Mutase	1CB7/P80078	Isomerase	**-8.6±0.0***
Cobalt-Precorrin-4 Transmethylase	1CBF/O87696	Methyltransferase	**-8.5±0.0***
Carboxypeptidase A	1CBX/P00730	Hydrolase(c Terminal Peptidase)	-6.8±0.0
Arginase	1CEV/P53608	Hydrolase	-7.7±0.0
Cathepsin G	1CGH/P08311	Hydrolase/hydrolase Inhibitor	-7.0±0.0
n/a	1CGZ/P30074	Transferase	-7.0±0.0
Pnp Oxidase	1CI0/P38075	Oxidoreductase	**-8.2±0.0***
Protein (Triosephosphate Isomerase)	1CI1/P52270	Triosephosphate Isomerase	-7.3±0.0
Thymidylate Synthase	1CI7/P13100	Transferase	**-8.2±0.0***
Carbonic Anhydrase II	1CIL/P00918	Lyase(oxo Acid)	-7.8±0.0
Carbonic Anhydrase II	1CIM/P00918	Lyase(oxo Acid)	-7.2±0.0
Carbonic Anhydrase II	1CIN/P00918	Lyase(oxo Acid)	-7.2±0.0
Serine Hydroxymethyltransferase, Mitochondrial	1CJ0/P07511	Transferase	-6.8±0.0
MRNA Capping Enzyme	1CKN/Q84424	Capping Enzyme	**-8.4±0.0***
Cholesterol Esterase	1CLE/P32947	Lipase	-6.5±0.0

Nitrate reductase	1CNF/P17571	Oxidoreductase	-7.7±0.0
Carbonic Anhydrase II	1CNW/P00918	Lyase (oxo Acid)	-6.6±0.0
Carbonic Anhydrase II	1CNX/P00918	Lyase (oxo Acid)	-7.3±0.0
Carbonic AnhydraseII	1CNY/P00918	Lyase (oxo Acid)	-6.9±0.0
Cholesterol Oxidase	1COY/P22637	Oxidoreductase(oxygen Receptor)	**-8.9±0.0***
Aminopeptidase	1CP7/P80561	Hydrolase	-6.3±0.0
Carboxypeptidase A	1CPS/P00730	Hydrolase(c Terminal Peptidase)	-7.2±0.0
Cytochrome P450-Terp	1CPT/P33006	Oxidoreductase(oxygenase)	**-8.5±0.0***
Epoxide hydrolase	1CQZ/P34914	Hydrolase	**-8.6±0.0***
DNA Primase/Helicase	1CR1/P03692	Transferase	-7.7±0.0
Basic-type creatine kinase	1CRK/P11009	Transferase	-6.5±0.0
Cathepsin B	1CSB/P07858	Hydrolase/hydrolase Inhibitor	-7.0±0.0
Casein kinase I	1CSN/P40233	Phosphotransferase	**-8.0±0.0***
Cytidine Deaminase	1CTT/P0ABF6	Hydrolase	-7.2±0.0
Prostatic acid phosphatase	1CVI/P15309	Hydrolase	-7.5±0.0
Gingipain R	1CVR/P95493	Hydrolase/hydrolase Inhibitor	-6.2±0.0
Coagulation Factor VIIa	1CVW/P08709	Hydrolase/hydrolase Inhibitor	-7.1±0.0
Tryptophan Synthase	1CW2/P00929	Lyase	-7.7±0.0
Cyclooxygenase-2	1CX2/Q05769	Oxidoreductase	**-8.9±0.0***
DNA Topoisomerase I	1CY1/P06612	Isomerase	-7.6±0.0
DNA Topoisomerase I	1CY9/P06612	Isomerase	-6.3±0.0
Cyclophilin Receptor	1CYN/P23284	Isomerase/immunosuppressant	-6.7±0.0
Microtubule Motor Protein NCD	1CZ7/P20480	Contractile Protein	-7.7±0.0
Alcohol Dehydrogenase	1D1T/P40394	Oxidoreductase	-7.9±0.0
Maly Protein	1D2F/P23256	Transferase	-6.1±0.0
Acid phosphatase	1D2T/Q9S1A6	Hydrolase	-7.5±0.0
Myeloperoxidase	1D2V/P05164	Oxidoreductase	**-8.3±0.0***
Dihydroorotate Dehydrogenase, Mitochondrial	1D3G/Q02127	Oxidoreductase	-7.9±0.0
Serine Proteinase alpha-thrombin	1D4P/P00734	Hydrolase/hydrolase Inhibitor	**-8.2±0.0***
Pokeweed Antiviral Protein	1D6A/P10297	Hydrolase	-7.9±0.0
Topoisomerase III	1D6M/P14294	Isomerase	-7.8±0.0

Hypoxanthine-Guanine Phosphoribosyltransferase	1D6N/P00492	Transferase	**-9.3±0.0***
O-acetylserine Sulfhydrylase	1D6S/P0A1E3	Lyase	**-8.5±0.0***
Copper Amine Oxidase	1D6U/P46883	Oxidoreductase	-7.6±0.0
Coagulation Factor VIIa	1DAN/P08709	Hydrolase/hydrolase Inhibitor	-7.5±0.0
Cytosolic Phospholipase A2	1DB4/P14555	Hydrolase/hydrolase Inhibitor	**-8.0±0.0***
Deacetoxycephalosporin C Synthase	1DCS/P18548	Oxidoreductase	-7.1±0.0
Dihydrofolate Reductase	1DF7/P9WNX1	Oxidoreductase	**-8.7±0.0***
Peptide deformylase	1DFF/P0A6K3	Hydrolase	-6.9±0.0
Dialkylglycine decarboxylase	1DGD/P16932	Lyase	-7.0±0.0
Adenosine kinase	1DGM/Q9TVW2	Transferase	-7.5±0.0
Dihydrofolate Reductase	1DHF/P00374	Oxido Reductase	**-8.5±0.0***
Dihydrofolate Reductase	1DHJ/P0ABQ4	Oxidoreductase	**-8.9±0.0***
Estrogenic 17-Beta Hydroxysteroid Dehydrogenase	1DHT/P14061	Oxidoreductase	**-8.3±0.0***
Cyclin-Dependent Kinase 2 (Cdk2)	1DI8/P24941	Transferase	**-8.5±0.0***
Methylenetetrahydrofolate Reductase	1DIA/P11586	Oxidoreductase	-7.7±0.0
D-Xylose Isomerase	1DID/P12070	Isomerase(intramolecular Oxidoreductase)	**-8.1±0.0***
HIV-1 Protease	1DIF/P03367	Aspartic Proteinase	-7.7±0.0
Microbial Dihydrofolate Reductase	1DIH/P04036	Oxidoreductase	-7.5±0.0
Dihydrofolate Reductase	1DIS/P00381	Oxido Reductase	**-9.2±0.0***
Prostaglandin H2 Synthase-1	1DIY/P05979	Oxidoreductase	**-8.4±0.0***
8-Amino-7-Oxonanoate Synthase	1DJE/P12998	Transferase	-7.8±0.0
Ribosomal Protein L4	1DMG/P38516	Gene Regulation	-5.7±0.0
Catechol 1,2-Dioxygenase	1DMH/P07773	Oxidoreductase	-7.8±0.0
Phenylalanine Hydroxylase	1DMW/P00439	Oxidoreductase	**-8.3±0.0***
Atrial Natriuretic Peptide Receptor A	1DP4/P18910	Hormone/growth Factor Receptor	-7.9±0.0
Protein (Antigen 85-C)	1DQY/P9WQN9	Immune System	-7.7±0.0
Dihydrofolate Reductase	1DR1/P00378	Oxidoreductase	-7.9±0.0
Dihydrofolate Reductase	1DRF/P00374	Oxidoreductase (ch Nh(d) NAD or Nadp(a))	**-8.7±0.0***
Protein Kinase C, Alpha type	1DSY/P05696	Transferase	-7.5±0.0

Biotin Carboxylase,Acetyl CoA carboxylase	1DV1/P24182	Ligase	-7.9±0.0
Serine Proteinase alpha-thrombin	1DWB/P00734	Hydrolase/hydrolase Inhibitor	-7.4±0.0
Serine Proteinase alpha-thrombin	1DWC/P00734	Hydrolase/hydrolase Inhibitor	**-8.0±0.0***
Serine Proteinase alpha-thrombin	1DWD/P00734	Hydrolase/hydrolase Inhibitor	**-8.1±0.0***
Class II Chitinase	1DXJ/O81934	Hydrolase	-7.2±0.0
Nonstructural Protein NS2,Hepatitis C virus NS3 protease	1DXP/P26662	Hydrolase	-6.5±0.0
Nonstructural Protein NS3,Hepatitis C virus NS3 helicase	1DY9/P26662	Hydrolase/hydrolase Inhibitor	-6.8±0.0
beta-Glucosidase	1E1F/P49235	Hydrolase	**-9.9±0.0***
Thymidine Kinase	1E2K/P03176	Transferase	-6.5±0.0
Steroid Delta-Isomerase	1E3V/P07445	Isomerase	-7.3±0.0
Delta-Aminolevulinic Acid Dehydratase	1E51/P13716	Lyase	-6.0±0.0
beta-Glucosidase	1E55/P49235	Glycoside Hydrolase	**-9.2±0.0***
Methionine gamma-lyase	1E5F/O15564	Lyase	-6.6±0.0
GDP fucose synthetase	1E6U/P32055	Epimerase/reductase	**-8.3±0.0***
GDP-Fucose Synthetase	1E7S/P32055	Epimerase/reductase	**-8.4±0.0***
Pteridine Reductase	1E7W/Q01782	Oxidoreductase	**-8.2±0.0***
Cytochrome C	1E86/P00138	Electron Transport	-6.8±0.0
Enolase	1EBG/P00924	Carbon Oxygen Lyase	-6.9±0.0
Escherichia-Coli Chorismate Mutase	1ECM/P0A9J8	Chorismate Mutase	-6.6±0.0
Nitric Oxide Synthase	1ED5/P29473	Oxidoreductase	**-8.0±0.0***
Endothiapepsin	1EED/P11838	Hydrolase/hydrolase Inhibitor	-7.2±0.0
HIV-1 Reverse Transcriptase	1EET/P03366	Viral Protein	**-8.9±0.0***
Hydroxysteroid Sulfotransferase	1EFH/Q06520	Transferase	**-8.2±0.0***
Bovine Mitochondrial F1-ATPase	1EFR/P19483/P00829/P05631	Hydrolase/antibiotic	**-9.6±0.0***
Modification Methylase Rsri	1EG2/P14751	Transferase	-7.8±0.0
DNA Gyrase B Subunit	1EI1/P0AES6	Isomerase	**-8.7±0.0***
Lumazine Synthase	1EJB/P50861	Transferase	-7.5±0.0
Branched Chain Amino Acid-dependent Aminotransferase	1EKV/O15382	Transferase	-7.7±0.0
Elastase	1ELA/P00772	Hydrolase/hydrolase Inhibitor	-7.2±0.0

Elastase	1ELC/P00772	Hydrolase/hydrolase Inhibitor	-7.2±0.0
Porcine Pancreatic Elastase	1ELF/P00772	Complex (hydrolase/inhibitor)	-7.1±0.0
Endothiapepsin	1EPP/P11838	Hydrolase/hydrolase Inhibitor	-7.4±0.0
Prostaglandin H2 Synthase-1, COX-1	1EQH/P05979	Oxidoreductase	**-8.7±0.0***
Thio oxidoreductase	1ERU/P10599	Oxidoreductase	-5.3±0.0
BONE MORPHOGENETIC PROTEIN-2(bmp2)	1ES7/P12643	Cytokine	-7.1±0.0
Epsilon- Thrombin	1ETR/P00735	Hydrolase/hydrolase Inhibitor	-7.0±0.0
Epsilon- Thrombin	1ETS/P00735	Hydrolase/hydrolase Inhibitor	**-8.1±0.0***
Epsilon- Thrombin	1ETT/P00735	Hydrolase/hydrolase Inhibitor	-7.9±0.0
D-Amino acid oxidase	1EVI/P00371	Oxidoreductase	**-9.6±0.0***
Glycerol-3-phosphate dehydrogenase	1EVZ/P90551	Oxidoreductase	-7.7±0.0
Phospholipase D	1F0I/P84147	Hydrolase	-7.2±0.0
Cruzain	1F2A/P25779	Hydrolase	-6.8±0.0
PAK-1 protein kinase	1F3M/Q13153	Transferase	-7.1±0.0
Carboxypeptidase A	1F57/P00730	Hydrolase	-6.4±0.0
A-G adenine DNA glycAosylase	1F6O/P29372	Hydrolase/dna	-7.3±0.0
Lipoxygenas-1	1F8N/P08170	Oxidoreductase	**-8.4±0.0***
Coagulation Factor Xa	1FAX/P00742	Coagulation Factor	-7.8±0.0
Fibroblast Growth Factor Receptor 1	1FGK/P11362	Phosphotransferase	-7.1±0.0
Oxalate oxidase	1FI2/P45850	Oxidoreductase	-6.8±0.0
Factor XIII	1FIE/P00488	Transferase	**-8.4±0.0***
Beta-Acrosin	1FIW/Q9GL10	Hydrolase	-7.5±0.0
Carboxylase	1FIY/P00864	Complex (lyase/inhibitor)	**-9.4±0.0***
3-Alpha hydroxysteroid dehydrogenase	1FJH/P80702	Oxidoreductase	-7.5±0.0
Fk506 Binding Protein (Fkbp)	1FKF/P62942	Isomerase	-6.7±0.0
Fk506 Binding Protein (Fkbp)	1FKG/P62942	Cis Trans Isomerase	-6.8±0.0
Fk506 Binding Protein (Fkbp)	1FKI/P62942	Cis Trans Isomerase	-6.6±0.0
Memapsin 2 (Beta-Secretase)	1FKN/P56817	Hydrolase/hydrolase Inhibitor	**-8.2±0.0***
Peptidase T	1FNO/P26311	Hydrolase	**-8.3±0.0***
Xanthine dehydrogenase	1FO4/P80457	Oxidoreductase	**-8.0±0.0***

Fructose-1,6-bisphosphatase	1FRP/P00636	Hydrolase(phosphoric Monoester)	-6.7±0.0
Protein farnesyl transferase	1FT2/Q04631	Transferase	-7.7±0.0
Methionine sulfoxide reductase	1FVG/P54149	Oxidoreductase	-6.6±0.0
Adenylate cyclase	1FX2/Q99279	Lyase	-6.8±0.0
Glucose phosphate thymidylyltransferase	1FXO/Q9HU22	Transferase	**-8.7±0.0***
Phosphotransferase	1FYN/P06241	Transferase	-5.9±0.0
Metalloendopeptidase	1G12/P81054	Hydrolase	-7.9±0.0
Glucosidase-471	1GAI/ P69327	Hydrolase	**-8.0±0.0***
Macrophage Migration Inhibitory Factor	1GCZ/P14174	Immune System	-6.9±0.0
Aspartate Aminotransferase	1GD9/O59096	Transferase	-7.1±0.0
Protein Tyrosine Phosphatase 1b	1GFY/P18031	Hydrolase	-6.9±0.0
Gamma Chymotrypsin	1GHB/P00766	Hydrolase/hydrolase Inhibitor	-6.9±0.0
Cell Division Protein Kinase 2(CDK2)	1GIH/P24941	Transferase	**-9.0±0.0***
Cyclin Dependent Kinase 2(Cdk2)	1GII/P24941	Transferase	**-8.0±0.0***
Aspartate-semialdehyde Dehydrogenase	1GL3/P0A9Q9	Oxidoreductase	-7.6±0.0
Tec tyrosine kinase	1GL5/P24604	Transferase	-6.2±0.0
Glutathione S-transferase	1GLP/P19157	Transferase(glutathione)	-6.6±0.0
Glutathione S-transferase	1GLQ/P19157	Transferase(glutathione)	-6.8±0.0
Monoamine Oxidase	1GOS/P27338	Oxidoreductase	**-9.4±0.0***
Spinach glycolate oxidase	1GOX/P05414	Oxidoreductase (oxygen(a))	**-9.3±0.0***
Leucoanthocyanidin Dioxygenase	1GP6/Q96323	Oxidoreductase	-7.5±0.0
Glycogen Phosphorylase B	1GPB/P00489	Glycogen Phosphorylase	**-8.5±0.0***
Acetylcholinesterase	1GPK/P04058	Hydrolase	**-8.2±0.0***
GMP synthetase	1GPM/P04079	Transferase (glutamine Amidotransferase)	**-8.6±0.0***
Acetylcholinesterase	1GPN/P04058	Hydrolase	**-9.1±0.0***
Transketolase 1	1GPU/P23254	Transferase	-7.7±0.0
Acetylcholinesterase	1GQS/P04058	Hydrolase	-7.8±0.0
Glutathione reductase	1GRE/P00390	Oxidoreductase	**-8.2±0.0***
Glutathione Transferase A1-1	1GSF/P08263	Transferase (glutathione)	**-8.8±0.0***
Thymidine Kinase	1GSI/P9WKE1	Transferase	**-8.9±0.0***

Glutathione S-transferase	1GTB/P08515	Glutathione Transferase	-6.2±0.0
Cyclin Dependent Kinase 2(Cdk2)	1GZ8/P24941	Transferase	**-8.0±0.0***
Leukocyte Elastase	1H1B/P08246	Hydrolase	-6.8±0.0
Nitrogenase Molybdenum Iron Protein Alpha Chain	1H1L/P00466	Oxidoreductase	**-8.3±0.0***
CDK2	1H28/P24941	Cell Cycle/transferase Substrate	-7.7±0.0
Sulfurtransferase	1H4K/P52197	Transferase	-6.7±0.0
Glycogen phosphorylase	1H5U/P00489	Glycogen Metabolism	**-8.5±0.0***
Dihydropyrimidine dehydrogenase	1H7X/Q28943	Electron Transfer	**-8.3±0.0***
Polyamine oxidase	1H82/O64411	Oxidoreductase	**-8.4±0.0***
Serine Proteinase alpha-thrombin	1H8D/P00734	Hydrolase/hydrolase Inhibitor	-7.5±0.0
Proto-Oncogene Tyrosine-Protein Kinase LCK	1H92/P06239	Transferase	-6.8±0.0
Phosphatidylinositol 3-kinase	1H9O/P27986	Transferase/receptor	-6.0±0.0
Elongation Factor Tu	1HA3/Q5SHN6	Translation	**-8.6±0.0***
Isopenicillin N Synthase	1HB2/P05326	Antibiotic Biosynthesis	**-8.2±0.0***
HIV-1 Protease	1HBV/P03366	Hydrolase (acid Protease)	-7.8±0.0
3-alpha-hydroxysteroid Dehydrogenase	1HDC/P19992	Oxidoreductase	**-8.1±0.0***
Galectin-10	1HDK/Q05315	Serine Esterase	-6.3±0.0
Biliverdin Ix Beta Reductase	1HDO/P30043	Biliverdin Ix Beta Reductase	**-8.0±0.0***
Serine Proteinase alpha-thrombin	1HDT/P00734	Hydrolase/hydrolase Inhibitor	**-8.3±0.0***
Alcohol Dehydrogenase	1HDY/P00325	Oxidoreductase(nad(a) Choh(d))	-7.7±0.0
HIV-1 Protease	1HEF/P03366	Hydrolase/hydrolase Inhibitor	-6.3±0.0
Fibroblast Collagenase	1HFC/P03956	Metalloprotease	-7.7±0.0
L-Asparaginase	1HFW/P06608	Asparaginase	-6.3±0.0
Hypoxanthine-guanine-xanthine Phosphoribosyltransferase	1HGX/P51900	Transferase (glycosyltransferase)	-7.8±0.0
Human Class I Histocompatibility Antigen	1HHJ/P01892	Histocompatibility Antigen	-7.9±0.0
HIV-1 Protease	1HIH/P03366	Hydrolase (aspartic Proteinase)	-7.4±0.0
Diaminopimelate Decarboxylase	1HKV/P9WIU7	Lyase	-7.9±0.0
Fucosidase alpha	1HL9/Q9WYE2	Hydrolase	-7.1±0.0
Alcohol Dehydrogenase	1HLD/P00327	Oxidoreductase(ch Oh(d) Nad(a))	**-8.2±0.0***
Hypoxanthine-Guanine Phosphoribosyltransferase	1HMP/P00492	Transferase (glycosyltransferase)	-6.6±0.0

Prophospholipase A2	1HN4/P00592	Hydrolase	**-9.0±0.0***
Glutathione S-transferase	1HNA/P28161	Transferase (glutathione)	-6.4±0.0
Human Neutrophil Elastase	1HNE/P08246	Hydrolase/hydrolase Inhibitor	-5.9±0.0
HIV-1 Reverse Transcriptase	1HNI/P03366	Nucleotidyltransferase	-7.8±0.0
Phenylethanolamine N-Methyltransferase	1HNN/P11086	Transferase	-7.0±0.0
Pyridoxine 5'-Phosphate Synthase	1HO4/P0A794	Biosynthetic Protein	**-8.1±0.0***
HIV-1 Protease	1HOS/P03366	Hydrolase(acid Proteinase)	-7.6±0.0
Beta-N-acetylhexosaminidase	1HP5/O85361	Hydrolase	-7.7±0.0
Glutamic Acid-Specific Protease	1HPG/Q07006	Hydrolase/hydrolase Inhibitor	-6.4±0.0
Adenosinetriphosphatase	1HPM/P19120	Hydrolase (acting On Acid Anhydrides)	**-9.5±0.0***
HIV-1 Protease	1HPS/P03366	Hydrolase(acid Proteinase)	**-8.2±0.0***
Human Rhinovirus 14	1HRI/P03303	Virus	-7.3±0.0
Leukotriene A4 hydrolase	1HS6/P09960	Hydrolase	**-8.8±0.0***
Prostaglandin G/H Synthase 1, COX-1	1HT8/P05979	Oxidoreductase	-7.5±0.0
UDP-n-acetylglucosamine Pyrophosphorylase	1HV9/P0ACC7	Transferase	-7.5±0.0
D-alanyl-D-alanine Carboxypeptidase	1HVB/P15555	Hydrolase	**-8.1±0.0***
Estrogen Sulfotransferase	1HY3/P49888	Transferase	-7.0±0.0
Thermolysin	1HYT/P00800	Hydrolase(metalloproteinase)	-7.3±0.0
Riboflavin Synthase	1HZE/P0AFU8	Transferase	-6.4±0.0
Riboflavin alpha synthase	1I18/P0AFU8	Transferase	-7.4±0.0
Precorrin-8X Methylmutase	1I1H/P21638	Isomerase	-6.4±0.0
4-Amino-4-Deoxychorismate Lyase	1I2K/P28305	Lyase	-7.3±0.0
Insulin Receptor	1I44/P06213	Transferase	-7.7±0.0
Carbonic anhydrase	1I6O/P61517	Lyase	-6.9±0.0
DNA-directed RNA Polymerase II 19 KDa Polypeptide	1I6V/Q9KWU8	Transcription	-7.6±0.0
Apolipoprotein(a)	1I71/P08519	Hydrolase	-5.6±0.0
Neutrophil Collagenase, MMP8	1I76/P22894	Hydrolase	**-8.6±0.0***
Methionine decarboxylase	1I7B/P17707	Lyase	**-9.3±0.0***
Riboflavin Synthase	1I8D/P0AFU8	Transferase	**-8.3±0.0***

Glutamate Mutase	1I9C/P80078	Isomerase	-7.5±0.0
Phosphatidylinositol phosphatase	1I9Z/O43001	Hydrolase	-6.8±0.0
Dihydrofolate Reductase	1IA1/P22906	Oxidoreductase	-7.8±0.0
Transient Receptor Potential-Related Protein,Atypical protein kinase	1IA9/Q923J1	Transferase	-7.4±0.0
1-Aminocyclopropane-1-Carboxylate Synthase 2	1IAY/P18485	Lyase	-7.4±0.0
14-3-3 Zeta: Serotonin	1IB1/P63104	Signaling Protein/transferase	-7.6±0.0
Cystathionine beta-lyase	1IBJ/P53780	Lyase	-7.7±0.0
Interleukin-1 Beta Converting Enzyme	1ICE/P29466	Hydrolase/hydrolase Inhibitor	-6.4±0.0
12-Oxophytodienoate Reductase 1	1ICP/Q9XG54	Oxidoreductase	-7.8±0.0
Nitroreductase	1ICR/P38489	Oxidoreductase	**-8.1±0.0***
HIV-2 Protease	1IDA/P04584	Hydrolase/hydrolase Inhibitor	-7.7±0.0
NH(3)-dependent NAD(+) Synthetase	1IFX/P08164	Ligase	-7.8±0.0
Thiamin Pyrophosphokinase	1IG0/P35202	Transferase	**-8.2±0.0***
Thiamin Pyrophosphokinase	1IG3/Q9R0M5	Transferase	**-8.7±0.0***
Cyclophilin 40	1IHG/P26882	Isomerase	-6.7±0.0
InaD	1IHJ/Q24008	Signaling Protein	-6.5±0.0
Peptide N-myristoyltransferase	1IIC/P14743	Transferase	-7.9±0.0
Cholesterol Oxidase	1IJH/P12676	Oxidoreductase	**-9.1±0.0***
Histidinol Phosphate Aminotransferase	1IJI/P06986	Transferase	-7.7±0.0
Pol Polyprotein	1IKX/P03366	Transferase	-7.8±0.0
Inositol monophosphatase	1IMB/P29218	Hydrolase	**-8.0±0.0***
Holliday Junction DNA Helicase Ruvb	1IN4/Q56313	DNA Binding Protein	-7.6±0.0
Influenza A Subtype N2 Neuraminidase	1ING/P06820	Hydrolase (o Glycosyl)	-7.2±0.0
Dipeptidase	1ITU/P16444	Hydrolase	-6.5±0.0
Phosphotransketolase	1ITZ/Q7SIC9	Transferase	-7.8±0.0
Microbial Transglutaminase	1IU4/P81453	Transferase	-7.7±0.0
Influenza A Subtype N2 Neuraminidase	1IVD/P06820	Hydrolase (o Glycosyl)	-7.6±0.0
Neuraminidase	1IVE/P06820	Hydrolase (o Glycosyl)	-7.2±0.0

Aspartate Aminotransferase, Cytoplasmic	1IX6/P00509	Transferase	-7.5±0.0
Branched Chain Amino Acid-dependent Aminotransferase	1IYD/P0AB80	Transferase	**-8.7±0.0***
Hematopoietic Prostagladin D Synthase	1IYH/O60760	Isomerase	-7.9±0.0
Oxygenase	1J02/P06762	Oxidoreductase	-6.9±0.0
1-Aminocyclopropane-1-Carboxylate Deaminase	1J0E/Q7M523	Lyase	-6.9±0.0
Glycogen synthase kinase-3 beta	1J1B/P49841	Transferase	-7.8±0.0
Aspartate Aminotransferase, Cytoplasmic	1J32/Q8RR70	Transferase	-7.2±0.0
cAMP-dependent protein kinase catalytic subunit	1J3H/P05132	Transferase	-7.1±0.0
Fk506-binding Protein 1a	1J4R/P62942	Isomerase	-6.4±0.0
Heme oxygenase	1J77/Q9RGD9	Oxidoreductase	-7.3±0.0
Deoxyribonucleoside Kinase	1J90/Q9XZT6	Transferase	-7.0±0.0
Aldo-Keto Reductase Family 1 Member C3	1J96/P52895	Oxidoreductase	**-8.8±0.0***
Alcohol Sulfotransferase	1J99/Q06520	Transferase	-7.9±0.0
Metalloprotease 8	1JAP/P22894	Complex (metalloprotease/inhibitor)	-7.9±0.0
CAMP-DEPENDENT PROTEIN KINASE, ALPHA-CATALYTIC SUBUNIT	1JBP/P05132	Transferase	**-9.2±0.0***
Cystathionine beta-synthase	1JBQ/P35520	Lyase	-7.9±0.0
IMP-1 dehydrogenase	1JCN/P20839	Oxidoreductase	-7.7±0.0
L-Isoaspartyl D-aspartyl O-methyltransferase	1JG1/Q8TZR3	Transferase	-5.9±0.0
Amylosucrase	1JGI/Q9ZEU2	Transferase	-7.5±0.0
Cyclic nucleotide phosphodiesterase	1JH7/O04147	Hydrolase	**-8.3±0.0***
Neutrophil-Activating Protein A	1JI4/P43313	Metal Transport	-7.2±0.0
Porcine Pancreatic Elastase	1JIM/P00772	Hydrolase(serine Proteinase)	-7.2±0.0
Phenylalanyl-tRNA Synthetase	1JJC/Q5SGX2	Ligase	-7.7±0.0
HIV Protease	1JKH/P04585	Transferase	**-8.4±0.0***
Death-Associated Protein Kinase	1JKL/P53355	Transferase	-7.2±0.0
Glycinamide Ribonucleotide Formyltransferase	1JKX/P08179	Transferase	-7.1±0.0
cAMP-dependent Protein Kinase	1JLU/P05132	Transferase/transferase Inhibitor	**-8.1±0.0***
Jun N terminal kinase	1JNK/P53779	Transferase	**-8.4±0.0***

Pyridoxine 5'-Phosphate Oxidase	1JNW/P0AFI7	Oxidoreductase	-6.8±0.0
NADH Peroxidase	1JOA/P37062	Oxidoreductase	**-8.0±0.0***
Dihydrofolate Reductase	1JOM/P0ABQ4	Oxidoreductase	-7.3±0.0
Methylthioadenosine phosphorylase	1JP7/P50389	Transferase	**-8.1±0.0***
Phospholipase A2	1JQ9/P59071	Hydrolase/hydrolase Inhibitor	**-8.1±0.0***
Histamine N-methyltransferase	1JQE/P50135	Transferase	**-9.2±0.0***
Insulin-like Growth Factor I Receptor	1JQH/P08069	Transferase	-7.1±0.0
Inosine-5'-monophosphate Dehydrogenase 2,IMP-2 dehydrogenase	1JR1/P12269	Oxidoreductase	**-8.6±0.0***
Catechol O-Methyltransferase	1JR4/P22734	Transferase	-6.6±0.0
Aromatic-L-amino-acid Decarboxylase	1JS3/P80041	Lyase	**-8.3±0.0***
Dopa decarboxylase	1JS6/P80041	Lyase	**-8.7±0.0***
Hydroxysteroid-Dehydrogenase Type 1	1JTV/P14061	Oxidoreductase	-7.5±0.0
Dihydroorotate Dehydrogenase	1JUE/A2RJT9	Oxidoreductase	-7.7±0.0
DXP reductoisomerase	1JVS/P45568	Oxidoreductase	-7.2±0.0
Casein Kinase II, Alpha Chain	1JWH/P68400	Transferase	**-8.1±0.0***
Prothrombin	1JWT/P00734	Hydrolase	**-8.3±0.0***
Methylthioadenosine nucleosidase,S-Adenosylhomocysteine nucleosidase	1JYS/P0AF12	Hydrolase	-6.7±0.0
NADPH Dehydrogenase	1K02/Q02899	Oxidoreductase	-6.6±0.0
Tryptophan Synthase Alpha chain	1K3U/P00929	Lyase	-7.6±0.0
Glutathione Transferase A1-1	1K3Y/P08263	Transferase	**-8.7±0.0***
Nicotinate-nucleotide Adenylyltransferase	1K4M/P0A752	Transferase	**-8.2±0.0***
Cytosine deaminase	1K6W/P25524	Hydrolase	**-8.2±0.0***
5-methyltetrahydrofolate--homocysteine Methyltransferase,Methionine Synthase	1K7Y/P13009	Transferase	-7.7±0.0
Caspase-7	1K86/P55210	Apoptosis	-6.9±0.0
Triacylglycerol Lipase, Pancreatic	1K8Q/P80035	Hydrolase	**-8.1±0.0***
Transforming Protein P21/H-Ras-1	1K8R/P01112	Signaling Protein	**-8.8±0.0***

Histidinol Dehydrogenase	1KAE/P06988	Oxidoreductase	**-8.3±0.0***
Neutrophil Collagenase	1KBC/P22894	Metalloproteinase	**-8.8±0.0***
Thymidine Kinase	1KIM/P03176	Transferase	-6.9±0.0
Urease	1KRA/P18316	Hydrolase (urea Amido)	-7.3±0.0
Alpha-N-acetylgalactosaminidase	1KTB/Q90744	Hydrolase	-6.5±0.0
Tgf-beta Type II Receptor	1KTZ/P10600	Cytokine/cytokine Receptor	-6.0±0.0
Mycolic Acid Synthase	1L1E/P9WPB3	Transferase	**-8.1±0.0***
CMV protease	1LAY/P16753	Serine Protease	-7.1±0.0
Inositol 1 phosphatase	1LBV/O30298	Hydrolase	**-8.0±0.0***
Lysophospholipase	1LCL/Q05315	Serine Esterase	-6.6±0.0
Adipocyte-derived Leucine Aminopeptidase	1LCP/P00727	Hydrolase (alpha Aminoacylpeptide)	-7.5±0.0
Alcohol Dehydrogenase	1LDE/P00327	Dehydrogenase	**-8.0±0.0***
L-lactate Dehydrogenase	1LDM/P00341	Oxidoreductase	**-8.7±0.0***
Alcohol Dehydrogenase	1LDY/P00327	Dehydrogenase	-7.8±0.0
Glutamine Synthetase	1LGR/P0A1P6	Ligase(amide Synthetase)	-7.6±0.0
Lysozyme	1LMO/P11941	Hydrolase (o Glycosyl)	-7.2±0.0
Thermolysin	1LNA/P00800	Metalloprotease	-7.6±0.0
Casein Kinase II, Protein Kinase Ck2	1LP4/P28523	Transferase	**-9.8±0.0***
Triacylglycerol Lipase, Pancreatic	1LPM/P20261	Hydrolase	-6.6±0.0
Homocysteine S methyltransferase	1LT8/Q93088	Transferase	-6.5±0.0
Guanylate kinase	1LVG/Q64520	Transferase	**-8.8±0.0***
Cathepsin D	1LYW/P07339	Aspartic Protease	-7.6±0.0
Memapsin 2 (Beta-Secretase)	1M4H/P56817	Hydrolase/hydrolase Inhibitor	**-8.4±0.0***
Caspase-1	1M72/P89116	Hydrolase/hydrolase Inhibitor	-7.0±0.0
Nitric Oxide Synthase	1M9M/P29474	Oxidoreductase	**-8.4±0.0***
Tgf-beta Type II Receptor	1M9Z/P37173	Hormone/growth Factor	-6.9±0.0
Mandelate Racemase	1MDR/P11444	Racemase	-7.9±0.0
Inosine-5'-Monophosphate Dehydrogenase	1ME8/P50097	Oxidoreductase	**-8.3±0.0***
Matrilysin	1MMQ/P09237	Metalloprotease	-7.0±0.0
ATPase	1MO7/P06685	Hydrolase	-6.9±0.0

Glucosamine-6-Phosphate Synthase	1MOQ/P17169	Glutamine Amidotransferase	-7.9±0.0
Cytidine deaminase	1MQ0/P32320	Hydrolase	-7.5±0.0
Alpha-Momorcharin	1MRG/P16094	Ribosome Inactivating Protein	-7.4±0.0
Alpha-Trichosanthin	1MRK/P09989	Ribosome Inactivating Protein	-7.9±0.0
Aurora-2 protein kinase	1MUO/O14965	Transferase	-7.4±0.0
Phenylethanolamine N-Methyltransferase	1N7I/P11086	Transferase	**-9.0±0.0***
Lysyl oxidase	1N9E/Q96X16	Oxidoreductase	-7.9±0.0
Carnitine Acetyltransferase	1NDF/P47934	Transferase	**-8.0±0.0***
Succinate dehydrogenase	1NEK/P0AC41	Oxidoreductase/electron Transport	-7.4±0.0
Carnitine acyltransferase	1NM8/P43155	Transferase	**-8.0±0.0***
Chymase	1NN6/P23946	Hydrolase	-7.5±0.0
Neuramidase	1NNB/P03472	Hydrolase(o Glycosyl)	-7.2±0.0
Neuraminidase N9	1NNC/P03472	Hydrolase (o Glucosyl)	-7.3±0.0
3-dehydroquinate Synthase	1NR5/P07547	Lyase	**-8.6±0.0***
Neuramidase	1NSC/P27907	Hydrolase(o Glycosyl)	-7.1±0.0
MAP kinase-activated protein kinase 2	1NY3/P49137	Transferase	-7.9±0.0
Tyrosine-protein kinase transforming protein SRC	1NZL/P00524	Transferase	-5.9±0.0
Aspartate Aminotransferase, Cytoplasmic,Aspartate Transaminase	1O4S/Q9X0Y2	Transferase	-7.2±0.0
Activated Akt/Protein Kinase B	1O6L/P31751	Transferase	**-8.3±0.0***
Alpha mannosidase	1O7D/Q29451	Hydrolase	**-8.1±0.0***
Fibroblast Growth Factor Receptor 2	1OEC/P21802	Transferase	**-8.0±0.0***
Aldehyde Dehydrogenase, Mitochondrial	1OF7/P05091	Oxidoreductase	-7.0±0.0
Cytochrome P450 2C9	1OG5/P11712	Electron Transport	**-8.5±0.0***
Glucosylceramidase	1OGS/P04062	Hydrolase	-7.2±0.0
4-AMINOBUTYRATE AMINOTRANSFERASE	1OHY/P80147	Transferase	-6.9±0.0
Cell Division Protein Kinase 2	1OI9/P24941	Kinase	**-8.7±0.0***
Cell Division Protein Kinase 2	1OIU/P24941	Kinase	**-8.8±0.0***
Hyaluronate Lyase	1OJN/Q54873	Lyase	-7.3±0.0

Carbonic Anhydrase II	1OKL/P00918	Lyase	**-8.3±0.0***
3-methyl-2-oxobutanoate Dehydrogenase (Lipoamide)	1OLS/P12694	Oxidoreductase	-7.4±0.0
Oxygen-insensitive NAD(P)H nitroreductase	1OOQ/P38489	Oxidoreductase	-7.9±0.0
Peptidylglycine Alpha-Hydroxylating Monooxyg	1OPM/P14925	Oxidoreductase	-7.0±0.0
Ornithine Decarboxylase	1ORD/P43099	Carboxy Lyase	-7.8±0.0
Thermolysin	1OS0/P00800	Hydrolase/hydrolase Inhibitor	-7.3±0.0
Hepatitis C virus NS5B polymerase	1OS5/P26663	Transferase	-7.3±0.0
Dihydroorotate Dehydrogenase, Mitochondrial	1OVD/A2RJT9	Oxidoreductase	**-8.1±0.0***
Indole-3-Pyruvate Decarboxylase	1OVM/P23234	Lyase	-7.8±0.0
Aspartate Aminotransferase, Cytoplasmic	1OXO/P00508	Aminotransferase	-7.8±0.0
NADPH Dehydrogenase	1OYA/Q02899	Oxidoreductase (flavoprotein)	**-9.1±0.0***
Acetolactate Synthase, Catabolic	1OZF/P27696	Lyase	-7.8±0.0
Isopentenyl-diphosphate delta-isomerase	1P0N/P50740	Isomerase	-7.1±0.0
Glycogen Phosphorylase, Muscle Form	1P2D/P00489	Transferase	**-8.5±0.0***
p38 MAP kinase	1P38/P47811	Transferase	-7.7±0.0
glutamyl-endopeptidase	1P3C/Q9EXR9	Hydrolase	-7.3±0.0
Heme oxygenase 1	1P3U/Q9RGD9	Oxidoreductase	-7.3±0.0
Cysteine desulfurase	1P3W/P0A6B7	Lyase	-7.0±0.0
N-acetylglucosamine deacetylase	1P42/O67648	Hydrolase	-7.8±0.0
Glycogen phosphorylase, muscle form	1P4G/P00489	Transferase	-7.9±0.0
Riboflavin Kinase	1P4M/Q969G6	Transferase	**-9.4±0.0***
Thrombin	1P8V/P07359	Membrane Protein/hydrolase	-7.5±0.0
P-Hydroxybenzoate Hydroxylase	1PBD/P00438	Oxidoreductase	**-8.9±0.0***
Purine Nucleoside Phosphorylase	1PBN/P55859	Pentosyltransferase	-6.8±0.0
Coagulation Factor IX	1PFX/P16293	Hydrolase/hydrolase Inhibitor	**-8.0±0.0***
Aspartate Carbamoyltransferase	1PG5/Q55338	Transferase	-7.7±0.0
6-phosphogluconate Dehydrogenase	1PGP/P00349	Oxidoreductase (choh(d) Nadp+(a))	-6.7±0.0
Cytochrome P450-Cam	1PHD/ P00183	Oxidoreductase(oxygenase)	**-8.0±0.0***

Phosphatidylinositol 3-kinase	1PIC/P27986	Complex (phosphotransferase/receptor)	**-8.1±0.0***
alpha-Amylase	1PIG/P00690	Glycosyltransferase	-7.9±0.0
Cis-trans isomerase	1PIN/Q13526	Isomerase	-7.4±0.0
N, N-Dimethylglycine Oxidase	1PJ6/Q9AGP8	Oxidoreductase	**-9.4±0.0***
Casein Kinase II, Alpha Chain	1PJK/P68400	Transferase	**-8.8±0.0***
Sorbitol dehydrogenase	1PL7/Q00796	Oxidoreductase	-7.8±0.0
Glutathione transferase	1PMT/P15214	Transferase	-6.7±0.0
Phospholipase A2	1POC/P00630	Hydrolase	**-8.3±0.0***
Trypsin	1PPC/P00760	Hydrolase/hydrolase Inhibitor	-6.8±0.0
Trypsin	1PPH/P00760	Hydrolase/hydrolase Inhibitor	-6.9±0.0
alpha-Amylase	1PPI/P00690	Hydrolase (o Glycosyl)	**-8.1±0.0***
Penicillopepsin	1PPK/P00798	Hydrolase/hydrolase Inhibitor	-7.7±0.0
Cytochrome P450 2C8	1PQ2/P10632	Oxidoreductase	**-8.1±0.0***
Arginase II, mitochondrial precursor	1PQ3/P78540	Hydrolase	-7.5±0.0
2,4-Dienoyl-CoA Reductase	1PS9/P42593	Oxidoreductase	**-8.4±0.0***
Pepsin 3A	1PSO/P0DJD7	Hydrolase/hydrolase Inhibitor	-7.3±0.0
Phosphatidylinositol-Specific Phosphodiesterase C	1PTG/P14262	Hydrolase (phosphoric Diester)	-7.6±0.0
Protein kinase C delta	1PTR/P28867	Phosphotransferase	-6.3±0.0
Protein Tyrosine Phosphatase 1b	1PTY/P18031	Hydrolase	-7.6±0.0
DNA Topoisomerase II	1PVG/P06786	Isomerase	**-9.4±0.0***
Ornithine carbamoyltransferase	1PVV/Q51742	Transferase	-7.8±0.0
3,4-dihydroxy-2-butanone-4-phosphate Synthase	1PVY/Q60364	Isomerase	-7.5±0.0
Cyclin-dependent kinase-2	1PW2/P24941	Transferase	-7.0±0.0
Prostaglandin G/H Synthase 2	1PXX/Q05769	Oxidoreductase	**-9.2±0.0***
TGF-beta receptor type I	1PY5/P36897	Transferase	**-8.7±0.0***
Penicillin Binding Protein 2	1PYY/P59676	Transpeptidase	**-8.1±0.0***
2-amino-4-hydroxy-6-hydroxymethyldihydropteridine pyrophosphokinase	1Q0N/P26281	Transferase	**-8.0±0.0***
Histone deacetylase-2	1Q1A/P53686	Gene Regulation	-7.2±0.0
Intestinal Glcnac-6-sulfotransferase	1Q1Z/O00204	Transferase	**-8.7±0.0***

Steroid Sulphotransferase	1Q44/P52839	Transferase	-7.1±0.0
PLK-1 protein kinase	1Q4O/P53350	Transferase	-6.9±0.0
Prostaglandin-E2 9-reductase	1Q5M/P80508	Oxidoreductase	**-9.9±0.0***
3-hydroxy-3-methylglutaryl-coenzyme A Reductase	1QAX/P13702	Oxidoreductase	**-8.1±0.0***
Pokeweed Antiviral Protein	1QCI/P10297	Antiviral Protein	-7.8±0.0
Phospholipase A1	1QD6/P0A921	Membrane Protein	**-9.5±0.0***
Pentosyltransferase	1QE5/P81989	Transferase	-6.9±0.0
ATP-Dependent Helicase Pcra	1QHG/P56255	Hydrolase	-7.8±0.0
Thymidine Kinase	1QHI/P03176	Transferase	-6.3±0.0
Stromelysin	1QIA/P08254	Hydrolase	**-8.1±0.0***
Acetylcholinesterase	1QIJ/P04058	Hydrolase	**-8.6±0.0***
Lactoylglutathione lyase	1QIP/Q04760	Lyase	-7.6±0.0
Isopenicillin N Synthetase	1QIQ/P05326	Antibiotic Biosynthesis	**-8.4±0.0***
Ketosteroid Isomerase	1QJG/P00947	Isomerase	-7.0±0.0
Esterase	1QLW/Q7SIA5	Hydrolase(carboxylic Esterase)	-7.1±0.0
Methionine adenosyltransferase	1QM4/P13444	Transferase	-7.1±0.0
Thioredoxin peroxidase	1QMV/P32119	Oxidoreductase	-7.3±0.0
Elastase	1QNJ/P00772	Hydrolase (serine Protease)	-7.3±0.0
Pyruvate Decarboxylase	1QPB/P06169	Lyase	**-9.8±0.0***
LCK kinase	1QPC/P06239	Transferase	-7.4±0.0
LCK Tyrosine Kinase	1QPJ/P06239	Transferase	**-8.1±0.0***
Quinone reductase-2	1QR2/P16083	Oxidoreductase	-6.6±0.0
Quinone reductase	1QRD/P05982	Quinone Reductase (cytosolic)	-7.3±0.0
HIV-1 Integrase	1QS4/P12497	Transferase	-6.5±0.0
Ornithine Decarboxylase	1QU4/P07805	Lyase	**-8.1±0.0***
Hepatocyte Growth Factor Receptor	1R0P/P08581	Transferase	**-8.0±0.0***
ADP-ribosyl cyclase	1R12/P29241	Hydrolase	-7.9±0.0
Methyltransferase	1R18/Q27869	Transferase	**-8.2±0.0***
3-hydroxy-3-methylglutaryl-coenzyme A reductase	1R31/P13702	Oxidoreductase	-7.9±0.0
Lipase	1R50/P37957	Hydrolase	-5.9±0.0

Chorismate synthase	1R53/P28777	Lyase	-6.7±0.0
ADAM-33	1R55/Q9BZ11	Hydrolase	-7.1±0.0
Glycosyltransferase	1R82/P16442	Transferase	-6.8±0.0
Cytochrome P450 2C9	1R9O/P11712	Oxidoreductase	**-8.4±0.0***
Dihydrofolate Reductase	1RA2/P0ABQ4	Oxidoreductase	-7.4±0.0
CT610	1RCW/O84616	Oxidoreductase	-6.2±0.0
Methylmalonyl-coa Mutase	1REQ/P11653	Isomerase	**-8.8±0.0***
Coagulation Factor IX	1RFN/P00740	Coagulation Factor	-7.8±0.0
Ribonuclease	1RGE/P05798	Hydrolase (guanyloribonuclease)	-6.5±0.0
Renin	1RNE/ P00797	Hydrolase(acid Proteinase)	**-9.2±0.0***
Ribonuclease A	1ROB/P61823	Hydrolase(endoribonuclease)	-7.0±0.0
Deoxyhypusine synthase	1ROZ/P49366	Transferase	-7.1±0.0
HIV-1 Reverse Transcriptase	1RT6/P04585	Nucleotidyltransferase	**-8.8±0.0***
DNA Polymerase/reverse Transcriptase,HIV-1 Reverse Transcriptase	1RTD/P03366	Transferase/dna	-7.0±0.0
Murine Double Minute-2 (MDM2)	1RV1/Q00987	Ligase	-6.1±0.0
Mitogen-Activated Protein Kinase Kinase 2 (MEK2)	1S9I/P36507	Transferase	**-8.3±0.0***
Protoporphyrinogen oxidase	1SEZ/O24164	Oxidoreductase	**-9.7±0.0***
4-aminobutyrate aminotransferase	1SFF/P22256	Transferase	**-8.5±0.0***
Alanine Racemase	1SFT/P10724	Isomerase	-7.0±0.0
NRH dehydrogenase [quinone] 2	1SG0/P16083	Oxidoreductase	**-8.8±0.0***
Tyrosine-protein kinase ITK/TSK	1SM2/Q08881	Transferase	-7.8±0.0
Amylase	1SMD/P04745	Hydrolase (o Glycosyl)	-7.5±0.0
Thermonuclease Precursor	1SNC/P00644	Hydrolase (phosphoric Diester)	**-8.3±0.0***
Adenylosuccinate synthetase	1SON/P0A7D4	Ligase	**-9.0±0.0***
Pantothenate kinase	1SQ5/P0A6I3	Transferase	**-8.0±0.0***
Nuclease	1STB/P00644	Hydrolase(phosphoric Diester)	-7.7±0.0
Camp-dependent Protein Kinase A Type I	1STC/P00517	Complex (transferase/inhibitor)	**-8.3±0.0***
Glucokinase	1SZ2/P0A6V9	Transferase	-7.3±0.0
Alpha-galactosidase	1SZN/P0A6V9	Transferase	-6.6±0.0

Atrial Natriuretic Peptide Receptor A,Natriuretic peptide receptor A	1T34/P18910	Signaling Protein	**-8.1±0.0***
Aldehyde Reductase	1T41/P15121	Oxidoreductase	**-9.0±0.0***
Kit tyrosine kinase	1T46/P10721	Transferase Activator	**-8.6±0.0***
TAQ DNA Polymerase	1TAQ/P19821	Nucleotidyltransferase	-7.2±0.0
Hypoxanthine phosphoribosyltransferase	1TC1/Q4DRC4	Transferase	-7.4±0.0
Serine/Threonine Phosphatase B2	1TCO/P48452	Complex (hydrolase/isomerase)	-7.8±0.0
HIV Protease	1TCX/P04587	Hydrolase (acid Protease)	-7.6±0.0
Thymidylate Synthetase	1TDB/P00469	Transferase (methyltransferase)	-6.8±0.0
Threonine Deaminase	1TDJ/P04968	Allostery	-7.6±0.0
Beta-galactosidase	1TG7/Q700S9	Hydrolase	**-8.9±0.0***
Thymidylate Synthase	1TIS/P00471	Transferase(methyltransferase)	**-8.0±0.0***
S-adenosylmethionine decarboxylase	1TLU/Q9WZC3	Lyase	-6.2±0.0
Thermolysin	1TMN/P00800	Hydrolase/hydrolase Inhibitor	-7.7±0.0
Trypsin	1TNG/P00760	Hydrolase/hydrolase Inhibitor	-7.0±0.0
Tyrosine hydroxylase	1TOH/P04177	Hydroxylase	**-8.8±0.0***
Triosephosphate Isomerase	1TPH/P00940	Triosephosphate Isomerase	-7.0±0.0
Tyrosine Phenol-Lyase	1TPL/P31013	Lyase(carbon Carbon)	-7.9±0.0
Beta-Trypsin	1TPP/P00760	Hydrolase/hydrolase Inhibitor	-7.1±0.0
Beta-Secretase	1TQF/P56817	Hydrolase	**-8.2±0.0***
Thymidylate Synthetase	1TSD/P0A884	Transferase (methyltransferase)	**-8.1±0.0***
Tubulin	1TUB/P02550	Microtubules	**-8.1±0.0***
Reverse Transcriptase	1TVR/P03366	Aspartyl Protease	**-8.4±0.0***
Dihydropteroate Synthetase	1TX0/Q81VW8	Transferase	-7.2±0.0
P50-Rhogap	1TX4/Q07960	Complex (gtpase Activatn/proto Oncogene)	**-8.2±0.0***
Trypanothione Reductase	1TYP/P39040	Oxidoreductase	**-8.1±0.0***
Chalcone synthase 2	1U0W/P30074	Transferase	**-8.5±0.0***
L-Lactate dehydrogenase	1U4O/Q27743	Oxidoreductase	-7.1±0.0
Uracil-DNA Glycosylase	1UDH/P10186	Hydrolase	-6.9±0.0
Metalloprotease 3	1UEA/P08254	Complex (metalloprotease/inhibitor)	**-8.4±0.0***
Enoyl ACP reductase	1UH5/Q9BJJ9	Oxidoreductase	**-8.7±0.0***

Threonine Synthase	1UIM/P83823	Lyase	-7.7±0.0
SARS Coronavirus Main Proteinase (SARS-CoV 3CLpro)	1UK4/P0C6X7	Hydrolase	-6.5±0.0
Uridylmonophosphate/Cytidylmonophosphate Kinase	1UKE/P20425	Nucleotide Monophosphate Kinase	**-8.1±0.0***
c-jun N-terminal kinase (JNK1)	1UKI/P45983	Transferase	-6.8±0.0
Uridylate Kinase	1UKZ/P15700	Transferase	-6.7±0.0
Purine Nucleoside Phosphorylase	1ULB/P00491	Pentosyltransferase	**-8.1±0.0***
Aldose Reductase	1US0/P15121	Oxidoreductase	**-8.3±0.0***
5'-Nucleotidase	1USH/P07024	Hydrolase	-7.9±0.0
PDK1 protein kinase	1UU7/O15530	Transferase	-7.2±0.0
Thrombin	1UVT/P00735	Serine Protease	**-8.3±0.0***
Glyceraldehyde-3-phosphate dehydrogenase	1UXV/O57693	Oxidoreductase	-7.8±0.0
DNA primase small subunit	1V33/O57934	Transferase	-7.0±0.0
Glucokinase Isoform 2	1V4S/P35557	Transferase	-7.4±0.0
Adenosylhomocysteinase	1V8B/P50250	Hydrolase	**-9.1±0.0***
Arylesterase	1VA4/P22862	Hydrolase	-6.4±0.0
Acylamino-acid-releasing enzyme	1VE6/Q9YBQ2	Hydrolase	**-8.1±0.0***
D-amino acid oxidase	1VE9/P00371	Oxidoreductase	**-9.5±0.0***
Beta-amylase	1VEM/P36924	Hydrolase	**-8.3±0.0***
NAD(P)H\:FMN Oxidoreductase	1VFR/P46072	Oxidoreductase	-7.7±0.0
Shikimate kinase	1VIA/Q0PBC3	Transferase	-6.6±0.0
Catechol O-Methyltransferase	1VID/P22734	Transferase (methyltransferase)	-7.1±0.0
Urokinase	1VJA/P00749	Hydrolase	-7.3±0.0
Alpha glucosidase	1VJT/Q9WZL1	Hydrolase	**-8.1±0.0***
Histone deacetylase-8	1VKG/Q9BY41	Hydrolase	**-8.2±0.0***
Aldehyde oxidoreductase	1VLB/Q46509	Oxidoreductase	-7.1±0.0
Acetylcholinesterase	1VOT/P04058	Hydrolase	**-8.0±0.0***
ABC transporter, ATP-binding protein	1VPL/Q9WZ14	Lyase	-7.9±0.0
Creatine Kinase, M chain	1VRP/P04414	Transferase	-6.8±0.0
Thymidine Kinase	1VTK/P03176	Transferase	-6.9±0.0
Acetylcholinesterase	1VXR/P04058	Hydrolase	**-8.9±0.0***

Acyl CoA oxidase-1	1W07/O65202	Oxidoreductase	**-8.5±0.0***
Lysozyme	1W08/P61626	Hydrolase	-6.6±0.0
Beta-secretase-1	1W50/P56817	Hydrolase	**-8.0±0.0***
Phenylethylamine Oxidase	1W6G/P46881	Oxidoreductase	-7.3±0.0
Lanosterol Synthase	1W6K/P48449	Isomerase	**-10.1±0.0***
Kynurenine oxoglutarate transaminase	1W7L/Q16773	Transferase	-7.7±0.0
Platelet-activating Factor Acetylhydrolase	1WAB/Q29460	Platelet Factor	-6.9±0.0
Protein Tyrosine Phosphatase 1b	1WAX/P18031	Hydrolase	-6.4±0.0
Protein arginine deiminase IV	1WDA/Q9UM07	Hydrolase	-6.2±0.0
A-G adenine DNA glycosylase	1WEI/P17802	Hydrolase	**-8.3±0.0***
Transglycosylase	1WKD/P28720	TRNA Modifying Enzyme	**-8.0±0.0***
Guanine deaminase	1WKQ/O34598	Hydrolase	-6.4±0.0
Polyporopepsin	1WKR/P17576	Hydrolase/hydrolase Inhibitor	-7.0±0.0
Isoleucyl tRNA synthetase	1WNY/P56690	Ligase	**-8.1±0.0***
Agmatinase	1WOH/Q9RZ04	Hydrolase	-7.8±0.0
Ras-Ras Gap	1WQ1/P01112	Complex (gtp Binding/gtpase Activation)	**-8.1±0.0***
Uricase	1WRR/Q00511	Oxidoreductase	-7.5±0.0
GTP cyclohydrolase-I	1WUR/Q5SH52	Hydrolase	-6.6±0.0
TrkA receptor	1WWA/P04629	Transferase	-5.9±0.0
TrkB receptor	1WWB/Q16620	Transferase	-7.6±0.0
TrkC receptor	1WWC/Q16288	Transferase	-6.6±0.0
Tyrosinase	1WXC/Q83WS2	Oxidoreductase/metal Transport	-7.1±0.0
Protein geranylgeranyl transferase	1WY0/O58799	Transferase	-6.8±0.0
Undecaprenyl diphosphate synthetase	1X07/P60472	Transferase	**-8.3±0.0***
Syk tyrosine kinase	1XBA/P43405	Transferase	-7.5±0.0
hypothetical protein PA0115	1XEB/Q9I717	Transferase	**-8.2±0.0***
Glutamate decarboxylase	1XEY/P69908	Lyase	-6.9±0.0
putative acetyltransferase/acyltransferase	1XHD/Q816R4	Transferase	-6.8±0.0
Alanine transaminase	1XI9/Q9P9M8	Transferase	-7.0±0.0
D-Xylose Isomerase	1XID/P24300	Isomerase(intramolecular Oxidoreductase)	**-8.7±0.0***

Coagulation Factor Xa	1XKA/P00742	Blood Coagulation Factor	-7.8±0.0
Epidermal Growth Factor Receptor(EGFR)	1XKK/P00533	Transferase	**-9.3±0.0***
Peroxisomal carnitine O-octanoyltransferase	1XMC/Q9DC50	Transferase	**-8.0±0.0***
Thrombin light chain	1XMN/P00734	Hydrolase/hydrolase Inhibitor	**-8.1±0.0***
cAMP-specific 3',5'-cyclic phosphodiesterase 4D	1XOQ/Q08499	Hydrolase	**-8.5±0.0***
cAMP-specific 3',5'-cyclic phosphodiesterase 4B	1XOS/Q07343	Hydrolase	**-8.4±0.0***
3-hydroxy-3-methylglutaryl CoA synthase	1XPM/Q79ZY6	Transferase	**-8.5±0.0***
PIM-1 protein kinase	1XQZ/P11309	Transferase	-7.3±0.0
Glycerol kinase	1XUP/O34153	Transferase	-7.9±0.0
Uridine phosphorylase	1Y1T/P0A1F6	Transcription	-6.9±0.0
11-Hydroxysteroid dehydrogenase	1Y5M/P50172	Oxidoreductase	**-8.3±0.0***
Peptidyl dipeptidase Dcp	1Y79/P24171	Hydrolase	**-8.2±0.0***
Aspartate Aminotransferase	1YAA/P23542	Aminotransferase	-7.1±0.0
Heat shock protein 90	1YET/P07900	Chaperone Protein	-7.6±0.0
Yeast Glycogen Phosphorylase	1YGP/P06738	Glycosyltransferase	-7.8±0.0
Proto-oncogene tyrosine-protein kinase Src	1YOL/P12931	Transferase	**-8.0±0.0***
Inorganic Pyrophosphatase	1YPP/P00817	Hydrolase	-7.0±0.0
Serine carboxypeptidase	1YSC/P00729	Hydrolase(carboxypeptidase)	-7.3±0.0
falcipain-2	1YVB/Q9N6S8	Hydrolase/hydrolase Inhibitor	-6.5±0.0
Tyrosine-protein kinase JAK3	1YVJ/P52333	Transferase	-7.9±0.0
CLK1 protein kinase	1Z57/P49759	Transferase	**-8.8±0.0***
Carbonic anhydrase-III	1Z93/P07451	Lyase	-7.3±0.0
Inosine monophosphate Dehydrogenase	1ZFJ/P0C0H6	Oxidoreductase	
Enoyl-[acyl-carrier-protein] Reductase	1ZID/P9WGR1	Oxidoreductase	-7.9±0.0
Mannan-binding lectin serine protease-2	1ZJK/O00187	Hydrolase	-6.6±0.0
Histone lysine methyltransferase	1ZKK/Q9NQR1	Transferase	**-8.1±0.0***
Voltage gated potassium channel	1ZSX/Q13303	Oxidoreductase	**-9.2±0.0***
Topoisomerase IV	1ZVU/P0AFI2	Isomerase	
Map Kinase P38	1ZYJ/Q16539	Transferase	

Ribonucleotide reductase	1ZZD/P21524	Oxidoreductase	-7.7±0.0
Aldehyde reductase II	1ZZE/Q9UUN9	Oxidoreductase	-7.4±0.0
Mineralocorticoid receptor	2A3I/P08235	Transferase	-7.4±0.0
AMP deaminase	2A3L/O80452	Hydrolase	**-8.0±0.0***
WASP interacting protein	2A3Z/P68135	Structural Protein	**-8.8±0.0***
WASP interacting protein	2A41/P68135	Structural Protein	-7.6±0.0
Endonuclease III	2ABK/P0AB83	Endonuclease	-6.4±0.0
MAP kinase-interacting serine/threonine kinase 2	2AC3/Q9HBH9	Transferase	-6.8±0.0
Acetylcholinesterase	2ACE/P04058	Serine Hydrolase	**-8.4±0.0***
Acetylcholinesterase	2ACK/P04058	Hydrolase	**-8.0±0.0***
Adenosine Deaminase	2ADA/P03958	Hydrolase	-7.8±0.0
Tropinone Reductase-II	2AE2/P50163	Oxidoreductase	**-9.0±0.0***
Arginase I	2AEB/P05089	Hydrolase/hydrolase Inhibitor	-7.6±0.0
Orotate phosphoribosyltransferase	2AEE/Q9A076	Transferase	-6.8±0.0
Adamalysin II	2AIG/P34179	Hydrolast/hydrolase Inhibitor	**-8.2±0.0***
Adenylate Kinase Isoenzyme-3	2AK3/P08760	Transferase (phosphotransferase)	-6.9±0.0
Alkaline phosphatase	2ANH/P00634	Alkaline Phosphatase	-6.6±0.0
Plasma kallikrein, light chain	2ANY/P03952	Blood Clotting	-7.2±0.0
Insulin Receptor	2AUH/P06213	Transferase/signaling Protein	-7.0±0.0
Ubiquitin carboxyl-terminal hydrolase 14	2AYO/P54578	Hydrolase	-7.4±0.0
Methionine aminopeptidase 1	2B3K/P53582	Hydrolase	**-8.1±0.0***
NAD-dependent deacetylase sirtuin-5	2B4Y/Q9NXA8	Hydrolase	-7.1±0.0
Jak2 tyrosine kinase	2B7A/O60674	Transferase	**-8.0±0.0***
Cholesterol esterase	2BCE/P30122	Hydrolase	**-8.9±0.0***
Serine/threonine-protein kinase/endoribonuclease IRE1	2BE1/P32361	Transcription	-7.9±0.0
Glycogen synthase	2BFW/Q9V2J8	Transferase	-6.3±0.0
Glucose-6-phosphate dehydrogenase	2BH9/P11413	Oxidoreductase	**-8.2±0.0***
Mannosyltransferase	2BO6/Q9RFR0	Transferase	-7.9±0.0
Checkpoint kinase 1	2BRO/O14757	Transferase	**-8.2±0.0***
Pyruvate dehydrogenase kinase-2	2BU5/Q15119	Transferase	**-8.1±0.0***

5-Aminolevulinate synthase	2BWN/P18079	Transferase	-7.1±0.0
GTP cyclohydrolase-II	2BZ1/P0A7I7	Hydrolase	-7.6±0.0
Beta-mannosidase	2C0H/Q8WPJ2	Hydrolase	-7.5±0.0
PAK-6 protein kinase	2C30/Q9NQU5	Transferase	-6.1±0.0
Casein kinase-I gamma-2	2C47/P78368	Transferase	-7.3±0.0
Glutamate carboxypeptidase II	2C6C/Q04609	Hydrolase	**-9.7±0.0***
GMP reductase 2	2C6Q/Q9P2T1	Oxidoreductase	**-8.6±0.0***
Superoxide dismutase	2C9V/P00441	Oxidoreductase	-6.2±0.0
Carbonic anhydrase form B	2CAB/P00915	Hydro Lyase	-7.2±0.0
Ornithine Aminotransferase	2CAN/P04181	Transferase	-7.5±0.0
Hyaluronidase	2CBI/Q0TR53	Hydrolase	-6.7±0.0
Other phosphate group transferase	2CG5/Q9NRN7	Transferase/hydrolase	**-8.0±0.0***
Chorismate Mutase	2CHT/P19080	Isomerase	-7.9±0.0
Sentrin specific protease-1	2CKG/Q9P0U3	Hydrolase	-6.7±0.0
Protein Tyrosine Phosphatase 1b	2CMB/P18031	Hydrolase	-7.6±0.0
malate dehydrogenase	2CMD/P61889	Oxidoreductase(nad(a) Choh(d))	-6.7±0.0
Arginine deiminase	2CMU/O24890	Hydrolase	-6.6±0.0
Cytochrome P450 Reductase	2CPP/P00183	Oxidoreductase(oxygenase)	**-8.1±0.0***
Carboxypeptidase A	2CTC/P00730	Hydrolase(c-Terminal Peptidase)	-6.8±0.0
ADP ribosylglycohydrolase	2CWC/Q5SMG9	Hydrolase	-6.7±0.0
Acyl CoA oxidase-2	2DDH/P07872	Oxidoreductase	**-8.3±0.0***
D-alanine:D-alanine ligase	2DLN/P07862	Ligase(peptidoglycan Synthesis)	-6.8±0.0
Endothiapepsin	2ER6/P11838	Hydrolase/hydrolase Inhibitor	-7.9±0.0
Endothiapepsin	2ER7/P11838	Hydrolase/hydrolase Inhibitor	-7.4±0.0
Ribonuclease HII	2ETJ/Q9X017	Hydrolase	-6.8±0.0
CLK3 protein kinase	2EU9/P49761	Transferase	-7.6±0.0
2-Dehydropantoate 2-reductase	2EW2/Q831Q5	Oxidoreductase	-7.2±0.0
Acetyl-CoA acetyltransferase, mitochondrial	2F2S/P24752	Transferase	-7.6±0.0
Serine/threonine-protein kinase PAK 7	2F57/Q9P286	Transferase	-6.5±0.0
Cytochrome P450 2D6	2F9Q/P10635	Oxidoreductase	**-8.3±0.0***

Ubiquitin thiolesterase-7	2FOJ/Q93009	Hydrolase	-5.6±0.0
Geranyltranstransferase	2FTZ/Q9WY08	Transferase	-7.5±0.0
Dipeptidyl peptidase 4	2G5T/P27487	Hydrolase	-7.6±0.0
Falcipain 2	2GHU/Q9N6S8	Hydrolase	-6.8±0.0
Hepatitis A Protease 3C	2HAL/P08617	Hydrolase/hydrolase Inhibitor	-7.2±0.0
Hematopoetic Cell Kinase HCK	2HCK/P08631	Transferase	-7.9±0.0
Dipeptidyl Peptiidase IV (DPP4)	2HHA/P27487	Hydrolase	-7.8±0.0
HIV-1 Integrase	2ITG/P12497	DNA Integration	-7.0±0.0
p450 3A4 (CYP,CYP450)	2J0D/P08684	Oxidoreductase	-7.6±0.0
Glutamine Synthetase	2LGS/P0A1P6	Ligase(amide Synthetase)	-7.6±0.0
Inosine-Uridine Nucleoside N-Ribohydrolase	2MAS/Q27546	Hydrolase	-6.5±0.0
UDP-N-Acetylmuramate dehydrogenase	2MBR/P08373	Oxidoreductase	**-9.3±0.0***
Peptidylprolyl isomerase	2NUL/P23869	Isomerase	-6.6±0.0
PARP	2PAW/P26446	Transferase	**-8.8±0.0***
P-Hydroxybenzoate Hydroxylase	2PHH/P00438	Oxidoreductase	**-8.6±0.0***
Human Plasminogen Kringle 4	2PK4/P00747	Hydrolase(serine Protease)	-5.9±0.0
Poliovirus (Type 1, Mahoney Strain)	2PLV/P03300	Virus	-7.3±0.0
Rhinovirus 14 (HRV14)	2R07/P03303	Virus	-7.0±0.0
Neuraminidase	2SIM/P29768	Hydrolase	**-8.1±0.0***
HIV-1 Protease	2UPJ/P03367	Hydrolase (acid Protease)	**-8.2±0.0***
Penicillopepsin	2WEA/P00798	Hydrolase	-7.0±0.0
Penicillopepsin	2WEC/P00798	Hydrolase	-7.6±0.0
Hexokinase B	2YHX/P04807	Transferase	-7.3±0.0
Triose Phosphate Isomerase	2YPI/P00942	Triose Phosphate Isomerase	-7.4±0.0
Carbonic anhydrase-IV	2ZNC/Q64444	Lyase	**-8.0±0.0***
Beta lactamase	3BLM/P00807	Hydrolase	-7.1±0.0
Alcohol Dehydrogenase	3BTO/P00327	Oxidoreductase	**-8.2±0.0***
Histone deacetylase 7a	3C0Z/Q8WUI4	Hydrolase	-7.7±0.0
Type III Chloramphenicol Acetyltransferase	3CLA/P00484	Transferase (acyltransferase)	-7.5±0.0
Carboxypeptidase A	3CPA/P00730	Hydrolase (c Terminal Peptidase)	-7.1±0.0

Dihydrofolate Reductase	3DFR/P00381	Oxido Reductase	-7.9±0.0
Endothiapepsin	3ER3/P11838	Hydrolase/hydrolase Inhibitor	-7.6±0.0
Gamma Chymotrypsin	3GCH/P00766	Hydrolase (serine Proteinase)	-6.9±0.0
Heat shock protein 70	3HSC/P19120	Hydrolase (acting On Acid Anhydrides)	**-9.0±0.0***
Reverse Transcriptase	3HVT/P03366	Nucleotidyltransferase	**-8.5±0.0***
Medium chain acyl CoA dehydrogenase	3MDE/P41367	Oxidoreductase	**-9.2±0.0***
Phosphoglycerate kinase	3PGK/P00560	Transferase	**-8.1±0.0***
Trypsin	3PTB/P00760	Hydrolase (serine Proteinase)	-6.8±0.0
Thermolysin	3TMN/P00800	Hydrolase/hydrolase Inhibitor	-7.7±0.0
Trypsin	3TPI/P00760	Hydrolase/hydrolase Inhibitor	-6.9±0.0
Carbonic Anhydrase IV	3ZNC/Q64444	Lyase	-7.5±0.0
Methanol Dehydrogenase	4AAH/P38539	Oxidoreductase (pqq(a) Choh(d))	-7.6±0.0
Human Fibroblast Collagenase	4AYK/P03956	Matrix Metalloproteinase	**-8.4±0.0***
Phospholipase A2	4BP2/P00593	Carboxylic Ester Hydrolase Zymogen	**-8.2±0.0***
Carbonic anhydrase-II	4CAC/P00918	Lyase(oxo Acid)	-6.8±0.0
Chymotrypsin	4CHA/P00766	Hydrolase (serine Proteinase)	-7.1±0.0
Citrate Synthase	4CTS/P00889	Oxo Acid Lyase	**-8.1±0.0***
Dihydrofolate Reductase	4DFR/P0ABQ4	Oxido Reductase	**-8.6±0.0***
Endothiapepsin	4ER1/P11838	Hydrolase/hydrolase Inhibitor	-7.1±0.0
Pancreatic Elastase	4EST/P00772	Hydrolase/hydrolase Inhibitor	-7.1±0.0
influenza virus hemagglutinin	4HMG/P03437	Viral Protein	-6.5±0.0
Phenylalanine Hydroxylase	4PAH/P00439	Monooxygenase	**-8.1±0.0***
HIV-1 Protease	4PHV/P12497	Hydrolase(aspartic Proteinase)	**-8.3±0.0***
Phenylalanine Hydroxylase	4THI/P45741	Transferase	-7.9±0.0
Serine Proteinase alpha-thrombin	4THN/P00734	Hydrolase/hydrolase Inhibitor	-6.5±0.0
Thermolysin	4TMN/P00800	Hydrolase/hydrolase Inhibitor	-7.7±0.0
tyrosyl-tRNA synthetase	4TS1/P00952	Ligase (synthetase)	-7.9±0.0
Protein (Ribonuclease T1)	5BU4/P00651	Hydrolase	-6.8±0.0
Cytochrome P450Cam	5CP4/P00183	Oxidoreductase	**-8.3±0.0***
Carboxypeptidase A	5CPA/P00730	Hydrolase (c-Terminal Peptidase)	-7.4±0.0
Cytochrome P450 Reductase	5CPP/P00183	Oxidoreductase(oxygenase)	-7.8±0.0

Endothiapepsin	5ER2/P11838	Hydrolase/hydrolase Inhibitor	-7.2±0.0
Phospholipase A2	5P2P/P00592	Hydrolase(carboxylic Ester)	**-8.8±0.0***
Beta Trypsin	5PTP/P00760	Serine Protease	-7.1±0.0
Thermolysin	5TLN/P00800	Hydrolase (metalloproteinase)	-7.8±0.0
Thermolysin	5TMN/P00800	Hydrolase/hydrolase Inhibitor	-7.8±0.0
Cyclooxygenase-2	6COX/Q05769	Oxidoreductase	**-9.2±0.0***
Carboxypeptidase A	6CPA/P00730	Hydrolase (c-Terminal Peptidase)	-7.5±0.0
Cytochrome P450 Reductase	6CPP/P00183	Oxidoreductase(oxygenase)	**-8.0±0.0***
ribonuclease T1	6RNT/P00651	Hydrolase(endoribonuclease)	-6.0±0.0
Ribonuclease A	6RSA/P61823	Hydrolase	-6.5±0.0
Thermolysin	6TMN/P00800	Hydrolase/hydrolase Inhibitor	-7.5±0.0
Aspartate Aminotransferase	7AAT/P00508	Transferase(aminotransferase)	-7.3±0.0
Carboxypeptidase A	7CPA/P00730	Hydrolase(c-Terminal Peptidase)	-7.0±0.0
Cytochrome P450 Reductase	7CPP/P00183	Oxidoreductase(oxygenase)	**-8.1±0.0***
Triosephosphate Isomerase (bacterial)	7TIM/P00942	Intramolecular Oxidoreductase	-7.2±0.0
HIV-1 Protease	7UPJ/P03367	Hydrolase	-7.3±0.0
Protein (Hydroxynitrile Lyase)	7YAS/P52704	Lyase	-6.1±0.0
Catalase	8CAT/P00432	Oxidoreductase	**-8.2±0.0***
Cytochrome P450 Reductase	8CPP/P00183	Oxidoreductase(oxygenase)	**-8.2±0.0***
Gamma-chymotrypsin	8GCH/P00766	Hydrolase/peptide	-6.9±0.0
Triose Phosphate isomerase	8TIM/P00940	Isomerase	-7.0±0.0
Aspartate Aminotransferase	9AAT/P00508	Transferase (aminotransferase)	-7.8±0.0
HIV-1 Protease	9HVP/P12497	Hydrolase/hydrolase Inhibitor	-7.9±0.0
Isocitrate dehydrogenase	9ICD/P08200	Oxidoreductase (nad(a) Choh(d))	-7.3±0.0

Table S31. Docking affinity scores for trans-dityrosine binding to circadian clock related proteins.

Target name	PDB/UniProtKB	Type	Affinity (kcal/mol)
CK II beta	1QF8/P67870	Transferase	-6.5±0.0
CKI-gamma 1	2CMW/Q9HCP0	Transferase	-7.7±0.0

CK II alpha'	2E3B/P19784	Oxidoreductase	**-8.7±0.0***
CKI-gamma 3	2IZR/Q9Y6M4	Transferase	-7.5±0.0
EAR-1R	2V0V/Q14995	Transcription	-6.6±0.0
CLK1	2VAG/P49759	Transferase	**-8.5±0.0***
CLK4	2W96/P24385	Transferase	**-8.1±0.0***
CLK3	2WU6/P49761	Transferase	-7.8±0.0
CK II alpha	3H30/P68400	Transferase	**-8.0±0.0***
CLK2	3NR9/P49760	Transferase	**-8.2±0.0***

Table S32. Docking affinity scores for trans-dityrosine binding to insulin receptor pathway proteins.

Target Name	PDB/UniProtKB	Type	Affinity (kcal/mol)
Leptin	1AX8/P41159	Cytokine	-6.1±0.0
Protein sinI	1B0N/P23308	Transcription regulator	-6.6±0.0
PTEN	1D5R/P60484	Hydrolase	-7.4±0.0
SOS 1	1DBH/Q07889	Gene regulation	-7.1±0.0
PI3K-gamma	1E8Y/P48736	Serine/threonine protein kinase	**-8.4±0.0***
TNF-R1	1EXT/P19438	Signaling protein	-6.3±0.0
CBL	1FBV/P22681	Ligase	-7.3±0.0
GAPDH	1GPD/P00357	Oxidoreductase	-7.4±0.0
NEDD4	1I5H/Q62940	Ligase	-6.7±0.0
Insulin Receptor	1IR3/P06213	Transferase	-7.6±0.0
CRK	1JU5/P46108	Protein binding/transferase	-6.7±0.0
GRB2	1JYR/P62993	Signaling protein	-5.9±0.0
Insulin	1MSO/P01308	Hormone/growth factor	-5.7±0.0
Glucagon	1NAU/P01275	Hormone/growth factor	-5.9±0.0
GRB10	1NRV/Q13322	Signaling protein/cytokine	-6.3±0.0
SHC1	1OY2/P29353	Signaling protein	-6.6±0.0
Interleukin 6	1P9M/P05231	Signaling protein/cytokine	-7.0±0.0

IRS-1	1QQG/P35568	Signaling protein	-7.0±0.0
c-RAF	1RFA/P04049	Serine/threonine protein kinase	-5.8±0.0
PP-1B	1S70/P62140	Hydrolase	-7.4±0.0
EIF2B4	1T5O/O29877	Translation	-6.8±0.0
NCK2	1U5S/O43639	Metal binding protein	-6.6±0.0
GSK3B	1UV5/P49841	Transferase	**-8.0±0.0***
Synip	1WI4/Q9WV89	Protein binding	-5.9±0.0
Flot2	1WIN/Q60634	Cell adhesion	-6.2±0.0
eIF-4E	1WKW/P06730	Translation/protein binding	-7.2±0.0
SOS-1	1XD4/Q07889	Signaling protein	-7.5±0.0
PKC-theta	1XJD/Q04759	Transferase	-7.8±0.0
RHEB	1XTS/Q15382	Signaling protein	**-8.5±0.0***
ASP	1Y7J/P42127	Signaling protein	-6.2±0.0
Zinc finger protein 174	1Y7Q/Q15697	Transcription	-7.5±0.0
p59-Fyn	1ZBJ/P06241	Transferase	-7.9±0.0
PPAR-gamma	1ZGY/P37231	Transcription	-7.7±0.0
TC10	2ATX/P17081	Hydrolase	-7.0±0.0
EHBP1	2D89/Q8NDI1	Structural protein , protein binding	-6.3±0.0
hSTP	2EFK/Q15642	Endocytosis/exocytosis	-6.0±0.0
ERK2	2ERK/P63086	Phosphotransferase	-7.9±0.0
EHD1	2JQ6/Q9H4M9	Metal binding protein	-6.2±0.0
FOXO3	2K86/O43524	DNA binding protein	-5.9±0.0
mTOR	2NPU/P42345	Transferase	-7.9±0.0
MAPK 10	2P33/P53779	Transferase	**-8.3±0.0***
MEK1	2P55/Q02750	Transferase	-7.7±0.0
PKA C-alpha	2QCS/P05132	Transferase/transferase inhibitor	**-9.7±0.0***
AMPK subunit beta-2	2V8Q/O43741	Transferase	**-8.0±0.0***
PDE10A	2WEY/Q9Y233	Hydrolase	-7.8±0.0
Phospholipase C	2WXT/Q0TV31	Hydrolase	-7.2±0.0

AKT-3	2X18/Q9Y243	Transferase	-6.8±0.0
AKT-2	2X39/P31751	Transferase	**-8.4±0.0***
P70S6K1	3A62/P23443	Transferase	**-8.0±0.0***
FOXO1	3CO6/Q12778	Transcription/DNA	**-8.5±0.0***
Hemoglobin	3DUT/P69905/P68871	Oxygen binding	-7.4±0.0
PTP1B	3EAX/P18031	Hydrolase	-7.2±0.0
EIF2BA	3ECS/Q14232	Translation	-7.7±0.0
IRS2	3FQX/Q9Y4H2	Immune system	-7.7±0.0
SGK1	3HDN/O00141	Transferase	**-8.7±0.0***
iNOS	3HR4/P35228	Oxidoreductase/metal Binding Protein	**-8.6±0.0***
hPDK1	3IOP/O15530	Transferase	-7.6±0.0
PKC-alpha	3IW4/P17252	Transferase	-7.9±0.0
EIF2BE	3JUI/Q13144	Translation	-6.1±0.0
FOXO4	3L2C/P98177	Transcription/DNA	-7.7±0.0
TNF-a	3L9J/P01375	Immune system	-6.9±0.0
ACL	3MWD/P53396	Transferase	-7.8±0.0
SHP-2	3O5X/Q06124	Hydrolase	-6.6±0.0

Table S33. Docking affinity scores for trans-dityrosine binding to serum proteins.

Target name	PDB/UniProtKB	Type	Affinity (kcal/mol)
Topo1	1A35/P11387	Isomerase/DNA	**-8.9±0.0***
TCP228	1B3A/P13501	Anti HIV protein	-6.2±0.0
MCAF	1DOK/P13500	Chemoattractant	-6.2±0.0
ALB	1E7A/P02768	Carrier protein	-7.9±0.0
PTP-1C	1FPR/P29350	Signaling protein	-7.5±0.0
MIP-4-alpha	1G2S/Q9Y258	Cytokine	-7.1±0.0
CKB-8	1G91/P55773	Cytokine	-6.3±0.0
LAG-1	1HUM/P13236	Cytokine(chemotactic)	-5.8±0.0
AE 1	1HYN/P02730	Membrane protein	**-8.1±0.0***
ApoC-II	1I5J/P02655	Lipid transport	-5.7±0.0

BPK	1K2P/Q06187	Transferase	-6.7±0.0
TCPTP	1L8K/P17706	Hydrolase	-6.8±0.0
PAI-1	1LJ5/P05121	Membrane protein	-6.5±0.0
HLA-B8	1M05/P30460	Immune system	**-8.2±0.0***
MIP-3-alpha	1M8A/P78556	Cytokine	-6.3±0.0
APOE	1NFN/P02649	Lipid transport	-6.8±0.0
C1 esterase	1NZI/P09871	Hydrolase	-6.5±0.0
B2M	1PY4/P61769	Signaling protein	-6.3±0.0
SL-2	1Q3A/P09238	Hydrolase	**-9.1±0.0***
HLA-A	1QVO/P13746	Immune system	**-8.6±0.0***
ADAM33	1R54/Q9BZ11	Hydrolase	-6.5±0.0
G-CSF	1RHG/P09919	Growth factor	-6.6±0.0
R-PTP-mu	1RPM/P28827	Receptor	-7.3±0.0
APP	1RW6/P05067	Cell adhesion	-6.3±0.0
HLA-A1	1W72/P30443	Immune system	-7.4±0.0
R-PTP-delta	1X5Z/P23468	Hydrolase	-5.4±0.0
MME	1Y93/P39900	Hydrolase	-7.8±0.0
L-CA	1YGR/P08575	Hydrolase	-7.5±0.0
HEPTP	1ZC0/P35236	Hydrolase	-6.9±0.0
R-PTP-R	2A8B/Q15256	Hydrolase	-7.1±0.0
TNF-a	2AZ5/P01375	Blood clotting	-6.7±0.0
PTP-H1	2B49/P26045	Hydrolase	-6.9±0.0
STEP	2BIJ/P54829	Hydrolase	-6.0±0.0
PTPN14	2BZL/Q15678	Hydrolase	-7.5±0.0
R-PTP-kappa	2C7S/Q15262	Hydrolase	-6.6±0.0
SSAO	2C10/Q16853	Oxidoreductase	-6.9±0.0
PTP-1B	2CM2/P18031	Hydrolase	-6.4±0.0
ADAM 17	2DDF/P78536	Hydrolase	**-8.2±0.0***
R-PTP-S	2FH7/Q13332	Hydrolase	-7.2±0.0
PTP-U2	2GJT/Q16827	Hydrolase	-7.0±0.0
GM-CSF	2GMF/P04141	Growth factor	-6.6±0.0

R-PTP-gamma	2H4V/P23470	Hydrolase	-7.4±0.0
R-PTP-beta	2HC1/P23467	Hydrolase	-7.0±0.0
MIP-2G	2HDL/O95715	Chemokine	-5.7±0.0
C1Q	2JG9/P02745/P02746/P02747	Membrane adhesion	-6.6±0.0
ADAM-TS 1	2JIH/Q9UHI8	Hydrolase	**-8.6±0.0***
R-PTP-epsilon	2JJD/P23469	Hydrolase	-7.4±0.0
MMP-20	2JSD/O60882	Hydrolase	-7.6±0.0
PTPN18	2OC3/Q99952	Hydrolase	-7.1±0.0
R-PTP-T	2OOQ/O14522	Hydrolase	-6.6±0.0
MEG2	2PA5/P43378	Hydrolase	-6.7±0.0
F8/G253 antigen	2PF6/P50895	Cell adhesion	-6.2±0.0
IAR	2QEP/Q92932	Hydrolase	-7.3±0.0
R-PTP-N	2QT7/Q16849	Hydrolase	-5.1±0.0
C1R	2QY0/P00736	Hydrolase	-7.8±0.0
ADAM-TS 4	2RJP/O75173	Hydrolase	**-8.2±0.0***
CCR5	2RLL/P51681	Membrane protein	-5.4±0.0
MEG	2VPH/P29074	Hydrolase	-6.6±0.0
HLE	2Z7F/P08246	Hydrolase/hydrolase Inhibitor	-7.4±0.0
PTP-1D	3B7O/Q06124	Hydrolase	-7.0±0.0
ADAM-TS 5	3B8Z/Q9UNA0	Hydrolase	**-8.8±0.0***
TIMP-3	3CKI/P3562z5	Hydrolase, hydrolase inhibitor	-7.4±0.0
Alpha-1-antitrypsin	3DRM/P01009	Hydrolase inhibitor	-7.4±0.0
ADORA2A	3EML/P29274	Membrane protein , receptor	**-8.1±0.0***
Cnt-a1	3FEH/O75689	Hydrolase activator	-6.5±0.0
MIP-1-alpha	3FPU/P10147	Immune system	-6.3±0.0
ADAM 22	3G5C/Q9P0K1	Membrane protein	**-8.4±0.0***
ADAM-TS 13	3GHM/Q76LX8	Hydrolase	-7.6±0.0
Galactohydrolase	3HG3/P06280	Hydrolase	-6.8±0.0
ALT2	3IHJ/Q8TD30	Transferase	-6.8±0.0
TopBP1	3JVE/Q92547	Protein binding	-6.2±0.0
R-PTP-zeta	3JXF/P23471	Cell adhesion	-7.3±0.0

AGP 1	3KQ0/P02763	Signaling protein	-7.5±0.0
MMP-13	830C/P45452	Matrix metalloprotease	**-8.3±0.0***

Table S34. Docking affinity scores for trans-dityrosine binding to breast cancer proteins.

Target name	PDB/UniProtKB	Type	Affinity (kcal/mol)
TIMP-2	1BR9/P16035	Hydrolase	-7.4±0.0
MMP-2	1CK7/P08253	Hydrolase	-7.4±0.0
BAX	1F16/Q07812	Apoptosis	-6.6±0.0
Smac	1FEW/Q9NR28	Apoptosis	-6.0±0.0
IFNG	1FYH/P01579	Immune system	-7.4±0.0
MMP-9	1GKC/P14780	Hydrolase	**-8.8±0.0***
PS2 protein	1HI7/P04155	Growth factor	-5.6±0.0
Stromelysin-1	1HY7/P08254	Hydrolase	-7.8±0.0
IL1RN	1IRP/P18510	Cytokine	-6.7±0.0
LAR	1LAR/P10586	Hydrolase	-7.8±0.0
VHL	1LM8/P40337	Transcription	-6.7±0.0
RAD51	1N0W/Q06609	Gene regulation/antitumor protein	-7.0±0.0
Caspase-9	1NW9/P55211	Apoptosis	-7.1±0.0
MMP-16	1RM8/P51512	Hydrolase	**-9.1±0.0***
BRIP1	1T29/Q9BX63	Signaling protein	-6.3±0.0
ER-alpha	1XPC/P03372	Nuclear receptor	-7.8±0.0
DNA topoisomerase II	1ZXM/P11388	Isomerase	**-8.5±0.0***
AIF	2G2B/P55008	Immune system	-7.4±0.0
Protein FADD	2GF5/Q13158	Apoptosis	-7.5±0.0
DEP-1	2NZ6/Q12913	Hydrolase	-6.4±0.0
Bcl-2	2O21/P10415	Apoptosis	-7.2±0.0
RAIDD	2O71/P78560	Apoptosis	-5.7±0.0
LyP	2P6X/Q9Y2R2	Hydrolase	-7.5±0.0
CHEK2	2W0J/O96017	Transferase	**-8.2±0.0***
PALB2	2W18/Q86YC2	Nuclear protein	-7.4±0.0

MMP-14	3C7X/P50281	Hydrolase	-7.2±0.0
p53	3D06/P04637	Transcription	-6.2±0.0
Caspase-3	3H0E/P42574	Hydrolase	-7.2±0.0
Caspase-8	3H11/Q14790	Apoptosis	-7.3±0.0
Beta-G1	3HN3/P08236	Hydrolase	-7.4±0.0
Mcl-1	3KJ0/Q07820	Transferase	-6.5±0.0
RAR-alpha	3KMR/P10276	Transcription	-7.3±0.0
FAP-1	3LNX/Q12923	Signaling protein	-6.6±0.0
Beta-2 adrenoceptor	3NY8/P07550	Membrane protein	**-9.5±0.0***

Table S35. Results of the refinement docking experiments for cis-dityrosine with AutoDock Vina.

Target name	PDB/UniProtKB	Type	Affinity (kcal/mol)
Binding Proteins			
Intestinal Fatty Acid Binding Protein	1ICN/P02693	Binding Protein(fatty Acid)	**-9.1±0.0**
Streptavidin	1SRJ/P22629	Biotin Binding Protein	**-8.1±0.0**
Uteroglobin	1UTR/P17559	Mammalian Pcb Binding Protein	**-8.2±0.0**
Intestinal Fatty Acid Binding Protein	2IFB/P02693	Fatty Acid Binding Protein	**-8.9±0.0**
Nuclear Receptors			
Cellular Retinoic-Acid-Binding Protein Type II	1CBS/P29373	Retinoic Acid Transport	**-8.1±0.0**
Nuclear Vitamin D Receptor	1DB1/P11473	Gene Regulation	**-8.3±0.0**
Lac Repressor	1EFA/P03023	Transcription/dna	**-9.0±0.0**
Nuclear receptor ROR-alpha	1N83/P35398	Lipid Binding Protein	**-8.2±0.0**
Thyroid hormone receptor Alpha-1	1NAV/P10827	Membrane Protein	**-8.2±0.0**
Liver X receptor alpha	1UHL/P28702	DNA Binding Protein	**-8.0±0.0**
Retinoic acid receptor RXR-alpha	1XVP/Q14994	DNA Binding Protein	**-8.3±0.0**
Peroxisome proliferator activated receptor delta	2AWH/Q03181	Gene Regulation	**-8.7±0.0**
Transport Proteins			
Cellular Retinoic-Acid-Binding Protein	1CBS/P29373	Retinoic Acid Transport	**-8.0±0.0**
Nonspecific lipid transfer protein	1CZ2/P24296	Lipid Binding Protein	**-8.2±0.0**
Cytochrome C2	1I8O/P00091	Electron Transport	**-8.8±0.0**
Plasma retinol-binding protein	1IIU/P41263	Transport Protein	**-8.4±0.0**
human retinol-binding protein	1QAB/P02753	Transport Protein	**-8.3±0.0**
Serum protein	2BX8/P02768	Transport Protein	**-8.4±0.0**
Receptors			
Retinoid X Receptor-alpha	1DKF/P10276	Hormone/growth Factor Receptor	**-8.9±0.0**
Peroxisome Proliferator Activated Receptor gamma (PPAR gamma)	1FM6/P37231	Transcription	**-8.2±0.0**

Phosphatidylinositol 3-kinase	1PIC/P27986	Complex (phosphotransferase/receptor)	**-8.2±0.0**
Oxysterols receptor LXR-beta	1UPV/P55055	Receptor	**-8.4±0.0**
Constitutive androstane alpha receptor	1XNX/O35627	Ligand Receptor/transcription Regulation	**-8.0±0.0**
Maltose-binding periplasmic protein	1YTV/P0AEX9	Sugar Binding Protein	**-8.2±0.0**
Monoclonal Antibodies			
Fab' Fragment Of Monoclonal Antibody Db3	1DBM/P01868	Immunoglobulin	**-8.5±0.0**
Human Class I Histocompatibility Antigen	1HHG/P01892	Histocompatibility Antigen	**-8.3±0.0**
Immunoglobulin lambda Light Chain Dimer (Mcg)	1MCJ	Immunoglobulin	**-8.9±0.0**
IGG1-KAPPA DB3 FAB	2DBL/P01868	Immunoglobulin	**-8.3±0.0**
4-4-20 (IgG2A) Fab Fragment	4FAB/P01865	Immunoglobulin	**-8.3±0.0**
Factor, Regulator and Hormones			
Elongation Factor Tu	1HA3/Q5SHN6	Translation	**-8.0±0.0**
Heat Shock 70 kDa protein 8	1HX1/P19120	Chaperone/chaperone Inhibitor	**-8.8±0.0**
Nuclear Orphan Receptor Lxr-beta	1K4W/P45446	Hormone/growth Factor	**-8.4±0.0**
Gonadotropin alpha subunit	1QFW/P01215	Immune System	**-6.9±0.0**
Structural Proteins			
ATP binding cassette	1F3O/Q58206	Structural Genomics	**-8.1±0.0**
Tubulin alpha chain	1JFF/P02550	Structural Protein	**-8.1±0.0**
Signaling Proteins			
Ran-GPPNHP-RanBP1-RanGAP	1K5D/P62826	Signaling Protein	**-8.9±0.0**
Ion Channels			
Voltage-Gated Potassium Channel	1JVM/P0A334	Membrane Protein	**-8.6±0.0**
Lipid Binding Protein			
KES1 protein	1ZHY/P35844	Lipid Binding Protein	**-8.6±0.0**
Undefined receptors			
bilin binding protein (BBP)	1BBP/P09464	Bilin Binding	**-8.7±0.0**
Actin, alpha skeletal muscle	1NWK/P68135	Contractile Protein	**-9.0±0.0**
Enzymes			
Alpha-thrombin	1A4W/P00734	Hydrolase/hydrolase Inhibitor	**-8.6±0.0**
Plasminogen Activator Inhibitor Type 1	1A7C/P05121	Hydrolase Inhibitor/peptide	**-7.9±0.0**
Cytochrome Peroxidase	1AC8/P00431	Oxidoreductase	**-8.0±0.0**
Alcohol Dehydrogenase	1ADC/P00327	Oxidoreductase (nad(a) Choh(d))	**-8.0±0.0**
Serine Proteinase alpha-thrombin	1AE8/P00734	Hydrolase/hydrolase Inhibitor	**-8.2±0.0**
TGF-beta receptor type I	1B6C/P36897	Complex (isomerase/protein Kinase)	**-7.7±0.0**
Fructose biphosphatase	1BIF/P25114	Bifunctional Enzyme	**-9.6±0.0**
Serine Proteinase alpha-thrombin	1BMN/P00734	Hydrolase/hydrolase Inhibitor	**-8.0±0.0**
Dihydrofolate Reductase	1BOZ/P00374	Oxidoreductase	**-8.4±0.0**
Liver Alcohol Dehydrogenase	1BTO/P00327	Oxidoreductase	**-8.1±0.0**
Protein (Cytochrome P450 BM-3)	1BVY/P14779	Oxidoreductase	**-8.5±0.0**
Beta-Amylase	1BYB/P10538	Hydrolase(o Glycosyl)	**-8.3±0.0**

Dihydrofolate Reductase	1BZF/P00381	Oxidoreductase	**-8.1±0.0**
Neutrophil Collagenase	1BZS/P22894	Hydrolase	**-8.8±0.0**
Ras-Binding Domain of the Serine/Threonine Kinase C-Raf1	1C1Y/P62834	Signaling Protein	**-8.9±0.0**
Lumazine Synthase	1C41/Q9UVT8	Transferase	**-8.9±0.0**
Ornithine Decarboxylase	1C4K/P43099	Lyase	**-8.0±0.0**
Glutamate Mutase	1CB7/P80078	Isomerase	**-8.4±0.0**
Cobalt-Precorrin-4 Transmethylase	1CBF/O87696	Methyltransferase	**-8.4±0.0**
Pnp Oxidase	1CI0/P38075	Oxidoreductase	**-8.2±0.0**
Thymidylate Synthase	1CI7/P13100	Transferase	**-8.2±0.0**
Cholesterol Oxidase	1COY/P22637	Oxidoreductase(oxygen Receptor)	**-9.1±0.0**
Cytochrome P450-Terp	1CPT/P33006	Oxidoreductase(oxygenase)	**-8.9±0.0**
Epoxide hydrolase	1CQZ/P34914	Hydrolase	**-8.4±0.0**
Casein kinase I	1CSN/P40233	Phosphotransferase	**-8.2±0.0**
Cyclooxygenase-2	1CX2/Q05769	Oxidoreductase	**-8.7±0.0**
Myeloperoxidase	1D2V/P05164	Oxidoreductase	**-8.0±0.0**
Dihydroorotate Dehydrogenase, Mitochondrial	1D3G/Q02127	Oxidoreductase	**-9.4±0.0**
Hypoxanthine-Guanine Phosphoribosyltransferase	1D6N/P00492	Transferase	**-9.6±0.0**
Dihydrofolate Reductase	1DHF/P00374	Oxido Reductase	**-8.0±0.0**
Dihydrofolate Reductase	1DHJ/P0ABQ4	Oxidoreductase	**-8.5±0.0**
Estrogenic 17-Beta Hydroxysteroid Dehydrogenase	1DHT/P14061	Oxidoreductase	**-8.1±0.0**
Cyclin-Dependent Kinase 2 (Cdk2)	1DI8/P24941	Transferase	**-8.3±0.0**
D-Xylose Isomerase	1DID/P12070	Isomerase(intramolecular Oxidoreductase)	**-8.1±0.0**
Dihydrofolate Reductase	1DIS/P00381	Oxido Reductase	**-9.2±0.0**
Phenylalanine Hydroxylase	1DMW/P00439	Oxidoreductase	**-8.3±0.0**
Dihydrofolate Reductase	1DR1/P00378	Oxidoreductase	**-8.2±0.0**
Serine Proteinase alpha-thrombin	1DWC/P00734	Hydrolase/hydrolase Inhibitor	**-8.1±0.0**
beta-Glucosidase	1E1F/P49235	Hydrolase	**-9.6±0.0**
GDP fucose synthetase	1E6U/P32055	Epimerase/reductase	**-8.0±0.0**
GDP-Fucose Synthetase	1E7S/P32055	Epimerase/reductase	**-8.6±0.0**
Pteridine Reductase	1E7W/Q01782	Oxidoreductase	**-8.1±0.0**
Nitric Oxide Synthase	1ED5/P29473	Oxidoreductase	**-8.5±0.0**
HIV-1 Reverse Transcriptase	1EET/P03366	Viral Protein	**-8.5±0.0**
Bovine Mitochondrial F1-ATPase	1EFR/P19483/P00829/P05631	Hydrolase/antibiotic	**-9.6±0.0**
D-Amino acid oxidase	1EVI/P00371	Oxidoreductase	**-8.7±0.0**
Glycerol-3-phosphate dehydrogenase	1EVZ/P90551	Oxidoreductase	**-8.2±0.0**
Lipoxygenas-1	1F8N/P08170	Oxidoreductase	**-8.1±0.0**
Carboxylase	1FIY/P00864	Complex (lyase/inhibitor)	**-9.3±0.0**
Peptidase T	1FNO/P26311	Hydrolase	**-8.2±0.0**
Protein farnesyl transferase	1FT2/Q04631	Transferase	**-8.4±0.0**
Glucose phosphate thymidylyltransferase	1FXO/Q9HU22	Transferase	**-8.9±0.0**
Cell Division Protein Kinase 2(CDK2)	1GIH/P24941	Transferase	**-8.6±0.0**
Cyclin Dependent Kinase 2(Cdk2)	1GII/P24941	Transferase	**-8.0±0.0**
Monoamine Oxidase	1GOS/P27338	Oxidoreductase	**-9.4±0.0**
Glycogen Phosphorylase B	1GPB/P00489	Glycogen Phosphorylase	**-9.1±0.0**

Acetylcholinesterase	1GPK/P04058	Hydrolase	**-8.3±0.0**
GMP synthetase	1GPM/P04079	Transferase (glutamine Amidotransferase)	**-8.3±0.0**
Acetylcholinesterase	1GQS/P04058	Hydrolase	**-9.0±0.0**
Glutathione reductase	1GRE/P00390	Oxidoreductase	**-8.1±0.0**
Glutathione Transferase A1-1	1GSF/P08263	Transferase (glutathione)	**-8.3±0.0**
Nitrogenase Molybdenum Iron Protein Alpha Chain	1H1L/P00466	Oxidoreductase	**-8.4±0.0**
CDK2	1H28/P24941	Cell Cycle/transferase Substrate	**-8.0±0.0**
Dihydropyrimidine dehydrogenase	1H7X/Q28943	Electron Transfer	**-8.5±0.0**
Polyamine oxidase	1H82/O64411	Oxidoreductase	**-9.3±0.0**
Elongation Factor Tu	1HA3/Q5SHN6	Translation	**-8.8±0.0**
Isopenicillin N Synthase	1HB2/P05326	Antibiotic Biosynthesis	**-8.3±0.0**
Human Class I Histocompatibility Antigen	1HHJ/P01892	Histocompatibility Antigen	**-8.4±0.0**
Alcohol Dehydrogenase	1HLD/P00327	Oxidoreductase(ch Oh(d) Nad(a))	**-8.4±0.0**
Prophospholipase A2	1HN4/P00592	Hydrolase	**-9.4±0.0**
HIV-1 Protease	1HPS/P03366	Hydrolase(acid Proteinase)	**-8.3±0.0**
Leukotriene A4 hydrolase	1HS6/P09960	Hydrolase	**-8.9±0.0**
Prostaglandin G/H Synthase 1, COX-1	1HT8/P05979	Oxidoreductase	**-8.3±0.0**
Neutrophil Collagenase, MMP8	1I76/P22894	Hydrolase	**-7.3±0.0**
Methionine decarboxylase	1I7B/P17707	Lyase	**-8.8±0.0**
Thiamin Pyrophosphokinase	1IG3/Q9R0M5	Transferase	**-8.9±0.0**
Cholesterol Oxidase	1IJH/P12676	Oxidoreductase	**-9.0±0.0**
Aldo-Keto Reductase Family 1 Member C3	1J96/P52895	Oxidoreductase	**-9.0±0.0**
HIV Protease	1JKH/P04585	Transferase	**-8.3±0.0**
Methylthioadenosine phosphorylase	1JP7/P50389	Transferase	**-8.2±0.0**
Histamine N-methyltransferase	1JQE/P50135	Transferase	**-8.7±0.0**
Inosine-5'-monophosphate Dehydrogenase 2,IMP-2 dehydrogenase	1JR1/P12269	Oxidoreductase	**-8.4±0.0**
Aromatic-L-amino-acid Decarboxylase	1JS3/P80041	Lyase	**-8.7±0.0**
Dihydroorotate Dehydrogenase	1JUE/A2RJT9	Oxidoreductase	**-8.5±0.0**
NADPH Dehydrogenase	1K02/Q02899	Oxidoreductase	**-8.8±0.0**
Glutathione Transferase A1-1	1K3Y/P08263	Transferase	**-8.9±0.0**
Nicotinate-nucleotide Adenylyltransferase	1K4M/P0A752	Transferase	**-8.2±0.0**
Transforming Protein P21/H-Ras-1	1K8R/P01112	Signaling Protein	**-8.5±0.0**
Histidinol Dehydrogenase	1KAE/P06988	Oxidoreductase	**-8.1±0.0**
Mycolic Acid Synthase	1L1E/P9WPB3	Transferase	**-8.3±0.0**
Inositol 1 phosphatase	1LBV/O30298	Hydrolase	**-8.2±0.0**
Alcohol Dehydrogenase	1LDE/P00327	Dehydrogenase	**-8.2±0.0**
Casein Kinase II, Protein Kinase Ck2	1LP4/P28523	Transferase	**-9.5±0.0**
Memapsin 2 (Beta-Secretase)	1M4H/P56817	Hydrolase/hydrolase Inhibitor	**-8.4±0.0**
Nitric Oxide Synthase	1M9M/P29474	Oxidoreductase	**-8.6±0.0**
Carnitine acyltransferase	1NM8/P43155	Transferase	**-8.0±0.0**
3-dehydroquinate Synthase	1NR5/P07547	Lyase	**-8.4±0.0**
Alpha mannosidase	1O7D/Q29451	Hydrolase	**-8.1±0.0**
Fibroblast Growth Factor Receptor 2	1OEC/P21802	Transferase	**-7.9±0.0**

Cell Division Protein Kinase 2	1OIU/P24941	Kinase	**-8.2±0.0**
Oxygen-insensitive NAD(P)H nitroreductase	1OOQ/P38489	Oxidoreductase	**-8.1±0.0**
NADPH Dehydrogenase	1OYA/Q02899	Oxidoreductase (flavoprotein)	**-9.1±0.0**
Glycogen Phosphorylase, Muscle Form	1P2D/P00489	Transferase	**-8.9±0.0**
P-Hydroxybenzoate Hydroxylase	1PBD/P00438	Oxidoreductase	**-8.7±0.0**
Cytochrome P450-Cam	1PHD/ P00183	Oxidoreductase(oxygenase)	**-8.1±0.0**
Phosphatidylinositol 3-kinase	1PIC/P27986	Complex (phosphotransferase/receptor)	**-8.2±0.0**
N, N-Dimethylglycine Oxidase	1PJ6/Q9AGP8	Oxidoreductase	**-8.9±0.0**
Casein Kinase II, Alpha Chain	1PJK/P68400	Transferase	**-8.6±0.0**
2,4-Dienoyl-CoA Reductase	1PS9/P42593	Oxidoreductase	**-8.4±0.0**
DNA Topoisomerase II	1PVG/P06786	Isomerase	**-9.3±0.0**
Prostaglandin G/H Synthase 2	1PXX/Q05769	Oxidoreductase	**-9.2±0.0**
TGF-beta receptor type I	1PY5/P36897	Transferase	**-8.8±0.0**
2-amino-4-hydroxy-6-hydroxymethyldihydropteridine pyrophosphokinase	1Q0N/P26281	Transferase	**-9.4±0.0**
Prostaglandin-E2 9-reductase	1Q5M/P80508	Oxidoreductase	**-9.7±0.0**
3-hydroxy-3-methylglutaryl-coenzyme A Reductase	1QAX/P13702	Oxidoreductase	**-8.3±0.0**
Phospholipase A1	1QD6/P0A921	Membrane Protein	**-9.2±0.0**
Stromelysin	1QIA/P08254	Hydrolase	**-7.9±0.0**
Isopenicillin N Synthetase	1QIQ/P05326	Antibiotic Biosynthesis	**-8.3±0.0**
Pyruvate Decarboxylase	1QPB/P06169	Lyase	**-9.2±0.0**
ADP-ribosyl cyclase	1R12/P29241	Hydrolase	**-8.0±0.0**
Methyltransferase	1R18/Q27869	Transferase	**-8.1±0.0**
Methylmalonyl-coa Mutase	1REQ/P11653	Isomerase	**-8.3±0.0**
Renin	1RNE/ P00797	Hydrolase(acid Proteinase)	**-9.0±0.0**
HIV-1 Reverse Transcriptase	1RT6/P04585	Nucleotidyltransferase	**-8.9±0.0**
Protoporphyrinogen oxidase	1SEZ/O24164	Oxidoreductase	**-8.6±0.0**
NRH dehydrogenase [quinone] 2	1SG0/P16083	Oxidoreductase	**-9.3±0.0**
Adenylosuccinate synthetase	1SON/P0A7D4	Ligase	**-8.4±0.0**
Pantothenate kinase	1SQ5/P0A6I3	Transferase	**-8.2±0.0**
Camp-dependent Protein Kinase A Type I	1STC/P00517	Complex (transferase/inhibitor)	**-8.4±0.0**
Atrial Natriuretic Peptide Receptor A,Natriuretic peptide receptor A	1T34/P18910	Signaling Protein	**-8.2±0.0**
Aldehyde Reductase	1T41/P15121	Oxidoreductase	**-9.6±0.0**
Kit tyrosine kinase	1T46/P10721	Transferase Activator	**-8.1±0.0**
Beta-galactosidase	1TG7/Q700S9	Hydrolase	**-8.6±0.0**
Tyrosine hydroxylase	1TOH/P04177	Hydroxylase	**-8.6±0.0**
Tubulin	1TUB/P02550	Microtubules	**-11.0±0.0**
Trypanothione Reductase	1TYP/P39040	Oxidoreductase	**-8.5±0.0**
Chalcone synthase 2	1U0W/P30074	Transferase	**-8.2±0.0**
Enoyl ACP reductase	1UH5/Q9BJJ9	Oxidoreductase	**-8.5±0.0**
Thrombin	1UVT/P00735	Serine Protease	**-8.3±0.0**
Adenosylhomocysteinase	1V8B/P50250	Hydrolase	**-8.6±0.0**
D-amino acid oxidase	1VE9/P00371	Oxidoreductase	**-9.4±0.0**
Beta-amylase	1VEM/P36924	Hydrolase	**-8.3±0.0**
Aldehyde oxidoreductase	1VLB/Q46509	Oxidoreductase	**-9.9±0.0**

Acetylcholinesterase	1VXR/P04058	Hydrolase	**-8.8±0.0**
Acyl CoA oxidase-1	1W07/O65202	Oxidoreductase	**-8.4±0.0**
Lanosterol Synthase	1W6K/P48449	Isomerase	**-9.5±0.0**
A-G adenine DNA glycosylase	1WEI/P17802	Hydrolase	**-8.2±0.0**
Transglycosylase	1WKD/P28720	TRNA Modifying Enzyme	**-8.2±0.0**
Isoleucyl tRNA synthetase	1WNY/P56690	Ligase	**-8.0±0.0**
Ras-Ras Gap	1WQ1/P01112	Complex (gtp Binding/gtpase Activation)	**-9.3±0.0**
Undecaprenyl diphosphate synthetase	1X07/P60472	Transferase	**-8.3±0.0**
hypothetical protein PA0115	1XEB/Q9I717	Transferase	**-8.1±0.0**
Epidermal Growth Factor Receptor(EGFR)	1XKK/P00533	Transferase	**-9.3±0.0**
Peroxisomal carnitine O-octanoyltransferase	1XMC/Q9DC50	Transferase	**-8.4±0.0**
cAMP-specific 3',5'-cyclic phosphodiesterase 4D	1XOQ/Q08499	Hydrolase	**-8.1±0.0**
cAMP-specific 3',5'-cyclic phosphodiesterase 4B	1XOS/Q07343	Hydrolase	**-8.2±0.0**
Glycerol kinase	1XUP/O34153	Transferase	**-8.1±0.0**
11-Hydroxysteroid dehydrogenase	1Y5M/P50172	Oxidoreductase	**-8.2±0.0**
Peptidyl dipeptidase Dcp	1Y79/P24171	Hydrolase	**-8.2±0.0**
Yeast Glycogen Phosphorylase	1YGP/P06738	Glycosyltransferase	**-8.3±0.0**
Voltage gated potassium channel	1ZSX/Q13303	Oxidoreductase	**-9.6±0.0**
AMP deaminase	2A3L/O80452	Hydrolase	**-8.5±0.0**
Acetylcholinesterase	2ACE/P04058	Serine Hydrolase	**-8.3±0.0**
Acetylcholinesterase	2ACK/P04058	Hydrolase	**-8.1±0.0**
Tropinone Reductase-II	2AE2/P50163	Oxidoreductase	**-9.2±0.0**
Methionine aminopeptidase 1	2B3K/P53582	Hydrolase	**-8.1±0.0**
Jak2 tyrosine kinase	2B7A/O60674	Transferase	**-8.1±0.0**
Glucose-6-phosphate dehydrogenase	2BH9/P11413	Oxidoreductase	**-8.4±0.0**
Mannosyltransferase	2BO6/Q9RFR0	Transferase	**-8.0±0.0**
Checkpoint kinase 1	2BRO/O14757	Transferase	**-8.2±0.0**
Pyruvate dehydrogenase kinase-2	2BU5/Q15119	Transferase	**-8.2±0.0**
Glutamate carboxypeptidase II	2C6C/Q04609	Hydrolase	**-9.9±0.0**
GMP reductase 2	2C6Q/Q9P2T1	Oxidoreductase	**-8.5±0.0**
Cytochrome P450 Reductase	2CPP/P00183	Oxidoreductase(oxygenase)	**-8.1±0.0**
Acyl CoA oxidase-2	2DDH/P07872	Oxidoreductase	**-8.8±0.0**
Cytochrome P450 2D6	2F9Q/P10635	Oxidoreductase	**-8.6±0.0**
UDP-N-Acetylmuramate dehydrogenase	2MBR/P08373	Oxidoreductase	**-9.0±0.0**
PARP	2PAW/P26446	Transferase	**-8.9±0.0**
P-Hydroxybenzoate Hydroxylase	2PHH/P00438	Oxidoreductase	**-8.3±0.0**
Endothiapepsin	3ER3/P11838	Hydrolase/hydrolase Inhibitor	**-8.0±0.0**
Reverse Transcriptase	3HVT/P03366	Nucleotidyltransferase	**-8.5±0.0**
Medium chain acyl CoA dehydrogenase	3MDE/P41367	Oxidoreductase	**-9.3±0.0**
Human Fibroblast Collagenase	4AYK/P03956	Matrix Metalloproteinase	**-8.5±0.0**
Citrate Synthase	4CTS/P00889	Oxo Acid Lyase	**-8.2±0.0**
Dihydrofolate Reductase	4DFR/P0ABQ4	Oxido Reductase	**-8.2±0.0**
Phenylalanine Hydroxylase	4PAH/P00439	Monooxygenase	**-8.0±0.0**
HIV-1 Protease	4PHV/P12497	Hydrolase(aspartic Proteinase)	**-8.1±0.0**
Thermolysin	4TMN/P00800	Hydrolase/hydrolase Inhibitor	**-8.0±0.0**

Cytochrome P450Cam	5CP4/P00183	Oxidoreductase	**-8.2±0.0**
Phospholipase A2	5P2P/P00592	Hydrolase(carboxylic Ester)	**-8.4±0.0**
Cyclooxygenase-2	6COX/Q05769	Oxidoreductase	**-9.4±0.0**
Cytochrome P450 Reductase	6CPP/P00183	Oxidoreductase(oxygenase)	**-8.2±0.0**
Cytochrome P450 Reductase	7CPP/P00183	Oxidoreductase(oxygenase)	**-8.1±0.0**
Catalase	8CAT/P00432	Oxidoreductase	**-8.4±0.0**
Cytochrome P450 Reductase	8CPP/P00183	Oxidoreductase(oxygenase)	**-8.3±0.0**
Circadian clock related proteins			
CK II alpha'	Oxidoreductase	2E3B/P19784	**-8.7±0.0**
CLK4	Transferase	2W96/P24385	**-8.0±0.0**
CLK1	Transferase	1Z57/P49759	**-9.2±0.0**
CLK2	Transferase	3NR9/P49760	**-8.3±0.0**
Insulin receptor pathway proteins			
PI3K-gamma	Serine/threonine protein kinase	1E8Y/P48736	**-8.7±0.0**
eIF-4E	Translation/protein binding	1WKW/P06730	**-8.2±0.0**
PKC-theta	Transferase	1XJD/Q04759	**-8.1±0.0**
MAPK 10	Transferase	2P33/P53779	**-8.1±0.0**
PKA C-alpha	Transferase/transferase inhibitor	2QCS/P05132	**-9.0±0.0**
PDE10A	Hydrolase	2WEY/Q9Y233	**-8.0±0.0**
AKT-2	Transferase	2X39/P31751	**-8.4±0.0**
FOXO1	Transcription/DNA	3CO6/Q12778	**-9.0±0.0**
iNOS	Oxidoreductase/metal Binding Protein	3HR4/P35228	**-8.5±0.0**
Serum proteins			
Topo1	Isomerase/DNA	1A35/P11387	**-9.0±0.0**
AE 1	Membrane protein	1HYN/P02730	**-8.0±0.0**
SL-2	Hydrolase	1Q3A/P09238	**-8.9±0.0**
ADAM 17	Hydrolase	2DDF/P78536	**-8.2±0.0**
ADAM-TS 1	Hydrolase	2JIH/Q9UHI8	**-8.4±0.0**
ADAM-TS 5	Hydrolase	3B8Z/Q9UNA0	**-8.3±0.0**
ADORA2A	Membrane protein , receptor	3EML/P29274	**-8.4±0.0**
MMP-13	Matrix metalloprotease	830C/P45452	**-8.3±0.0**
Breast cancer proteins			
MMP-9	Hydrolase	1GKC/P14780	**-8.6±0.0**
Stromelysin-1	Hydrolase	1HY7/P08254	**-8.0±0.0**
LAR	Hydrolase	1LAR/P10586	**-8.1±0.0**
MMP-16	Hydrolase	1RM8/P51512	**-8.0±0.0**
TOP2A	Isomerase	1ZXM/P11388	**-9.4±0.0**
CHEK2	Transferase	2W0J/O96017	**-8.1±0.0**
Beta-2 adrenoceptor	Membrane protein	3NY8/P07550	**-9.8±0.0**

Table S36. Results of the refinement docking experiments for trans-dityrosine with AutoDock Vina.

Binding Proteins			
Target name	PDB/UniProtKB	Type	Affinity (kcal/mol)
Annexin III	1AXN/P12429	Calcium/phospholipid Binding Protein	**-8.2±0.0**

Intestinal Fatty Acid Binding Protein	1ICN/P02693	Binding Protein(fatty Acid)	**-8.5±0.0**
Streptavidin	1SRJ/P22629	Biotin Binding Protein	**-8.7±0.0**
Uteroglobin	1UTR/P17559	Mammalian Pcb Binding Protein	**-8.2±0.0**
Intestinal Fatty Acid Binding Protein	2IFB/P02693	Fatty Acid Binding Protein	**-8.7±0.0**
		Nuclear Receptor	
EAR-1	1A6Y/P20393	Transcription/DNA	**-8.5±0.0**
Cellular Retinoic-Acid-Binding Protein Type II	1CBS/P29373	Retinoic Acid Transport	**-8.1±0.0**
Nuclear Vitamin D Receptor	1DB1/P11473	Gene Regulation	**-8.5±0.0**
Lac Repressor	1EFA/P03023	Transcription/dna	**-9.0±0.0**
EAR-1	1A6Y/P20393	Transcription/DNA	**-8.5±0.0**
Vitamin D3 Receptor	1IE9/P11473	Gene Regulation	**-8.8±0.0**
ERR-beta	1LO1/O95718	Hormone/growth factor receptor/DNA	**-7.9±0.0**
Nuclear receptor ROR-alpha	1N83/P35398	Lipid Binding Protein	**-8.4±0.0**
Thyroid hormone receptor Beta-1	1NAX/P10828	Membrane Protein	**-10.7±0.0**
Liver X receptor alpha	1UHL/P28702	DNA Binding Protein	**-8.1±0.0**
Retinoic acid receptor beta	1XAP/P10826	Transcription	**-8.1±0.0**
Orphan nuclear receptor NR5A2	1YUC/Q15466	Transcription Regulation	**-8.1±0.0**
Peroxisome proliferator activated receptor delta	2AWH/Q03181	Gene Regulation	**-8.6±0.0**
Peroxisome Proliferator Activated Receptor gamma	2F4B/P37231	Transcription Activator	**-8.1±0.0**
		Transport Proteins	
Flavodoxin	1BU5/P00323	Electron Transport	**-7.5±0.0**
Nonspecific lipid transfer protein	1CZ2/P24296	Lipid Binding Protein	**-8.4±0.0**
Retinol Binding Protein	1FEN/P18902	Transport Protein	**-8.1±0.0**
Plasma retinol-binding protein	1IIU/P41263	Transport Protein	**-8.5±0.0**
human retinol-binding protein	1QAB/P02753	Transport Protein	**-8.2±0.0**
Transferrin	1TFD/P19134	Iron Transport Protein	**-8.9±0.0**
Serum protein	2BX8/P02768	Transport Protein	**-8.1±0.0**
		Receptors	
Progesterone Receptor	1A28/P06401	Progesterone Receptor	**-8.0±0.0**
Retinoid X Receptor-alpha	1DKF/P10276	Hormone/growth Factor Receptor	**-9.0±0.0**
Peroxisome Proliferator Activated Receptor gamma (PPAR gamma)	1FM6/P37231	Transcription	**-8.2±0.0**
Glutama receptor subunit 2	1FTK/P19491	Membrane Protein	**-8.1±0.0**
androgen Receptor	1GS4/P10275	Androgen Receptor	**-8.0±0.0**
Metabotropic Glutamate Receptor 2	1P1N/P19491	Membrane Protein	**-9.5±0.0**
Phosphatidylinositol 3-kinase	1PIC/P27986	Complex (phosphotransferase/receptor)	**-8.4±0.0**
Retinoic acid receptor RXR-beta	1UHL/Q13133	DNA Binding Protein	**-8.1±0.0**
Oxysterols receptor LXR-beta	1UPV/P55055	Receptor	**-8.2±0.0**

Peroxisome Proliferator Activated Receptor delta(PPAR delta)	1Y0S/Q03181	Hormone/growth Factor Receptor	**-8.0±0.0**
Maltose-binding periplasmic protein	1YTV/P0AEX9	Sugar Binding Protein	**-8.5±0.0**
Monoclonal Antibodies			
Fab' Fragment Of Monoclonal Antibody Db3	1DBM/P01868	Immunoglobulin	**-8.9±0.0**
IGG2B-KAPPA 17E8 FAB	1EAP	Catalytic Antibody	**-8.2±0.0**
Type I IL-1 receptor	1G0Y/P14778	Immune System	**-8.1±0.0**
Immunoglobulin lambda Light Chain Dimer (Mcg)	1MCJ	Immunoglobulin	**-9.0±0.0**
Mhc Class I H-2Kb Heavy Chain	1VAC/Q7SIF6	Complex (mhc I/peptide)	**-8.3±0.0**
4-4-20 (IgG2A) Fab Fragment	4FAB/P01865	Immunoglobulin	**-8.5±0.0**
Factor, Regulator and Hormones			
Elongation Factor Tu	1HA3/Q5SHN6	Translation	**-8.6±0.0**
Heat Shock 70 kDa protein 8	1HX1/P19120	Chaperone/chaperone Inhibitor	**-8.8±0.0**
Elongation Factor 1-alpha	1IJE/P02994	Translation	**-8.1±0.0**
Imaginal Disc Growth Factor-2	1JND/Q9V3D4	Hormone/growth Factor	**-8.0±0.0**
Nuclear Orphan Receptor Lxr-beta	1K4W/P45446	Hormone/growth Factor	**-8.2±0.0**
Gonadotropin alpha subunit	1QFW/P01215	Immune System	**-8.5±0.0**
Structural Proteins			
Tubulin alpha chain	1JFF/P02550/P02554	Structural Protein	**-8.1±0.0**
Myosin light chain kinase family	2BKH/Q29122	Motor Protein/metal Binding Protein	**-9.4±0.0**
Signaling Proteins			
Neutrophil Cytosol Factor 2	1E96/P19878	Signaling Protein	**-8.6±0.0**
Ran-GPPNHP-RanBP1-RanGAP	1K5D/P62826 /P43487/P4 1391	Signaling Protein/signaling Activator	**-9.2±0.0**
Ion Channels			
Hypothetical Protein Slr1257	1II5/P73797	Membrane Protein	**-7.9±0.0**
Voltage-Gated Potassium Channel	1JVM/P0A334	Membrane Protein	**-8.5±0.0**
Lipid Binding Protein			
KES1 protein	1ZHY/P35844	Lipid Binding Protein	**-9.1±0.0**
Undefined receptors			
bilin binding protein (BBP)	1BBP/P09464	Bilin Binding	**-8.3±0.0**
Catabolite Gene Activator Protein	1J59/P0ACJ8	Gene Regulation/dna	**-8.0±0.0**
Actin, alpha skeletal muscle	1NWK/P68135	Contractile Protein	**-8.8±0.0**
Enzymes			
Alpha-thrombin	1A4W/P00734	Hydrolase/hydrolase Inhibitor	**-8.5±0.0**
Acetylcholinesterase	1ACJ/P04058	Hydrolase(carboxylic Esterase)	**-9.2±0.0**
Serine Proteinase alpha-thrombin	1AE8/P00734	Hydrolase/hydrolase Inhibitor	**-8.1±0.0**

Cytochrome C Peroxidase	1AEV/P00431	Oxidoreductase	**-8.2±0.0**
Glycolate Oxidase	1AL8/P05414	Flavoprotein	**-7.9±0.0**
Pepsin	1AM5/P56272	Aspartyl Protease	**-7.5±0.0**
TGF-beta receptor type I	1B6C/P36897	Complex (isomerase/protein Kinase)	**-8.4±0.0**
17-beta Hydroxysteroid-Dehydrogenase Type 1,Estradiol 17 beta dehydrogenase	1BHS/P14061	Oxidoreductase	**-8.3±0.0**
Fructose biphosphatase	1BIF/P25114	Bifunctional Enzyme	**-9.5±0.0**
Map Kinase P38	1BMK/Q16539	Transferase	**-8.4±0.0**
Serine Proteinase alpha-thrombin	1BMN/P00734	Hydrolase/hydrolase Inhibitor	**-8.5±0.0**
Cytosine DNA methyltransferase	1BOO/P11409	Transferase	**-7.8±0.0**
Liver Alcohol Dehydrogenase	1BTO/P00327	Oxidoreductase	**-8.3±0.0**
Protein (Cytochrome P450 BM-3)	1BVY/P14779	Oxidoreductase	**-8.3±0.0**
NADPH Dehydrogenase 1	1BWK/Q02899	Oxidoreductase	**-8.7±0.0**
Beta-Amylase	1BYB/P10538	Hydrolase(o Glycosyl)	**-8.5±0.0**
Dihydrofolate Reductase	1BZF/P00381	Oxidoreductase	**-8.2±0.0**
Neutrophil Collagenase	1BZS/P22894	Hydrolase	**-8.9±0.0**
Ras-Binding Domain of the Serine/Threonine Kinase C-Raf1	1C1Y/P62834	Signaling Protein	**-8.8±0.0**
Lumazine Synthase	1C41/Q9UVT8	Transferase	**-9.3±0.0**
Ornithine Decarboxylase	1C4K/P43099	Lyase	**-7.9±0.0**
Aspartate Aminotransferase, Cytoplasmic	1C9C/P00509	Transferase	**-8.3±0.0**
Glutamate Mutase	1CB7/P80078	Isomerase	**-8.6±0.0**
Cobalt-Precorrin-4 Transmethylase	1CBF/O87696	Methyltransferase	**-8.4±0.0**
Pnp Oxidase	1CI0/P38075	Oxidoreductase	**-8.2±0.0**
Thymidylate Synthase	1CI7/P13100	Transferase	**-8.2±0.0**
MRNA Capping Enzyme	1CKN/Q84424	Capping Enzyme	**-8.3±0.0**
Cholesterol Oxidase	1COY/P22637	Oxidoreductase(oxygen Receptor)	**-8.9±0.0**
Cytochrome P450-Terp	1CPT/P33006	Oxidoreductase(oxygenase)	**-8.5±0.0**
Epoxide hydrolase	1CQZ/P34914	Hydrolase	**-8.6±0.0**
Casein kinase I	1CSN/P40233	Phosphotransferase	**-8.0±0.0**
Cyclooxygenase-2	1CX2/Q05769	Oxidoreductase	**-9.0±0.0**
Myeloperoxidase	1D2V/P05164	Oxidoreductase	**-8.2±0.0**
Serine Proteinase alpha-thrombin	1D4P/P00734	Hydrolase/hydrolase Inhibitor	**-8.2±0.0**
Hypoxanthine-Guanine Phosphoribosyltransferase	1D6N/P00492	Transferase	**-9.4±0.0**
O-acetylserine Sulfhydrylase	1D6S/P0A1E3	Lyase	**-8.5±0.0**
Cytosolic Phospholipase A2	1DB4/P14555	Hydrolase/hydrolase Inhibitor	**-8.0±0.0**
Dihydrofolate Reductase	1DF7/P9WNX1	Oxidoreductase	**-8.8±0.0**
Dihydrofolate Reductase	1DHF/P00374	Oxido Reductase	**-8.5±0.0**
Dihydrofolate Reductase	1DHJ/P0ABQ4	Oxidoreductase	**-9.0±0.0**

Estrogenic 17-Beta Hydroxysteroid Dehydrogenase	1DHT/P14061	Oxidoreductase	**-8.2±0.0**
Cyclin-Dependent Kinase 2 (Cdk2)	1DI8/P24941	Transferase	**-8.5±0.0**
D-Xylose Isomerase	1DID/P12070	Isomerase(intramolecular Oxidoreductase)	**-8.1±0.0**
Dihydrofolate Reductase	1DIS/P00381	Oxido Reductase	**-9.3±0.0**
Prostaglandin H2 Synthase-1	1DIY/P05979	Oxidoreductase	**-8.0±0.0**
Phenylalanine Hydroxylase	1DMW/P00439	Oxidoreductase	**-8.6±0.0**
Dihydrofolate Reductase	1DRF/P00374	Oxidoreductase (ch Nh(d) NAD or Nadp(a))	**-8.6±0.0**
Serine Proteinase alpha-thrombin	1DWC/P00734	Hydrolase/hydrolase Inhibitor	**-8.0±0.0**
Serine Proteinase alpha-thrombin	1DWD/P00734	Hydrolase/hydrolase Inhibitor	**-8.2±0.0**
beta-Glucosidase	1E1F/P49235	Hydrolase	**-9.9±0.0**
GDP fucose synthetase	1E6U/P32055	Epimerase/reductase	**-8.4±0.0**
GDP-Fucose Synthetase	1E7S/P32055	Epimerase/reductase	**-8.3±0.0**
Pteridine Reductase	1E7W/Q01782	Oxidoreductase	**-8.2±0.0**
Nitric Oxide Synthase	1ED5/P29473	Oxidoreductase	**-8.3±0.0**
HIV-1 Reverse Transcriptase	1EET/P03366	Viral Protein	**-8.9±0.0**
Hydroxysteroid Sulfotransferase	1EFH/Q06520	Transferase	**-8.2±0.0**
Bovine Mitochondrial F1-ATPase	1EFR/P19483/P00829/P05631	Hydrolase/antibiotic	**-9.6±0.0**
DNA Gyrase B Subunit	1EI1/P0AES6	Isomerase	**-8.5±0.0**
Prostaglandin H2 Synthase-1, COX-1	1EQH/P05979	Oxidoreductase	**-8.5±0.0**
Epsilon- Thrombin	1ETS/P00735	Hydrolase/hydrolase Inhibitor	**-8.1±0.0**
D-Amino acid oxidase	1EVI/P00371	Oxidoreductase	**-9.6±0.0**
Lipoxygenas-1	1F8N/P08170	Oxidoreductase	**-8.4±0.0**
Factor XIII	1FIE/P00488	Transferase	**-8.1±0.0**
Carboxylase	1FIY/P00864	Complex (lyase/inhibitor)	**-9.4±0.0**
Memapsin 2 (Beta-Secretase)	1FKN/P56817	Hydrolase/hydrolase Inhibitor	**-8.2±0.0**
Peptidase T	1FNO/P26311	Hydrolase	**-8.3±0.0**
Xanthine dehydrogenase	1FO4/P80457	Oxidoreductase	**-9.3±0.0**
Glucose phosphate thymidylyltransferase	1FXO/Q9HU22	Transferase	**-8.6±0.0**
Glucosidase-471	1GAI/ P69327	Hydrolase	**-8.4±0.0**
Cell Division Protein Kinase 2(CDK2)	1GIH/P24941	Transferase	**-9.1±0.0**
Cyclin Dependent Kinase 2(Cdk2)	1GII/P24941	Transferase	**-8.1±0.0**
Monoamine Oxidase	1GOS/P27338	Oxidoreductase	**-9.4±0.0**
Spinach glycolate oxidase	1GOX/P05414	Oxidoreductase (oxygen(a))	**-9.4±0.0**
Glycogen Phosphorylase B	1GPB/P00489	Glycogen Phosphorylase	**-8.9±0.0**
Acetylcholinesterase	1GPK/P04058	Hydrolase	**-8.5±0.0**
GMP synthetase	1GPM/P04079	Transferase (glutamine Amidotransferase)	**-8.7±0.0**
Acetylcholinesterase	1GPN/P04058	Hydrolase	**-8.9±0.0**
Glutathione reductase	1GRE/P00390	Oxidoreductase	**-8.2±0.0**
Glutathione Transferase A1-1	1GSF/P08263	Transferase (glutathione)	**-8.8±0.0**

Thymidine Kinase	1GSI/P9WKE1	Transferase	**-8.9±0.0**
Cyclin Dependent Kinase 2(Cdk2)	1GZ8/P24941	Transferase	**-8.1±0.0**
Nitrogenase Molybdenum Iron Protein Alpha Chain	1H1L/P00466	Oxidoreductase	**-8.3±0.0**
Glycogen phosphorylase	1H5U/P00489	Glycogen Metabolism	**-8.5±0.0**
Dihydropyrimidine dehydrogenase	1H7X/Q28943	Electron Transfer	**-8.4±0.0**
Polyamine oxidase	1H82/O64411	Oxidoreductase	**-8.8±0.0**
Elongation Factor Tu	1HA3/Q5SHN6	Translation	**-8.6±0.0**
Isopenicillin N Synthase	1HB2/P05326	Antibiotic Biosynthesis	**-8.2±0.0**
3-alpha-hydroxysteroid Dehydrogenase	1HDC/P19992	Oxidoreductase	**-8.1±0.0**
Biliverdin Ix Beta Reductase	1HDO/P30043	Biliverdin Ix Beta Reductase	**-7.7±0.0**
Serine Proteinase alpha-thrombin	1HDT/P00734	Hydrolase/hydrolase Inhibitor	**-8.3±0.0**
Alcohol Dehydrogenase	1HLD/P00327	Oxidoreductase(ch Oh(d) Nad(a))	**-8.2±0.0**
Prophospholipase A2	1HN4/P00592	Hydrolase	**-9.0±0.0**
Pyridoxine 5'-Phosphate Synthase	1HO4/P0A794	Biosynthetic Protein	**-8.5±0.0**
Adenosinetriphosphatase	1HPM/P19120	Hydrolase (acting On Acid Anhydrides)	**-9.5±0.0**
HIV-1 Protease	1HPS/P03366	Hydrolase(acid Proteinase)	**-8.2±0.0**
Leukotriene A4 hydrolase	1HS6/P09960	Hydrolase	**-8.2±0.0**
D-alanyl-D-alanine Carboxypeptidase	1HVB/P15555	Hydrolase	**-8.1±0.0**
Neutrophil Collagenase, MMP8	1I76/P22894	Hydrolase	**-8.6±0.0**
Methionine decarboxylase	1I7B/P17707	Lyase	**-9.3±0.0**
Riboflavin Synthase	1I8D/P0AFU8	Transferase	**-8.3±0.0**
Nitroreductase	1ICR/P38489	Oxidoreductase	**-8.1±0.0**
Thiamin Pyrophosphokinase	1IG0/P35202	Transferase	**-8.3±0.0**
Thiamin Pyrophosphokinase	1IG3/Q9R0M5	Transferase	**-8.7±0.0**
Cholesterol Oxidase	1IJH/P12676	Oxidoreductase	**-9.1±0.0**
Inositol monophosphatase	1IMB/P29218	Hydrolase	**-8.0±0.0**
Branched Chain Amino Acid-dependent Aminotransferase	1IYD/P0AB80	Transferase	**-7.4±0.0**
Aldo-Keto Reductase Family 1 Member C3	1J96/P52895	Oxidoreductase	**-8.8±0.0**
Camp-Dependent Protein Kinase, Alpha-Catalytic Subunit	1JBP/P05132	Transferase	**-9.2±0.0**
Cyclic nucleotide phosphodiesterase	1JH7/O04147	Hydrolase	**-8.3±0.0**
HIV Protease	1JKH/P04585	Transferase	**-8.5±0.0**
cAMP-dependent Protein Kinase	1JLU/P05132	Transferase/transferase Inhibitor	**-8.3±0.0**
Jun N terminal kinase	1JNK/P53779	Transferase	**-8.4±0.0**
NADH Peroxidase	1JOA/P37062	Oxidoreductase	**-8.0±0.0**
Methylthioadenosine phosphorylase	1JP7/P50389	Transferase	**-8.7±0.0**
Phospholipase A2	1JQ9/P59071	Hydrolase/hydrolase Inhibitor	**-8.5±0.0**

Histamine N-methyltransferase	1JQE/P50135	Transferase	**-9.2±0.0**
Inosine-5'-monophosphate Dehydrogenase 2,IMP-2 dehydrogenase	1JR1/P12269	Oxidoreductase	**-8.6±0.0**
Aromatic-L-amino-acid Decarboxylase	1JS3/P80041	Lyase	**-9.1±0.0**
Dopa decarboxylase	1JS6/P80041	Lyase	**-8.7±0.0**
Casein Kinase II, Alpha Chain	1JWH/P68400	Transferase	**-8.1±0.0**
Prothrombin	1JWT/P00734	Hydrolase	**-8.3±0.0**
Glutathione Transferase A1-1	1K3Y/P08263	Transferase	**-8.7±0.0**
Nicotinate-nucleotide Adenylyltransferase	1K4M/P0A752	Transferase	**-8.3±0.0**
Cytosine deaminase	1K6W/P25524	Hydrolase	**-8.3±0.0**
Triacylglycerol Lipase, Pancreatic	1K8Q/P80035	Hydrolase	**-8.1±0.0**
Transforming Protein P21/H-Ras-1	1K8R/P01112	Signaling Protein	**-8.7±0.0**
Histidinol Dehydrogenase	1KAE/P06988	Oxidoreductase	**-8.2±0.0**
Neutrophil Collagenase	1KBC/P22894	Metalloproteinase	**-8.8±0.0**
Mycolic Acid Synthase	1L1E/P9WPB3	Transferase	**-8.2±0.0**
Inositol 1 phosphatase	1LBV/O30298	Hydrolase	**-8.2±0.0**
Alcohol Dehydrogenase	1LDE/P00327	Dehydrogenase	**-8.3±0.0**
L-lactate Dehydrogenase	1LDM/P00341	Oxidoreductase	**-8.6±0.0**
Casein Kinase II, Protein Kinase Ck2	1LP4/P28523	Transferase	**-9.7±0.0**
Guanylate kinase	1LVG/Q64520	Transferase	**-9.0±0.0**
Memapsin 2 (Beta-Secretase)	1M4H/P56817	Hydrolase/hydrolase Inhibitor	**-8.4±0.0**
Nitric Oxide Synthase	1M9M/P29474	Oxidoreductase	**-8.4±0.0**
Inosine-5'-Monophosphate Dehydrogenase	1ME8/P50097	Oxidoreductase	**-8.2±0.0**
Phenylethanolamine N-Methyltransferase	1N7I/P11086	Transferase	**-6.8±0.0**
Carnitine Acetyltransferase	1NDF/P47934	Transferase	**-8.0±0.0**
Carnitine acyltransferase	1NM8/P43155	Transferase	**-8.0±0.0**
3-dehydroquinate Synthase	1NR5/P07547	Lyase	**-8.5±0.0**
Activated Akt/Protein Kinase B	1O6L/P31751	Transferase	**-8.3±0.0**
Alpha mannosidase	1O7D/Q29451	Hydrolase	**-8.1±0.0**
Fibroblast Growth Factor Receptor 2	1OEC/P21802	Transferase	**-7.9±0.0**
Cytochrome P450 2C9	1OG5/P11712	Electron Transport	**-8.0±0.0**
Cell Division Protein Kinase 2	1OI9/P24941	Kinase	**-8.7±0.0**
Cell Division Protein Kinase 2	1OIU/P24941	Kinase	**-8.9±0.0**
Carbonic Anhydrase II	1OKL/P00918	Lyase	**-8.4±0.0**
Dihydroorotate Dehydrogenase, Mitochondrial	1OVD/A2RJT9	Oxidoreductase	**-7.9±0.0**
NADPH Dehydrogenase	1OYA/Q02899	Oxidoreductase (flavoprotein)	**-9.1±0.0**

Glycogen Phosphorylase, Muscle Form	1P2D/P00489	Transferase	**-8.8±0.0**
Riboflavin Kinase	1P4M/Q969G6	Transferase	**-9.3±0.0**
P-Hydroxybenzoate Hydroxylase	1PBD/P00438	Oxidoreductase	**-8.9±0.0**
Coagulation Factor IX	1PFX/P16293	Hydrolase/hydrolase Inhibitor	**-8.0±0.0**
Cytochrome P450-Cam	1PHD/ P00183	Oxidoreductase(oxygenase)	**-8.1±0.0**
Phosphatidylinositol 3-kinase	1PIC/P27986	Complex (phosphotransferase/receptor)	**-8.2±0.0**
N, N-Dimethylglycine Oxidase	1PJ6/Q9AGP8	Oxidoreductase	**-9.6±0.0**
Casein Kinase II, Alpha Chain	1PJK/P68400	Transferase	**-8.8±0.0**
Phospholipase A2	1POC/P00630	Hydrolase	**-8.1±0.0**
alpha-Amylase	1PPI/P00690	Hydrolase (o Glycosyl)	**-8.2±0.0**
Cytochrome P450 2C8	1PQ2/P10632	Oxidoreductase	**-8.1±0.0**
2,4-Dienoyl-CoA Reductase	1PS9/P42593	Oxidoreductase	**-8.4±0.0**
DNA Topoisomerase II	1PVG/P06786	Isomerase	**-9.4±0.0**
Prostaglandin G/H Synthase 2	1PXX/Q05769	Oxidoreductase	**-9.1±0.0**
TGF-beta receptor type I	1PY5/P36897	Transferase	**-8.6±0.0**
Penicillin Binding Protein 2	1PYY/P59676	Transpeptidase	**-7.9±0.0**
2-amino-4-hydroxy-6-hydroxymethyldihydropteridine pyrophosphokinase	1Q0N/P26281	Transferase	**-9.0±0.0**
Intestinal Glcnac-6-sulfotransferase	1Q1Z/O00204	Transferase	**-8.9±0.0**
Prostaglandin-E2 9-reductase	1Q5M/P80508	Oxidoreductase	**-9.9±0.0**
3-hydroxy-3-methylglutaryl-coenzyme A Reductase	1QAX/P13702	Oxidoreductase	**-8.3±0.0**
Phospholipase A1	1QD6/P0A921	Membrane Protein	**-9.7±0.0**
Stromelysin	1QIA/P08254	Hydrolase	**-8.2±0.0**
Acetylcholinesterase	1QIJ/P04058	Hydrolase	**-8.6±0.0**
Isopenicillin N Synthetase	1QIQ/P05326	Antibiotic Biosynthesis	**-8.4±0.0**
Pyruvate Decarboxylase	1QPB/P06169	Lyase	**-9.8±0.0**
LCK Tyrosine Kinase	1QPJ/P06239	Transferase	**-7.8±0.0**
Ornithine Decarboxylase	1QU4/P07805	Lyase	**-8.0±0.0**
Hepatocyte Growth Factor Receptor	1R0P/P08581	Transferase	**-7.6±0.0**
Methyltransferase	1R18/Q27869	Transferase	**-8.0±0.0**
Cytochrome P450 2C9	1R9O/P11712	Oxidoreductase	**-8.5±0.0**
Methylmalonyl-coa Mutase	1REQ/P11653	Isomerase	**-8.9±0.0**
Renin	1RNE/ P00797	Hydrolase(acid Proteinase)	**-9.2±0.0**
HIV-1 Reverse Transcriptase	1RT6/P04585	Nucleotidyltransferase	**-8.8±0.0**
Mitogen-Activated Protein Kinase Kinase 2 (MEK2)	1S9I/P36507	Transferase	**-8.2±0.0**
Protoporphyrinogen oxidase	1SEZ/O24164	Oxidoreductase	**-9.7±0.0**
4-aminobutyrate aminotransferase	1SFF/P22256	Transferase	**-8.5±0.0**
NRH dehydrogenase [quinone] 2	1SG0/P16083	Oxidoreductase	**-8.8±0.0**
Thermonuclease Precursor	1SNC/P00644	Hydrolase (phosphoric Diester)	**-7.8±0.0**

Adenylosuccinate synthetase	1SON/P0A7D4	Ligase	**-9.0±0.0**
Pantothenate kinase	1SQ5/P0A6I3	Transferase	**-8.0±0.0**
Camp-dependent Protein Kinase A Type I	1STC/P00517	Complex (transferase/inhibitor)	**-8.5±0.0**
Atrial Natriuretic Peptide Receptor A,Natriuretic peptide receptor A	1T34/P18910	Signaling Protein	**-8.1±0.0**
Aldehyde Reductase	1T41/P15121	Oxidoreductase	**-9.5±0.0**
Kit tyrosine kinase	1T46/P10721	Transferase Activator	**-8.6±0.0**
Beta-galactosidase	1TG7/Q700S9	Hydrolase	**-8.8±0.0**
Thymidylate Synthase	1TIS/P00471	Transferase(methyltransferase)	**-8.0±0.0**
Tyrosine hydroxylase	1TOH/P04177	Hydroxylase	**-8.7±0.0**
Beta-Secretase	1TQF/P56817	Hydrolase	**-8.2±0.0**
Thymidylate Synthetase	1TSD/P0A884	Transferase (methyltransferase)	**-8.1±0.0**
Tubulin	1TUB/P02550	Microtubules	**-11.2±0.0**
Reverse Transcriptase	1TVR/P03366	Aspartyl Protease	**-8.4±0.0**
P50-Rhogap	1TX4/Q07960	Complex (gtpase Activatn/proto Oncogene)	**-8.2±0.0**
Trypanothione Reductase	1TYP/P39040	Oxidoreductase	**-8.5±0.0**
Chalcone synthase 2	1U0W/P30074	Transferase	**-7.7±0.0**
Metalloprotease 3	1UEA/P08254	Complex (metalloprotease/inhibitor)	**-8.4±0.0**
Enoyl ACP reductase	1UH5/Q9BJJ9	Oxidoreductase	**-8.7±0.0**
Uridylmonophosphate/Cytidyl monophosphate Kinase	1UKE/P20425	Nucleotide Monophosphate Kinase	**-8.2±0.0**
Purine Nucleoside Phosphorylase	1ULB/P00491	Pentosyltransferase	**-8.1±0.0**
Aldose Reductase	1US0/P15121	Oxidoreductase	**-9.1±0.0**
Thrombin	1UVT/P00735	Serine Protease	**-8.3±0.0**
Adenosylhomocysteinase	1V8B/P50250	Hydrolase	**-8.8±0.0**
Acylamino-acid-releasing enzyme	1VE6/Q9YBQ2	Hydrolase	**-8.6±0.0**
D-amino acid oxidase	1VE9/P00371	Oxidoreductase	**-9.6±0.0**
Beta-amylase	1VEM/P36924	Hydrolase	**-8.3±0.0**
Alpha glucosidase	1VJT/Q9WZL1	Hydrolase	**-8.2±0.0**
Histone deacetylase-8	1VKG/Q9BY41	Hydrolase	**-8.0±0.0**
Acetylcholinesterase	1VOT/P04058	Hydrolase	**-8.6±0.0**
Acetylcholinesterase	1VXR/P04058	Hydrolase	**-8.9±0.0**
Acyl CoA oxidase-1	1W07/O65202	Oxidoreductase	**-8.5±0.0**
Beta-secretase-1	1W50/P56817	Hydrolase	**-6.9±0.0**
Lanosterol Synthase	1W6K/P48449	Isomerase	**-10.1±0.0**
A-G adenine DNA glycosylase	1WEI/P17802	Hydrolase	**-8.3±0.0**
Transglycosylase	1WKD/P28720	TRNA Modifying Enzyme	**-8.0±0.0**
Isoleucyl tRNA synthetase	1WNY/P56690	Ligase	**-8.2±0.0**
Ras-Ras Gap	1WQ1/P01112	Complex (gtp Binding/gtpase Activation)	**-9.1±0.0**
Undecaprenyl diphosphate synthetase	1X07/P60472	Transferase	**-8.3±0.0**
hypothetical protein PA0115	1XEB/Q9I717	Transferase	**-8.3±0.0**
D-Xylose Isomerase	1XID/P24300	Isomerase(intramolecular Oxidoreductase)	**-8.3±0.0**
Epidermal Growth Factor Receptor(EGFR)	1XKK/P00533	Transferase	**-9.4±0.0**

Peroxisomal carnitine O-octanoyltransferase	1XMC/Q9DC50	Transferase	**-8.0±0.0**
Thrombin light chain	1XMN/P00734	Hydrolase/hydrolase Inhibitor	**-8.1±0.0**
cAMP-specific 3',5'-cyclic phosphodiesterase 4D	1XOQ/Q08499	Hydrolase	**-8.4±0.0**
cAMP-specific 3',5'-cyclic phosphodiesterase 4B	1XOS/Q07343	Hydrolase	**-8.4±0.0**
3-hydroxy-3-methylglutaryl CoA synthase	1XPM/Q79ZY6	Transferase	**-8.5±0.0**
11-Hydroxysteroid dehydrogenase	1Y5M/P50172	Oxidoreductase	**-8.4±0.0**
Peptidyl dipeptidase Dcp	1Y79/P24171	Hydrolase	**-8.7±0.0**
Proto-oncogene tyrosine-protein kinase Src	1YOL/P12931	Transferase	**-8.0±0.0**
CLK1 protein kinase	1Z57/P49759	Transferase	**-8.9±0.0**
Histone lysine methyltransferase	1ZKK/Q9NQR1	Transferase	**-8.1±0.0**
Voltage gated potassium channel	1ZSX/Q13303	Oxidoreductase	**-9.8±0.0**
AMP deaminase	2A3L/O80452	Hydrolase	**-8.4±0.0**
WASP interacting protein	2A3Z/P68135	Structural Protein	**-9.2±0.0**
Acetylcholinesterase	2ACE/P04058	Serine Hydrolase	**-8.6±0.0**
Acetylcholinesterase	2ACK/P04058	Hydrolase	**-8.0±0.0**
Tropinone Reductase-II	2AE2/P50163	Oxidoreductase	**-9.0±0.0**
Adamalysin II	2AIG/P34179	Hydrolast/hydrolase Inhibitor	**-8.4±0.0**
Methionine aminopeptidase 1	2B3K/P53582	Hydrolase	**-8.1±0.0**
Jak2 tyrosine kinase	2B7A/O60674	Transferase	**-8.1±0.0**
Cholesterol esterase	2BCE/P30122	Hydrolase	**-8.9±0.0**
Glucose-6-phosphate dehydrogenase	2BH9/P11413	Oxidoreductase	**-8.5±0.0**
Checkpoint kinase 1	2BRO/O14757	Transferase	**-8.2±0.0**
Pyruvate dehydrogenase kinase-2	2BU5/Q15119	Transferase	**-8.2±0.0**
Glutamate carboxypeptidase II	2C6C/Q04609	Hydrolase	**-9.7±0.0**
GMP reductase 2	2C6Q/Q9P2T1	Oxidoreductase	**-8.6±0.0**
Other phosphate group transferase	2CG5/Q9NRN7	Transferase/hydrolase	**-8.0±0.0**
Cytochrome P450 Reductase	2CPP/P00183	Oxidoreductase(oxygenase)	**-8.1±0.0**
Acyl CoA oxidase-2	2DDH/P07872	Oxidoreductase	**-8.4±0.0**
Cytochrome P450 2D6	2F9Q/P10635	Oxidoreductase	**-9.1±0.0**
UDP-N-Acetylmuramate dehydrogenase	2MBR/P08373	Oxidoreductase	**-9.3±0.0**
PARP	2PAW/P26446	Transferase	**-8.8±0.0**
P-Hydroxybenzoate Hydroxylase	2PHH/P00438	Oxidoreductase	**-8.8±0.0**
Neuraminidase	2SIM/P29768	Hydrolase	**-8.1±0.0**
HIV-1 Protease	2UPJ/P03367	Hydrolase (acid Protease)	**-8.2±0.0**
Carbonic anhydrase-IV	2ZNC/Q64444	Lyase	**-7.8±0.0**
Alcohol Dehydrogenase	3BTO/P00327	Oxidoreductase	**-8.2±0.0**
Heat shock protein 70	3HSC/P19120	Hydrolase (acting On Acid Anhydrides)	**-8.5±0.0**

Reverse Transcriptase	3HVT/P03366	Nucleotidyltransferase	**-8.7±0.0**
Medium chain acyl CoA dehydrogenase	3MDE/P41367	Oxidoreductase	**-9.3±0.0**
Phosphoglycerate kinase	3PGK/P00560	Transferase	**-8.5±0.0**
Human Fibroblast Collagenase	4AYK/P03956	Matrix Metalloproteinase	**-8.4±0.0**
Phospholipase A2	4BP2/P00593	Carboxylic Ester Hydrolase Zymogen	**-8.1±0.0**
Citrate Synthase	4CTS/P00889	Oxo Acid Lyase	**-8.1±0.0**
Dihydrofolate Reductase	4DFR/P0ABQ4	Oxido Reductase	**-8.6±0.0**
Phenylalanine Hydroxylase	4PAH/P00439	Monooxygenase	**-8.6±0.0**
HIV-1 Protease	4PHV/P12497	Hydrolase(aspartic Proteinase)	**-8.3±0.0**
Cytochrome P450Cam	5CP4/P00183	Oxidoreductase	**-8.4±0.0**
Phospholipase A2	5P2P/P00592	Hydrolase(carboxylic Ester)	**-8.8±0.0**
Cyclooxygenase-2	6COX/Q05769	Oxidoreductase	**-9.1±0.0**
Cytochrome P450 Reductase	6CPP/P00183	Oxidoreductase(oxygenase)	**-8.0±0.0**
Cytochrome P450 Reductase	7CPP/P00183	Oxidoreductase(oxygenase)	**-8.1±0.0**
Catalase	8CAT/P00432	Oxidoreductase	**-8.4±0.0**
Cytochrome P450 Reductase	8CPP/P00183	Oxidoreductase(oxygenase)	**-8.2±0.0**
Circadian clock related proteins			
CK II alpha'	Oxidoreductase	2E3B/P19784	**-8.8±0.0**
CLK1	Transferase	2VAG/P49759	**-8.5±0.0**
CLK4	Transferase	2W96/P24385	**-8.1±0.0**
CK II alpha	Transferase	3H30/P68400	**-8.0±0.0**
CLK2	Transferase	3NR9/P49760	**-8.2±0.0**
Insulin receptor pathway proteins			
PI3K-gamma	Serine/threonine protein kinase	1E8Y/P48736	**-8.4±0.0**
GSK3B	Transferase	1UV5/P49841	**-8.0±0.0**
RHEB	Signaling protein	1XTS/Q15382	**-8.6±0.0**
MAPK 10	Transferase	2P33/P53779	**-8.3±0.0**
PKA C-alpha	Transferase/transferase inhibitor	2QCS/P05132	**-9.5±0.0**
AMPK subunit beta-2	Transferase	2V8Q/O43741	**-8.0±0.0**
AKT-2	Transferase	2X39/P31751	**-8.7±0.0**
P70S6K1	Transferase	3A62/P23443	**-8.0±0.0**
FOXO1	Transcription/DNA	3CO6/Q12778	**7.9±0.0**
SGK1	Transferase	3HDN/O00141	**-8.7±0.0**
iNOS	Oxidoreductase/metal Binding Protein	3HR4/P35228	**-8.6±0.0**
Serum proteins			
Topo1	Isomerase/DNA	1A35/P11387	**-8.7±0.0**
AE 1	Membrane protein	1HYN/P02730	**-8.1±0.0**
HLA-B8	Immune system	1M05/P30460	**-8.2±0.0**
SL-2	Hydrolase	1Q3A/P09238	**-9.2±0.0**
HLA-A	Immune system	1QVO/P13746	**-8.3±0.0**
ADAM 17	Hydrolase	2DDF/P78536	**-8.3±0.0**
ADAM-TS 1	Hydrolase	2JIH/Q9UHI8	**-8.6±0.0**
ADAM-TS 4	Hydrolase	2RJP/O75173	**-8.9±0.0**
ADAM-TS 5	Hydrolase	3B8Z/Q9UNA0	**-8.8±0.0**

ADORA2A	Membrane protein , receptor	3EML/P29274	**-8.4±0.0**
ADAM 22	Membrane protein	3G5C/Q9P0K1	**-8.4±0.0**
MMP-13	Matrix metalloprotease	830C/P45452	**-8.3±0.0**
	Breast cancer proteins		
MMP-9	Hydrolase	1GKC/P14780	**-8.8±0.0**
MMP-16	Hydrolase	1RM8/P51512	**-8.8±0.0**
DNA topoisomerase II	Isomerase	1ZXM/P11388	**-9.2±0.0**
CHEK2	Transferase	2W0J/O96017	**-8.3±0.0**
Beta-2 adrenoceptor	Membrane protein	3NY8/P07550	**-9.2±0.0**

Table 37. Results of the validation experiments for cis-dityrosine with AutoDock Vina.

Target name	PDB/UniProtKB	Affinity (kcal/mol)
Binding Proteins		
Intestinal Fatty Acid Binding Protein (IFABP)	1ICN/P02693	**-9.1±0.0(-7.2)**
Nuclear Receptors		
Lac Repressor (LacR)	1EFA/P03023	**-9.0±0.0(-9.0)**
Undefined receptors		
Actin, alpha skeletal muscle (Actin)	1NWK/P68135	**-9.0±0.0(-10.8)**
Enzymes		
Fructose biphosphatase (FBP)	1BIF/P25114	**-9.6±0.0(-9.6)**
Dihydroorotate Dehydrogenase, Mitochondrial (DHOD)	1D3G/Q02127	**-9.4±0.0(-12.6)**
Hypoxanthine-Guanine Phosphoribosyltransferase (HPRT)	1D6N/P00492	**-9.6±0.0(-8.3)**
Dihydrofolate Reductase (DHFR)	1DIS/P00381	**-9.2±0.0(-8.6)**
beta-Glucosidase (β-GS)	1E1F/P49235	**-9.6±0.0(-9.1)**
Bovine Mitochondrial F1-ATPase (BMF1)	1EFR/P19483	**-9.6±0.0(-9.7)**
Carboxylase	1FIY/P00864	**-9.3±0.0(-5.2)**
Monoamine Oxidase (MAO)	1GOS/P27338	**-9.4±0.0(-10.1)**
Glycogen Phosphorylase B (GPB)	1GPB/P00489	**-9.1±0.0(-7.3)**
Acetylcholinesterase	1GQS/P04058	**-9.0±0.0(-6.7)**
Polyamine oxidase (PAO)	1H82/O64411	**-9.3±0.0(-6.5)**
Prophospholipase A2 (proPLA2)	1HN4/P00592	**-9.4±0.0(-7.5)**
Cholesterol Oxidase (ChOx)	1IJH/P12676	**-9.0±0.0(-13.1)**
Aldo-Keto Reductase Family 1 Member C3 (AKR1C3)	1J96/P52895	**-9.0±0.0(-10.9)**
Casein Kinase II, Protein Kinase Ck2 (CK2)	1LP4/P28523	**-9.5±0.0(-7.9)**
NADPH Dehydrogenase (NDH)	1OYA/Q02899	**-9.1±0.0(-13.2)**
DNA Topoisomerase II (topo II)	1PVG/P06786	**-9.3±0.0(-10.8)**

Prostaglandin G/H Synthase 2	1PXX/Q05769	**-9.2±0.0(-9.2)**
2-amino-4-hydroxy-6-hydroxymethyldihydropteridine pyrophosphokinase (AHHP)	1Q0N/P26281	**-9.4±0.0(-9.4)**
Prostaglandin-E2 9-reductase (PGE2 9-reductase)	1Q5M/P80508	**-9.7±0.0(-12.7)**
Phospholipase A1 (PLA1)	1QD6/P0A921	**-9.2±0.0(-9.7)**
Pyruvate Decarboxylase (PDC)	1QPB/P06169	**-9.2±0.0(-10.7)**
Renin	1RNE/ P00797	**-9.0±0.0(-10.0)**
NRH dehydrogenase [quinone] 2 (NDH2)	1SG0/P16083	**-9.3±0.0(-9.4)**
Aldehyde Reductase (ALR)	1T41/P15121	**-9.6±0.0(-10.5)**
Tubulin	1TUB/P02550	**-11.0±0.0(-9.1)**
D-amino acid oxidase (D-AAO)	1VE9/P00371	**-9.4±0.0(-10.7)**
Aldehyde oxidoreductase (AOR)	1VLB/Q46509	**-9.9±0.0(-10.0)**
Lanosterol Synthase (LS)	1W6K/P48449	**-9.5±0.0**(-13.1)
Ras-Ras Gap	1WQ1/P01112	**-9.3±0.0(-11.3)**
Epidermal Growth Factor Receptor (EGFR)	1XKK/P00533	**-9.3±0.0(-9.5)**
CLK1 protein kinase (CLK1)	1Z57/P49759	**-9.2±0.0(-8.7)**
Voltage gated potassium channel (VGKC)	1ZSX/Q13303	**-9.6±0.0(-9.9)**
Tropinone Reductase-II (TR-II)	2AE2/P50163	**-9.2±0.0(-13.2)**
Glutamate carboxypeptidase II (GCPII)	2C6C/Q04609	**-9.9±0.0(-7.6)**
UDP-N-Acetylmuramate dehydrogenase (UDP-N-ADH)	2MBR/P08373	**-9.0±0.0(-12.9)**
Medium chain acyl CoA dehydrogenase (MCAD)	3MDE/P41367	**-9.3±0.0(-9.3)**
Cyclooxygenase-2 (COX-2)	6COX/Q05769	**-9.4±0.0(-9.4)**
Insulin receptor pathway proteins		
PKA C-alpha	2QCS/P05132	**-9.0±0.0(-8.8)**
FOXO1	3CO6/Q12778	**-9.0±0.0(-9.1)**
Serum proteins		
Topo1	1A35/P11387	**-9.0±0.0(-9.0)**
Breast cancer proteins		
TOP2A	1ZXM/P11388	**-9.4±0.0(-10.1)**
Beta-2 adrenoceptor (beta(2)AR)	3NY8/P07550	**-9.8±0.0(-8.0)**

Table 38. Results of the validation experiments for trans-TR with AutoDock Vina.

Target name	PDB/UniProtKB	Affinity (kcal/mol)
Nuclear Receptor		
Lac Repressor (LacR)	1EFA/P03023	**-9.0±0.0**(-9.0）
Thyroid hormone receptor Beta-1 (TRβ1)	1NAX/P10828	**-10.7±0.0(-10)**
Receptors		
Retinoid X Receptor-alpha (RXRα)	1DKF/P10276	**-9.0±0.0(-16.0)**

Metabotropic Glutamate Receptor 2 (mGluR2)	1P1N/P19491	**-9.5±0.0(-8.1)**
Monoclonal Antibodies		
Immunoglobulin lambda Light Chain Dimer (Mcg) (IGL)	1MCJ	**-9.0±0.0(-9.5)**
Structural Proteins		
Myosin light chain kinase family (MLCK)	2BKH/Q29122	**-9.4±0.0(-9.5)**
Signaling Proteins		
Ran-GPPNHP-RanBP1-RanGAP	1K5D/P62826	**-9.2±0.0(-9.2)**
Lipid Binding Protein		
KES1 protein (KES1)	1ZHY/P35844	**-9.1±0.0(-11.1)**
Enzymes		
Acetylcholinesterase	1ACJ/P04058	**-9.2±0.0(-9.5)**
Fructose biphosphatase (FBP)	1BIF/P25114	**-9.5±0.0(-9.6)**
Lumazine Synthase (LS)	1C41/Q9UVT8	**-9.3±0.0(-7.7)**
Hypoxanthine-Guanine Phosphoribosyltransferase (HPRT)	1D6N/P00492	**-9.4±0.0(-8.3)**
Dihydrofolate Reductase (DHFR)	1DIS/P00381	**-9.3±0.0(-8.6)**
beta-Glucosidase (β-GS)	1E1F/P49235	**-9.9±0.0 (-9.1)**
Bovine Mitochondrial F1-ATPase (BMF1)	1EFR/P19483	**-9.6±0.0 (-9.7)**
Carboxylase	1FIY/P00864	**-9.4±0.0(-5.2)**
Xanthine dehydrogenase (XDH)	1FO4/P80457	**-9.3±0.0(-8.1)**
Cell Division Protein Kinase 2 (CDK2)	1GIH/P24941	**-9.1±0.0(-10.6)**
Monoamine Oxidase (MAO)	1GOS/P27338	**-9.4±0.0(-10.1)**
Spinach glycolate oxidase (SGO)	1GOX/P05414	**-9.4±0.0(-12.9)**
Prophospholipase A2 (proPLA2)	1HN4/P00592	**-9.0±0.0(-7.5)**
Adenosinetriphosphatase (ATPase)	1HPM/P19120	**-9.5±0.0(-9.7)**
Methionine decarboxylase (MetDC)	1I7B/P17707	**-9.3±0.0(-7.7)**
Cholesterol Oxidase (ChOx)	1IJH/P12676	**-9.1±0.0(-13.1)**
cAMP-Dependent Protein Kinase, Alpha-Catalytic Subunit (PKA)	1JBP/P05132	**-9.2±0.0(-8.2)**
Histamine N-methyltransferase (HNMT)	1JQE/P50135	**-9.2±0.0(-7.6)**
Aromatic-L-amino-acid Decarboxylase (AADC)	1JS3/P80041	**-9.1±0.0(-10.4)**
Casein Kinase II, Protein Kinase Ck2 (CK2)	1LP4/P28523	**-9.7±0.0(-7.9)**
Guanylate kinase (GK)	1LVG/Q64520	**-9.0±0.0(-9.9)**
NADPH Dehydrogenase (NDH)	1OYA/Q02899	**-9.1±0.0(-13.2)**
Riboflavin Kinase (RFK)	1P4M/Q969G6	**-9.3±0.0(-9.4)**
N, N-Dimethylglycine Oxidase (N, N-DMGO)	1PJ6/Q9AGP8	**-9.6±0.0(-11.7)**
DNA Topoisomerase II (topo II)	1PVG/P06786	**-9.4±0.0(-10.8)**
Prostaglandin G/H Synthase 2 (PGHS)	1PXX/Q05769	**-9.1±0.0(-9.2)**
2-amino-4-hydroxy-6-hydroxymethyldihydropteridine pyrophosphokinase (AHHP)	1Q0N/P26281	**-9.0±0.0(-9.4)**
Prostaglandin-E2 9-reductase (PGE2 9-reductase)	1Q5M/P80508	**-9.9±0.0(-12.7)**
Phospholipase A1 (PLA1)	1QD6/P0A921	**-9.7±0.0(-9.7)**
Pyruvate Decarboxylase (PDC)	1QPB/P06169	**-9.8±0.0(-10.7)**
Renin	1RNE/ P00797	**-9.2±0.0(-10.0)**
Protoporphyrinogen oxidase (PPO)	1SEZ/O24164	**-9.7±0.0(-14.2)**
Adenylosuccinate synthetase (AdSS)	1SON/P0A7D4	**-9.0±0.0(-9.1)**
Aldehyde Reductase (ALR)	1T41/P15121	**-9.5±0.0(-10.5)**
Tubulin	1TUB/P02550	**-11.2±0.0(-9.1)**
Aldose Reductase (AR)	1US0/P15121	**-9.1±0.0(-8.4)**
D-amino acid oxidase (D-AAO)	1VE9/P00371	**-9.6±0.0(-10.7)**
Lanosterol Synthase (LS)	1W6K/P48449	**-10.1±0.0(-13.1)**
Ras-Ras Gap	1WQ1/P01112	**-9.1±0.0(-11.3)**

Epidermal Growth Factor Receptor (EGFR)	1XKK/P00533	**-9.4±0.0(-9.5)**
Voltage gated potassium channel (VGKC)	1ZSX/Q13303	**-9.8±0.0(-9.9)**
WASP interacting protein (WIP)	2A3Z/P68135	**-9.2±0.0(-9.2)**
Tropinone Reductase-II (TR-II)	2AE2/P50163	**-9.0±0.0(-13.2)**
Glutamate carboxypeptidase II (GCPII)	2C6C/Q04609	**-9.7±0.0(-7.6)**
Cytochrome P450 2D6 (CYP2D6)	2F9Q/P10635	**-9.1±0.0(-14.6)**
UDP-N-Acetylmuramate dehydrogenase (UDP-N-ADH)	2MBR/P08373	**-9.3±0.0(-12.9)**
Medium chain acyl CoA dehydrogenase (MCAD)	3MDE/P41367	**-9.3±0.0(-9.3)**
Cyclooxygenase-2 (COX-2)	6COX/Q05769	**-9.1±0.0(-9.4)**
Insulin receptor pathway proteins		
PKA C-alpha	2QCS/P05132	**-9.5±0.0(-8.8)**
Serum proteins		
SL-2	1Q3A/P09238	**-9.2±0.0(-9.2)**
Breast cancer proteins		
TOP2A	1ZXM/P11388	**-9.2±0.0(-10.1)**
Beta-2 adrenoceptor (beta(2)AR)	3NY8/P07550	**-9.2±0.0(-8.0)**

Table S39. Interacting residues between CDT and proteins with the best affinity score.

Protein	Contact residues	Type of interactions
Intestinal Fatty Acid Binding Protein (IFABP)	Tyr14, Glu51, Val60, Tyr70, Leu72, Trp82, Phe93, Leu102, Ala104, Gln115, Tyr117, Arg126	Hydrophobic Interactions<br>Electrostatic Interactions<br>Aromatic Ring Interactions<br>Hydrogen Bond Donor<br>Hydrogen Bond Acceptor
Hypoxanthine-Guanine Phosphoribosyltransferase (HPRT)	Ala68a, Ser103a, Tyr104a, Glu133a, Asp134a, Ile135a, Thr138a, Gly139a, Thr141a, Lys165a, Val187a, Leu192a, Asp193a	Hydrophobic Interactions<br>Electrostatic Interactions<br>Hydrogen Bond Donor<br>Hydrogen Bond Acceptor
Dihydrofolate Reductase (DHFR)	Ile13, Leu19, Asp26, Leu27, Phe30, Thr45, Phe49, Thr126	Hydrophobic Interactions<br>Electrostatic Interactions<br>Hydrogen Bond Donor<br>Hydrogen Bond Acceptor

beta-Glucosidase (β-GS)	His142a, Trp143a, Glu191a, Thr194a, Phe198a, Phd205a, Asp261a, Met263a, Trp378a, Glu406a, Trp457a, Trp465a	Hydrophobic Interactions<br>Electrostatic Interactions<br>Aromatic Ring Interactions<br>Hydrogen Bond Donor<br>Hydrogen Bond Acceptor
Carboxylase	Glu86, Arg587, Gly589, Ile626, Ser629, Ser630,Lys773, Trp824, Ile825, Arg832, Arg880, Asn881	Hydrophobic Interactions<br>Electrostatic Interactions<br>Aromatic Ring Interactions<br>Hydrogen Bond Donor<br>Hydrogen Bond Acceptor
Glycogen Phosphorylase B (GPB)	Tyr90a, Asp283a, Asn284a, Arg569a, Lys574a, Lys608a, Tyr648a, Glu672a, Lys680a	Hydrophobic Interactions<br>Electrostatic Interactions<br>Aromatic Ring Interactions<br>Hydrogen Bond Donor<br>Hydrogen Bond Acceptor
Acetylcholinesterase	Tyr70a, Trp84a, Tyr121a, Ser122a, Ser200a, Phe330a, His440a	Hydrophobic Interactions<br>Electrostatic Interactions<br>Hydrogen Bond Donor<br>Hydrogen Bond Acceptor
Polyamine oxidase (PAO)	Arg43, Als58, Trp60, Glu62, Glu170, Tyr298, Lys300, Thr402, Phe403, Glu430, Gly438, Tyr439, Val440, Ala443	Hydrophobic Interactions<br>Electrostatic Interactions<br>Hydrogen Bond Donor<br>Hydrogen Bond Acceptor
Prophospholipase A2 (proPLA2)	Phe5a, Gly32a, His48a, Tyr52a, Asp99a	Hydrophobic Interactions<br>Electrostatic Interactions

		Hydrogen Bond Donor<br>Hydrogen Bond Acceptor
Casein Kinase II, Protein Kinase Ck2 (CK2)	Arg43a, Val45a, Val53a, Ile66a, Lys68a, Tyr115a, Val116a, Met163a, Ile174a, Asp175a, Trp176a	Hydrophobic Interactions<br>Electrostatic Interactions<br>Hydrogen Bond Donor<br>Hydrogen Bond Acceptor
Tubulin	Gln8, Gly17, Trp21, Ile24, Gln43, Leu44, Glu47, Arg48, Val62, Phe83, Ile86	Hydrophobic Interactions<br>Electrostatic Interactions<br>Hydrogen Bond Donor<br>Hydrogen Bond Acceptor
CLK1 protein kinase (CLK1)	Phe172, Val175, Lys191, Val225, Phe241, Leu244, Ser247, Asn293, Leu295, Val324	Hydrophobic Interactions<br>Hydrogen Bond Donor<br>Hydrogen Bond Acceptor
Glutamate carboxypeptidase II (GCPII)	Arg210, His377, Asp387, Glu425, Asp453, Tyr552, His553, Tyr700	Hydrophobic Interactions<br>Electrostatic Interactions<br>Aromatic Ring Interactions<br>Hydrogen Bond Acceptor
PKA C-alpha	Thr51a, Ser53a, Val57a, Gly96a, Ala97a, Glu127a, Lys168a, Leu173a, Thr183a, Phe327a	Hydrophobic Interactions<br>Electrostatic Interactions<br>Hydrogen Bond Donor<br>Hydrogen Bond Acceptor
Beta-2 adrenoceptor (beta(2)AR)	Trp109a, Thr110a, Asp113a, Val114a, Val117a, Phe193a, Ser203a, Phe289a, Phe290a, Asn312a	Hydrophobic Interactions<br>Electrostatic Interactions<br>Hydrogen Bond Donor

Table S40. Interacting residues between TDT and proteins with the best affinity score.

Protein	Contact residues	Type of interactions
Thyroid hormone receptor Beta-1 (TRβ1)	Phe272a, Ile275a, Ile276a, Ala279a, Met310a, Met313a, Arg320a, Thr329a, Leu330a, Leu341a, Gly344a, Leu346a	Hydrophobic Interactions<br>Electrostatic Interactions<br>Hydrogen Bond Donor<br>Hydrogen Bond Acceptor
Metabotropic Glutamate Receptor 2 (mGluR2)	Ile11a, Glu13a, Lys60a, Tyr61a, Thr91a, Arg96a, Thr138a, Ser140a, Ser142a, Arg172a, Thr173a, Thr174a, Tyr190a, Glu193a, Met196a	Hydrophobic Interactions<br>Electrostatic Interactions<br>Hydrogen Bond Donor<br>Hydrogen Bond Acceptor
Lumazine Synthase (LS)	Asp179a, Trp180a, Trp25b, Ser59b, Trp60b, Leu120b, His127b, Ile131b	Hydrophobic Interactions<br>Electrostatic Interactions<br>Aromatic Ring Interactions<br>Hydrogen Bond Donor<br>Hydrogen Bond Acceptor
Hypoxanthine-Guanine Phosphoribosyltransferase (HPRT)	Ala68a, Ser103a, Tyr104a, Glu133a, Asp134a, Ile135a, Asp137a, Thr138a, Gly139a, Thr141a,	Hydrophobic Interactions<br>Electrostatic Interactions

	Lys165a, Val187a, Phe186a, Leu192a, Asp193a	Hydrogen Bond Donor<br>Hydrogen Bond Acceptor
Dihydrofolate Reductase (DHFR)	Ile13, His18, Leu19, Asp26, Leu27, Phe30, Thr45, Ser48, Phe49, Ala97	Hydrophobic Interactions<br>Electrostatic Interactions<br>Hydrogen Bond Donor<br>Hydrogen Bond Acceptor
beta-Glucosidase (β-GS)	His142a, Trp143a, Asn190a, Glu191a, Thr194a, Phe205a, Asp261a, Met263a, Arg265a, Glu406a, Trp465a, Tyr473a	Hydrophobic Interactions<br>Electrostatic Interactions<br>Hydrogen Bond Donor<br>Hydrogen Bond Acceptor
Carboxylase	Gly589, Ile626, Ser629, Ser630, Lys773, Trp824, Ile825, Arg832, Arg880, Asn881	Hydrophobic Interactions<br>Aromatic Ring Interactions<br>Electrostatic Interactions<br>Hydrogen Bond Donor<br>Hydrogen Bond Acceptor
Xanthine dehydrogenase (XDH)	Phe798, Arg912, Met1038, Lys1045, Thr1077,	Hydrophobic Interactions

	Thr1083, Val1259, Gly1260	Electrostatic Interactions<br>Hydrogen Bond Donor<br>Hydrogen Bond Acceptor
Prophospholipase A2 (proPLA2)	Leu2b, Phe5b, Ile9b, Pro18b, Asn23b, Gly30b, His48b, Asp49b, Phe106b	Hydrophobic Interactions<br>Electrostatic Interactions<br>Hydrogen Bond Donor<br>Hydrogen Bond Acceptor
Leukotriene A4 hydrolase (LTA4H)	Gln136, Ala137, Tyr267, Phe314, Leu369, Asp375, Ala377, Tyr378, Ser379	Hydrophobic Interactions<br>Electrostatic Interactions<br>Hydrogen Bond Donor<br>Hydrogen Bond Acceptor
Methionine decarboxylase (MetDC)	Lys80a, Cys82a, Phe223a, Cys226a, Thr245a, Glu247a, Phe7b, Ser66b, Glu67b	Hydrophobic Interactions<br>Electrostatic Interactions<br>Hydrogen Bond Donor<br>Hydrogen Bond Acceptor
cAMP-Dependent Protein Kinase, Alpha-	Leu49e, Phe54e, Val57e, Lys72e, Val123e,	Hydrophobic Interactions

Catalytic Subunit (PKA)	Glu127e, Leu173e, Thr183e, Phe327e	Electrostatic Interactions<br>Hydrogen Bond Donor<br>Hydrogen Bond Acceptor
Histamine N-methyltransferase (HNMT)	Tyr15a, Ser18a, Tyr146a, Trp179a, Cys196a, Asp242a, Phe243a	Hydrophobic Interactions<br>Electrostatic Interactions<br>Hydrogen Bond Donor<br>Hydrogen Bond Acceptor
Casein Kinase II, Protein Kinase Ck2 (CK2)	Arg47a, Ser51a, Val53a, Ile66a, Lys68a, Val95a, Phe113a, Glu114a, Val116a, Ile174a, Asp175a	Hydrophobic Interactions<br>Electrostatic Interactions<br>Hydrogen Bond Donor<br>Hydrogen Bond Acceptor
Tubulin	Gln8, Gly17, Trp21, Ile24, Gln43, Leu44, Glu47, Arg48, Val62, Phe83, Ile86	Hydrophobic Interactions<br>Electrostatic Interactions<br>Hydrogen Bond Donor<br>Hydrogen Bond Acceptor
Aldose Reductase (AR)	Trp20, Lys21, Asp43, Tyr48, Lys77, His110,	Hydrophobic Interactions

	Trp111, Gln183, Tyr209, Ser214, Ile260, Lys262	Electrostatic Interactions<br>Hydrogen Bond Donor<br>Hydrogen Bond Acceptor
Glutamate carboxypeptidase II (GCPII)	Arg210, His377, Asp387, Glu425, Asp453, Tyr552, His553, Tyr700	Hydrophobic Interactions<br>Electrostatic Interactions<br>Hydrogen Bond Donor<br>Hydrogen Bond Acceptor
PKA C-alpha	Thr51a, Ser53a, Val57a, Gly96a, Ala97a, Val123a, Glu127a, Lys168a, Leu173a, Thr183a, Phe327a	Hydrophobic Interactions<br>Electrostatic Interactions<br>Hydrogen Bond Donor<br>Hydrogen Bond Acceptor
Beta-2 adrenoceptor (beta(2)AR)	His93a, Trp109a, Thr110a, Asp113a, Val114a, Val117a, Ser203a, Ser204a, Phe289a, Phe290a, Asn312a, Trp313a, Tyr316a	Hydrophobic Interactions<br>Electrostatic Interactions<br>Hydrogen Bond Donor<br>Hydrogen Bond Acceptor
